# Improved Longan Genome Assembly Reveals Insights Into Flowering Mechanisms

**DOI:** 10.1111/pbi.70433

**Published:** 2025-11-04

**Authors:** Guochun Zhao, Fan Jiang, Wenshun Hu, Jisen Zhang, Wen Wang, Siping Tu, Baiyu Wang, Qing Zhang, Jing Mei, Lianyu Lin, Yiying Qi, Xiuping Chen, Jianguo Li, Ray Ming, Shaoquan Zheng

**Affiliations:** ^1^ Center for Genomics and Biotechnology, Fujian Provincial Key Laboratory of Haixia Applied Plant Systems Biology, Key Laboratory of Genetics, Breeding and Multiple Utilization of Corps, Ministry of Education Fujian Agriculture and Forestry University Fuzhou Fujian China; ^2^ Fujian Breeding Engineering Technology Research Center for Longan and Loquat, Fruit Research Institute Fujian Academy of Agricultural Sciences Fuzhou Fujian China; ^3^ State Key Lab for Conservation and Utilization of Subtropical Agro‐Biological Resources, Guangxi Key Lab for Sugarcane Biology, College of Agriculture Guangxi University Nanning China; ^4^ State Key Laboratory for Conservation and Utilization of Subtropical Agro‐Bioresources, Guangdong Litchi Engineering Research Center South China Agricultural University Guangzhou China

**Keywords:** crop improvement, *Dimocarpus longan*, floral induction, KClO_3_, Sapindaceae

## Abstract

Longan is an exotic tropical fruit crop and exhibits off‐season flowering induced by potassium chlorate (KClO_3_), though the molecular mechanisms remain unclear. We assembled a high‐quality, 441.5 Mb genome of variety ‘Shixia’, with a contig N50 at 28.1 Mb, 29, 325 protein‐coding genes, 26 telomeres and 15 centromeres. Comparative genomic analysis with lychee revealed structural variations potentially driving gene family expansions related to flavone biosynthesis and disease resistance. Transcriptomic profiling showed that natural flowering appears to be primarily regulated by photoperiod, vernalisation and autonomous pathways, while KClO_3_‐induced flowering may preferentially activate the gibberellin pathway at 5 days after treatment (DAT) and autonomous pathways at 10 DAT, involving putative repression of *DlDDF1*, *DlFLCs* and *DlSVPs*, and up‐regulation of *DlWRKY75_2*. In the perpetual‐flowering variety ‘Sijimi’, elevated expression of photoperiod genes, such as *DlCOR28*, *DlCOR27*, *DlADO3*, *DlPRR5*, *DlGI* and *DlJMJ30*, may explain its perpetual blooming. Overexpression of *DlDDF1* in Arabidopsis delayed flowering, partially reversed by KClO_3_ through an increase in bioactive GA_4_. Together, KClO_3_‐induced flowering likely involves oxidative stress response and gibberellin signalling via *DlDDF1* repression, while natural flowering relies on seasonal cues. These results lay the foundation for longan genetic improvement.

## 1 Introduction

Longan ( *Dimocarpus longan* Lour.) is a member of the Sapindaceae family, which contains 138 genera with 1858 species in four subfamilies and 20 tribes (Buerki et al. 2021). This family includes valuable fruit crop trees such as longan, lychee (Litchi *chinensis* Sonn.), rambutan ( *Nephelium lappaceum* L.), soapberry ( *Sapindus mukorossi* Gaertn.), yellowhorn ( *Xanthoceras sorbifolia* Bunge), maple (*Acer* L.), ackee ( *Blighia sapida* K.D. Koenig) and horse chestnut ( *Aesculus hippocastanum* L.). Because of their economic importance, draft and high‐quality genomes have been published for yellowhorn (Wang et al. 2023), lychee (Hu et al. 2022), soapberry (Xue et al. 2022), rambutan (Zhang et al. 2021) and *Acer* spp. (Chen et al. 2023; Yang et al. 2019; Yu et al. 2021). These genomic resources are readily accessible for studying the evolution of the Sapindaceae family and the gene regulation networks underlying flowering and economically important traits.

Longan originated in southern China, where it has been cultivated for over 2000 years. Its first recorded use dates back to the Han Dynasty, after which it spread to other regions through trade. The name ‘longan’, meaning ‘dragon eye’ in Chinese, is derived from its appearance when shelled, resembling the eyeball of a mythical dragon. Now, longan is widely cultivated in subtropical and tropical regions worldwide. Major production countries include China, Thailand, Vietnam, Malaysia, Indonesia, Australia, India, Sri Lanka, Israel and the United States. China leads global longan production, accounting for 50% of the world's output, with the largest cultivated area, highest fruit yield and the most diverse varieties, followed by Thailand and Vietnam (South Subtropical Crops Center 2024). Longan is widely valued for its unique flavour and medicinal properties. The fruit is sweet, juicy and commonly consumed fresh, while dried longan is in high demand and widely used in the food industry. Nutritionally, longan pulp is rich in vitamin C, minerals, polysaccharides, antioxidants and dietary fibre. It plays an important role in traditional Chinese medicine and modern medical practices, offering benefits in treating insomnia, forgetfulness, heart palpitations and exhibiting anticancer, anti‐inflammatory and immune‐regulating properties (Zeng et al. 2024).

Off‐season flowering induced by potassium chlorate (KClO_3_) is a major breakthrough in longan cultivation, substantially enhancing its year‐round production (Yan et al. 1998). However, conventional breeding for genetic improvement in longan remains challenging due to its lengthy juvenile phase of 7–8 years. Only the draft genome of the variety ‘Honghezi’ (Lin et al. 2017) and the chromosome‐scale genome of ‘Jidanben’ (Wang, Li, et al. 2022; Wang, Wang, et al. 2022) have been generated using Illumina sequencing and PacBio single molecule real‐time (SMRT) reads, respectively. ‘Shixia’ is the leading variety known for its extensive cultivation area, high yield, crisp and refreshing flesh, high sugar content and pleasant aroma. It is also highly sensitive to KClO_3_‐induced flowering, yet its genome has not been deciphered.

Floral induction is a key determinant of longan yield. Under natural conditions, longan blooms only once a year, following a period of low‐temperature stimulation during winter (Pham et al. 2015). However, some exceptions exist, such as the variety ‘Sijimi’, which can achieve perpetual flowering (PF) under optimal conditions (Jue et al. 2019). In tropical regions, the application of KClO_3_ can induce flowering in longan during any season. The use of KClO_3_ substantially expanded the cultivation area and increased longan production, particularly for off‐season production (Hau and Hieu 2019). Understanding the regulatory gene networks underlying flowering across different varieties, under both natural and KClO_3_‐induced conditions, is crucial for advancing breeding and elucidating flowering regulation in tropical and subtropical fruit trees.

## 2 Results

### 2.1 Genome Assembly and Annotation

**FIGURE 1:**
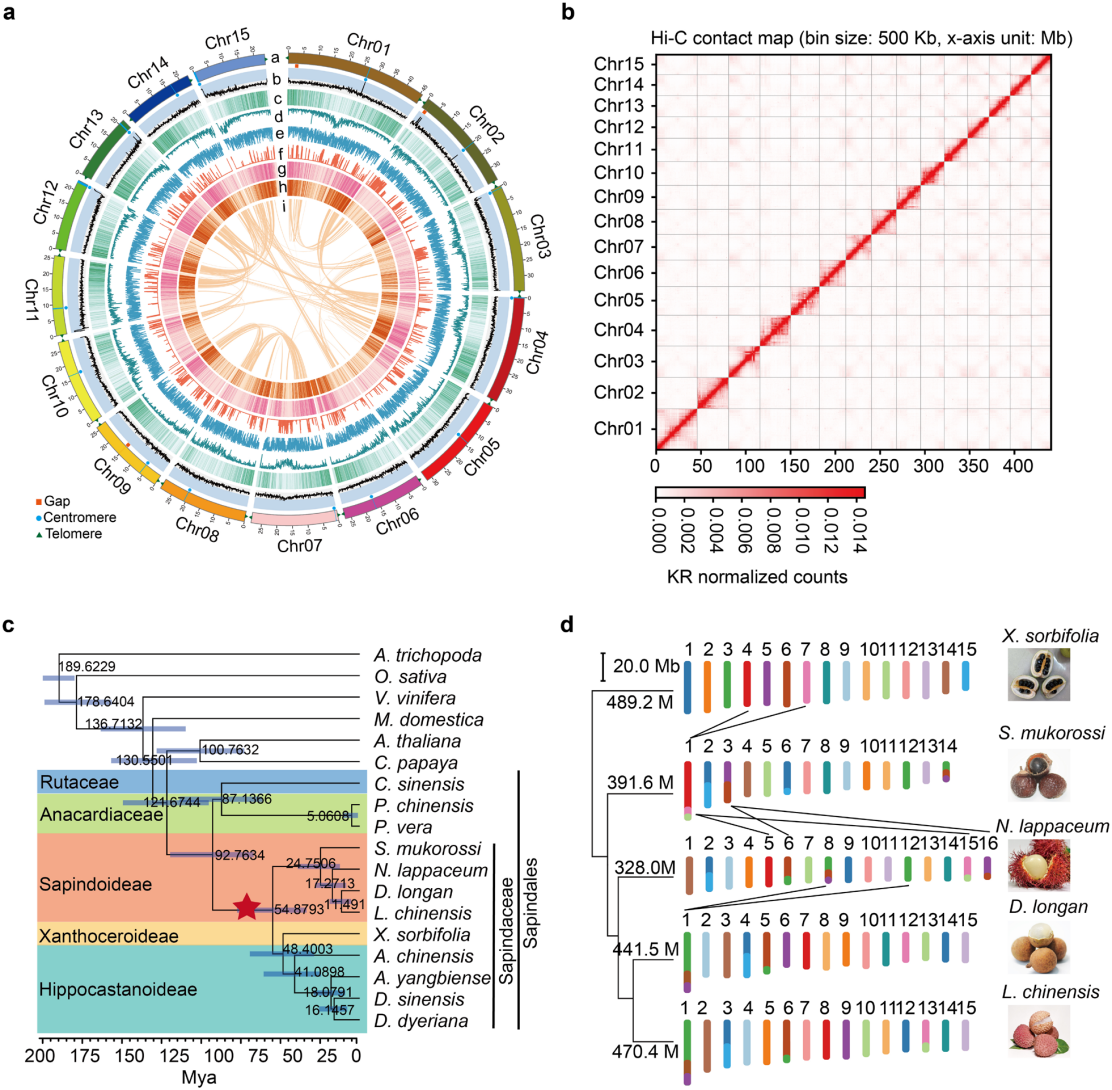
Genomic features, Hi‐C contact maps and karyotype evolution of longan ‘Shixia’. (a) Genomic feature distribution across 15 pseudo‐chromosomes. Rings (outermost to innermost) show: a, Chromosome structure with predicted centromeres, telomeres and assembly gaps; b, GC content; c, gene density; d, Copia‐type TE density; e, Gypsy‐type TE density; f, SNP density; g, gene expression in natural flowering; h, gene expression in KClO_3_‐induced flowering; i, collinear block links. (b) Hi‐C intrachromosomal contact maps of 15 pseudo‐chromosomes, where contact intensity between 500 kb windows is indicated on a logarithmic scale; darker red represents higher contact probability. (c) Evolutionary relationships of 18 species, including members of the Sapindaceae family across Sapindoideae, Xanthoceroideae and Hippocastanoideae subfamilies, alongside model plant species. (d) Karyotype evolution among four closely related genera. Karyotype colours reflect collinearity with yellowhorn.

The genome size of the longan variety ‘Shixia’ was estimated at 441.5 Mb (Figure 1a). For assembly, we generated 22.3 Gb of PacBio HiFi reads (51× coverage) and 88.1 Gb Hi‐C reads (200× coverage) (Table S1). Using HiFiasm, we assembled HiFi reads into 98 contigs (N50 = 28.3 Mb), totalling 441.5 Mb (Figure S1, Tables S2 and S3). Using HiC‐Pro and EndHiC, 437.8 Mb (99.2%) of the assembly was anchored to 15 pseudo‐chromosomes (Figure 1b, Figure S2, Tables S4 and S5).

The assembly reached high quality with 98.4% BUSCO completeness, a quality value (QV) of 68.1 and a HiFi read coverage rate of 99.99% (Tables S6–S9). We annotated 29,325 protein‐coding genes with 97.7% BUSCO completeness, fewer than in ‘Jidanben’ (40,420 genes) and ‘Honghezi’ (31,007 genes) (Tables S10 and S11), likely due to improved assembly accuracy. The contig N50 increased from 12.1 to 28.3 Mb, nearly matched the average chromosome length of 29.2 Mb, with 26 telomeres and 15 centromeres predicted, and only three gaps on chromosomes 1, 2 and 9 (Figure 1a, Tables S12–S14, Figure S3). Additionally, we identified 137 microRNAs, 885 snRNAs, 437 tRNAs and 1627 rRNAs (Table S15), with 96.4% of genes functionally annotated using nine databases (Table S16).

Transposable elements (TEs) comprised 55.0% of the assembled genome. Class I elements (retrotransposons) accounted for 33.0%, including 21.1% long terminal repeat retrotransposons (LTR‐RTs) and 7.2% non‐long terminal repeat retrotransposons (nLTR‐RTs). Class II elements (DNA transposons) represented 20.0%, primarily consisting of 18.9% terminal inverted repeat (TIR) elements (Table S17).

### 2.2 Comparative Genomic Analysis

**FIGURE 2:**
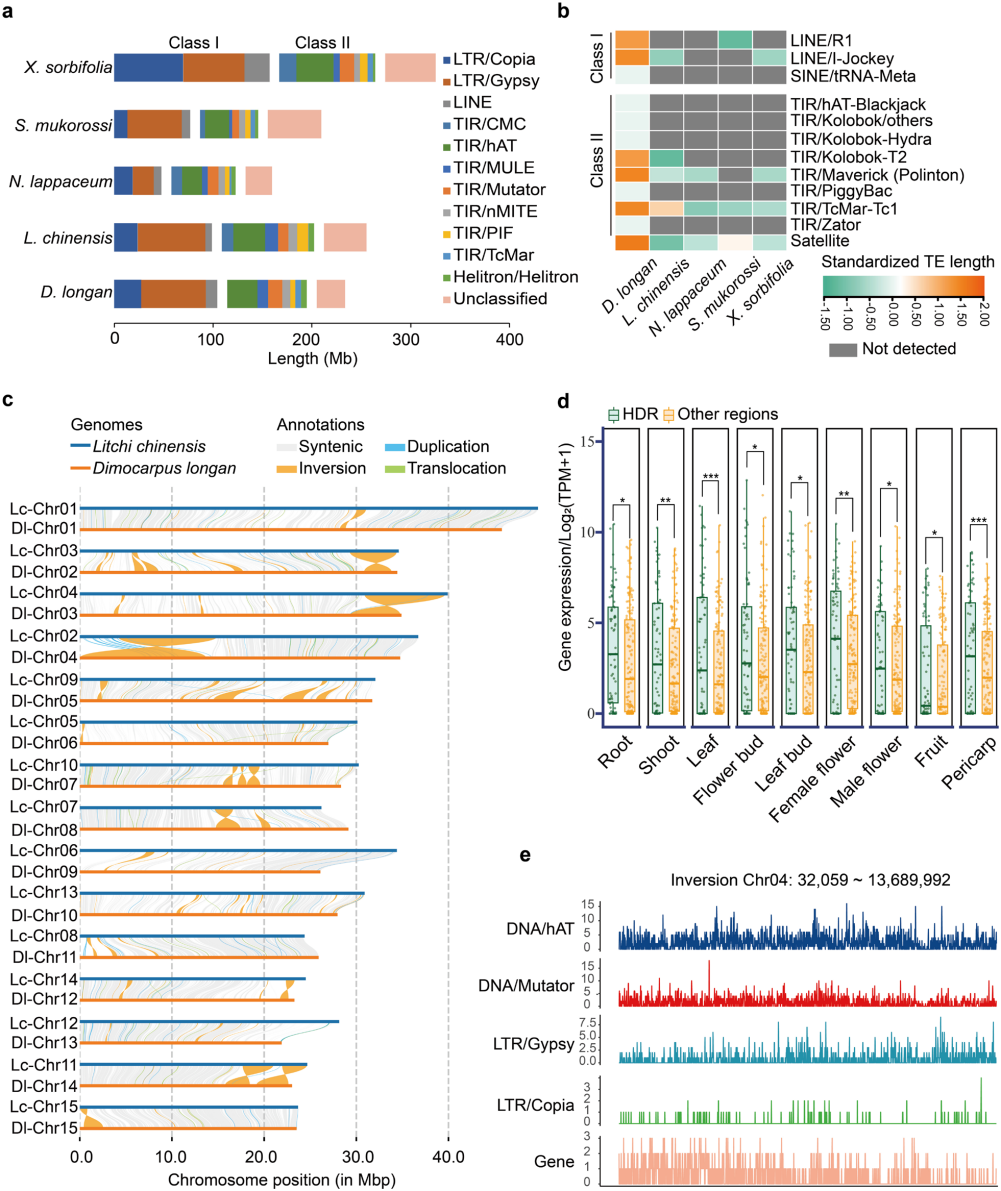
Comparative genomic analysis of transposable elements and structural variations in longan and lychee. (a) Comparison of transposable elements (TEs) types and proportions across five species from closely related genera. (b) Specifically enriched and abundantly distributed TEs in longan. The colour gradient reflects TE proportions, with orange indicating higher values. (c) Structural variation analysis between homologous chromosomes of longan and lychee. (d) Comparison of flavone biosynthesis‐related gene expression levels in highly diverged regions (HDRs) and other regions across different tissues. Differential analysis was performed using a *t*‐test, with *, ** and *** indicating *p*‐values < 0.05, 0.01 and 0.001, respectively. (e) Distribution density of more abundant TEs and genes in 10‐kb windows within a larger inversion region (Chr04: 32 059–13 689 992) of longan.

Phylogenetic and syntenic analyses of longan and four related species revealed a close phylogenetic relationship and high collinearity (Figures S4–S10, Figure 1c,d). Chromosome numbers varied from 14 to 16 due to fusion and fission events, with soapberry at 14, lychee, longan and yellowhorn at 15, and rambutan at 16 (Figure 1d, Table S18). While non‐repetitive genome sizes were similar (~200 Mb) (Figure S11), total genome sizes varied from 328.0 to 489.2 Mb due to differences in repetitive sequences content (Figure 2a). Yellowhorn had the highest repetitive sequence portion (63.5%), dominated by *Copia* and LINEs (Table S11), whereas longan, lychee and soapberry had more *Gypsy* elements (Figure 2a). A recent *Copia* burst occurred in yellowhorn, as characterised by the lowest divergence and the latest insertions of LTR, LINE and pararetrovirus elements (Figures S12 and S13). Longan uniquely contained 12 TEs absent in the others, including two LINEs, one SINE, eight class II elements and one satellite (Figure 2b).

Comparative genomic analyses identified 486 longan‐specific gene families (comprising 3522 genes) and 3206 BlastP‐unique genes, enriched in pathways related to pathogen response, glutathione and sugar metabolism (Figures S14–S16, Table S19). Since diverging from lychee, 857 gene families have expanded and 1587 have contracted in longan, including 28 NBS‐encoding genes (Figure S17). NBS genes, more abundant in longan than lychee (Table S20), clustered mainly on chromosomes 2, 3 and 5 (Table S21, Figure S18). Longan‐specific expansion was enriched in flavone/flavonol biosynthesis, involving 27 significantly expressed genes (e.g., seven *chalcone stilbene synthases* (*CHS*) and five *Feruloyl‐CoA 6ʹ‐Hydroxylase* (*F6ʹH*)), higher expressed in flower buds (Figures S19 and S20).

Structural variation (SV) analysis between longan and lychee revealed 43.6% synteny (Table S22). Major SVs included inversions (INV) (55.4 Mb, 3000 genes), duplications (DUP) (9.6 Mb, 1926 genes) and translocations (TRANS) (3.0 Mb, 618 genes) (Figure 2c, Tables S22 and S23). Large INV regions were found on the ends of chromosomes 2, 3, 4 and 14 (Table S23), with genes in these regions enriched in plant‐pathogen interaction and flavone and flavonol biosynthesis pathways (Figures S21–S23). Highly diverged regions (HDR) (170.7 Mb, 39% of the genome) showed elevated gene expression of flavone biosynthesis genes across tissues (Figure 2d, Figure S24). Furthermore, 39, 30 and 7 floral pathway genes were identified within INV, DUP and TRANS, respectively, including *Flower Locus1* (*DlFT1*) and *Early Flowering 4* (*DlELF4*) in INVs, *Short Vegetative Phase* (*DlSVP4*) and *FD PARALOG* (*DlFDP*) in DUPs, and *DlSVP5/8* in TRANSs, suggesting potential roles of these SVs in floral regulation.

TEs were abundant in these SVs (INVs: 77669; DUPs: 7023; TRANs: 2776), with hAT, Mutator, LTR/*Gypsy* and LTR/*Copia* elements more prevalent overall (Table S24). In INVs, *Gypsy* elements (Tekay, Retand, CRM), LINEs (*I‐Jockey*, *L1*, *R1*) and *CMC‐EnSpm* elements were particularly prevalent (Figure 2e, Figure S25). Additionally, 12 longan‐specific repeats were enriched in HDR and INV regions, particularly LINE elements (*I‐Jockey*, *R1*) and *PiggyBac* elements (Figure S26).

### 2.3 Natural Floral Transition in Longan

**FIGURE 3:**
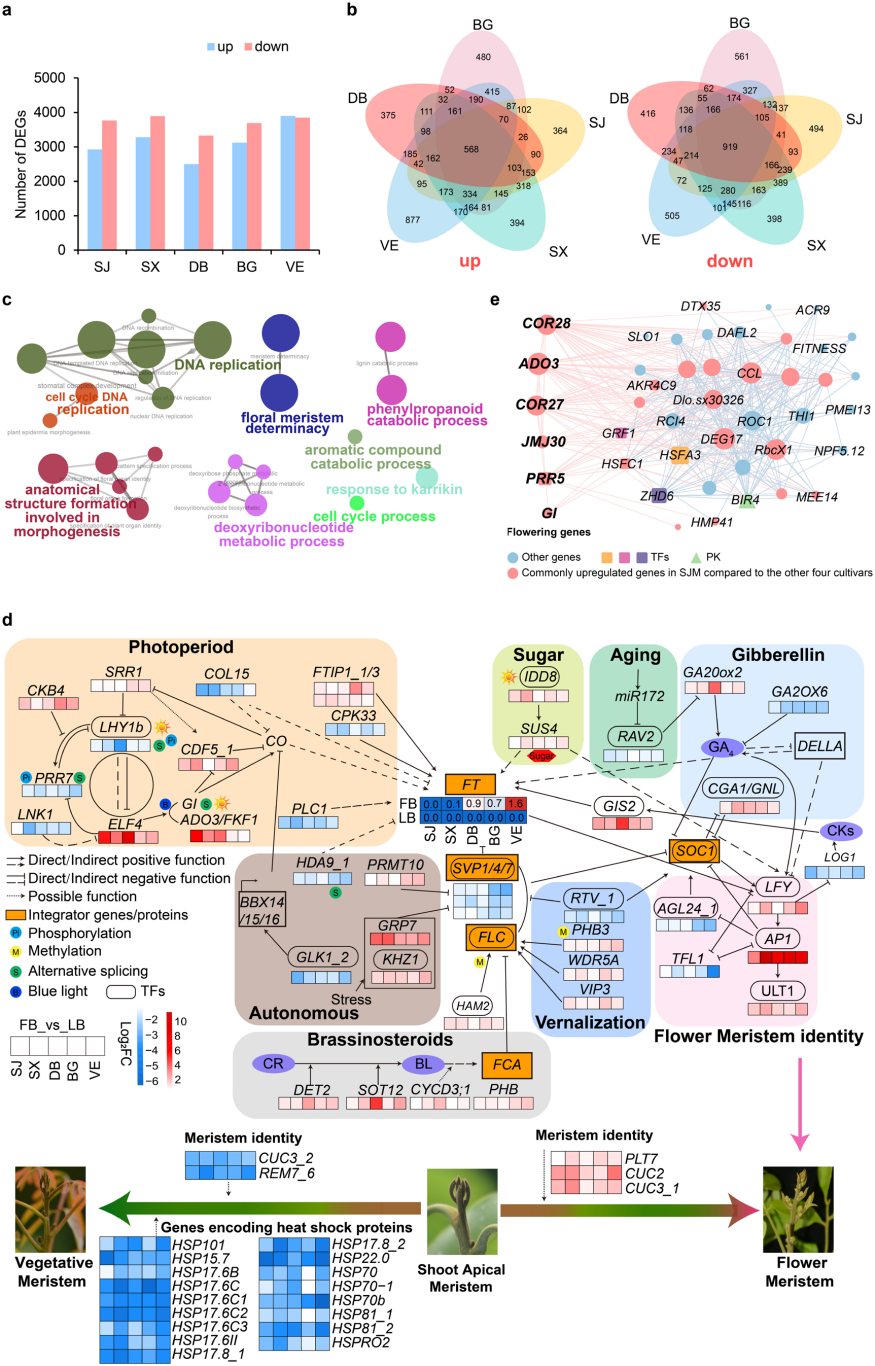
Transcriptome dynamics of natural floral transition in longan. (a) Pairwise comparisons of differentially expressed genes (DEGs) between the leaf bud and flower bud in five varieties ‘Dongbao NO. 9’ (‘DB’), ‘Siji’ (‘SJ’), ‘Shixia’ (‘SX’), ‘Biaogui’ (‘BG’) and ‘Vienna Erzhao’ (‘VE’). (b) Venn diagram showing the number of shared and unique DEGs between the leaf bud and flower bud across the five varieties. (c) GO enrichment analysis of 2642 uniquely up‐regulated DEGs (|log_2_FC| > 2, *FDR* < 0.05) in all five longan varieties. Nodes represent significantly enriched GO terms clustered based on functional similarity. (d) Integrated gene regulatory network (GRN) of key genes consistently up‐regulated or down‐regulated in flower buds across five varieties, involved in floral pathways, hormone signalling, meristem identity and heat shock response. Heatmaps display log_2_FC values for floral pathway genes except *DlFT1*, which is shown as transcripts per kilobase million (TPM) across different varieties and tissues. Arrows indicate positive regulation, while bars represent negative regulation. BL: Brassinolide; CR: Campesterol; CKs: Cytokinins. (e) Specific gene co‐expression network of floral transition for ‘Sijimi’ according to WGCNA analysis. The top 0.15% co‐expression relationships of the ‘Sijimi’ flower bud strongly correlated MEpink were used to construct this network in cytoscape (weight > 0.26, 47 genes).

Floral transition involves the conversion of the shoot apical meristem into a floral meristem, a critical process that determines stem identity and developmental fate. To uncover the molecular mechanisms underlying floral transition, we conducted RNA‐seq on flower and leaf buds from five longan varieties under natural on‐season conditions. We identified 13,673 differentially expressed genes (DEGs), with 6522 up‐regulated and 5922 down‐regulated in flower buds (Figure 3a). Of these, 568 up‐regulated and 919 down‐regulated DEGs were shared across five varieties (Figure 3b).

Weighted Gene Co‐expression Network Analysis (WGCNA) revealed strong co‐expression among shared up‐regulated DEGs, forming a high‐confidence network of 577 genes (Figure S27, weight > 0.87). Key hub genes included *ARSENIC TOLERANCE 5* (*DlARS5*), *NUCLEOTIDE TRANSPORTER 1* (*DlNTT1*), *PSEUDOURIDINE SYNTHASE 10* (*DlPUS1*), *DlGRF3* and 11 flowering genes. Most of these flowering genes belonged to the photoperiod and autonomous pathways (Figure S28). In contrast, down‐regulated genes consistently exhibited weaker co‐expression, and a relaxed threshold was used to obtain a meaningful network of 821 genes (weight > 0.45, Figure S29). Key hub genes included *DloSX03G010400*, *VIT tetratricopeptide‐repeat thioredoxin‐like 3* (*DlTTL3*), *ABA INSENSITIVE 1* (*DlABI1*) and *DlWRKY33_1*, along with 15 flowering genes (Figure S30). GO enrichment analysis revealed that up‐regulated DEGs were linked to floral meristem determinacy, morphogenesis, cell cycle DNA replication and phenylpropanoid catabolic processes (Figure 3c, Figure S31). In contrast, down‐regulated DEGs were associated with stress response, defence processes and negative regulation of flower development (Figures S32 and S33).

Using three flower time‐related databases, 577 floral pathway homologues were identified in longan, with 20 up‐regulated and 16 down‐regulated across five varieties. Gene regulatory networks (GRNs) inferred Arabidopsis floral pathways revealed that floral transition in longan involved photoperiod, autonomous and vernalisation pathways, interacting with gibberellin (GA) and sugar signalling (Figure 3d, Figure S31). Autonomous pathway genes *DlKHZ1* and *DlPRMT10* showed high connectivity, while *DlGRP7* was commonly up‐regulated (Figure S34a). In the vernalisation pathway, *DlPHB3*, *DlVIP3* and *DlWDR5A* exhibited strong co‐expression and elevated expression. Photoperiod pathway genes, *DlELF4* and *DlADO3*/*FKF1* were activated, whereas *DlLHY1b*, *DlPRR7* and *DlLNK1* were down‐regulated. Key repressors, including *DlSVP1*/*4*/*7* and *DlTFL1* were suppressed, with *DlSVP4* exhibiting the highest connectivity (Figure S34b). Concurrently, *DlFT1*, *DlAP1*, *DlLFY* and *DlULT1* were up‐regulated in flower buds (Figure 3d).

The cytokinin response gene *DlGIS2* and brassinosteroid pathway genes *DlDET2*, *DlSOT12* and *DlCYCD3;1* were up‐regulated (Figure 3d). Meristem identity genes (*DlCUC3_1*, *DlCUC2*, *DlPLT7*), transcription factors (TFs) and hormone‐related genes may also contribute to flower bud formation (Figures S35 and S36). In contrast, 17 heat shock protein genes (*DlHSPs*) were down‐regulated (Figure 3d, Figure S36a,b). Notably, *DlCUC3_1* and *DlCUC3_2* exhibited opposing expression.

The ever‐flowering variety ‘Sijimi’ flowers continuously between 5°C and 35°C. Among 2840 genes up‐regulated in flower buds (vs. leaf buds), 1176 were also more highly expressed in ‘Sijimi’ flower buds than in those of the other four varieties. Of these highly expressed genes in ‘Sijimi’, 53 in flower buds and 59 in leaf buds were common across the other four varieties (Figure S37a,b). These genes included six up‐regulated genes associated with the circadian clock and photoperiod pathway: *COLD REGULATED GENE 27* (*DlCOR27*), *DlCOR28*, *DlFKF1*/*ADO3*, *DlGI*, *DlPRR5* and *Jumonji C domain‐containing protein 30* (*DlJMJ30*) (Figure S37c,d). To further explore genes potentially involved in the unique floral transition of ‘Sijimi’, we conducted WGCNA using all the transcriptome profiles from flower and leaf buds across the five varieties. The MEpink module, comprising 692 genes, showed the strongest positive correlation with the ‘Sijimi’ flower bud (Figure S37e,f). Within this module, 47 genes formed a tightly connected co‐expression network, which appeared to be specific to ‘Sijimi’. Notably, six commonly and highly expressed flowering‐related genes were within this network, and *DlCOR27*, *DlCOR28* and *DlFKF1*/*ADO3* acted as hub genes, along with *ROTAMASE CYP 1* (*DlROC1*) and *BPG4 HOMOLOGOUS GENE 3* (*DlBGH3*) (Figure 3e). Several TFs, including *HEAT SHOCK* related (*DlHSFA3*, *DlHSFC1*), *GROWTH‐REGULATING FACTOR 1* (*DlGRF1*) and *ZINC FINGER HOMEODOMAIN 6* (*DlZHD6*), also contribute to this regulatory network.

### 2.4 KClO_3_ ‐Induced Off‐Season Flowering in Longan

KClO_3_‐induced flowering is a unique trait of longan, enabling year‐round production. To investigate its mechanisms, KClO_3_ was applied to ‘Shixia’ trees. Floral induction reached 64% at 41 days after treatment (DAT) and 85% at 54 DAT. In contrast, the control (CK) group showed only 25% at 54 DAT (January 11, 2017) (Figure S39).

RNA‐seq of treated and control buds across 10 time points identified 2851 DEGs, including 193 TFs and 155 hormone‐related genes. Most TFs (e.g., ERF, C2H2, WRKY and MYB) were down‐regulated, while 33 TFs, predominantly from the MIKC‐MADS family, were up‐regulated at later stages (Figure S40). GA pathway genes *DlGA3ox2* and several *DlGASA* genes were likely up‐regulated (Figure S41). Genes associated with oxidative stress and detoxification appeared to be induced. Specifically, 17 flavonoid biosynthesis genes related to antioxidant activity, 12 genes involved in redox reactions and five genes encoding lignin degradation and detoxification enzymes were significantly up‐regulated at the transcription level (Figure S42).

**FIGURE 4:**
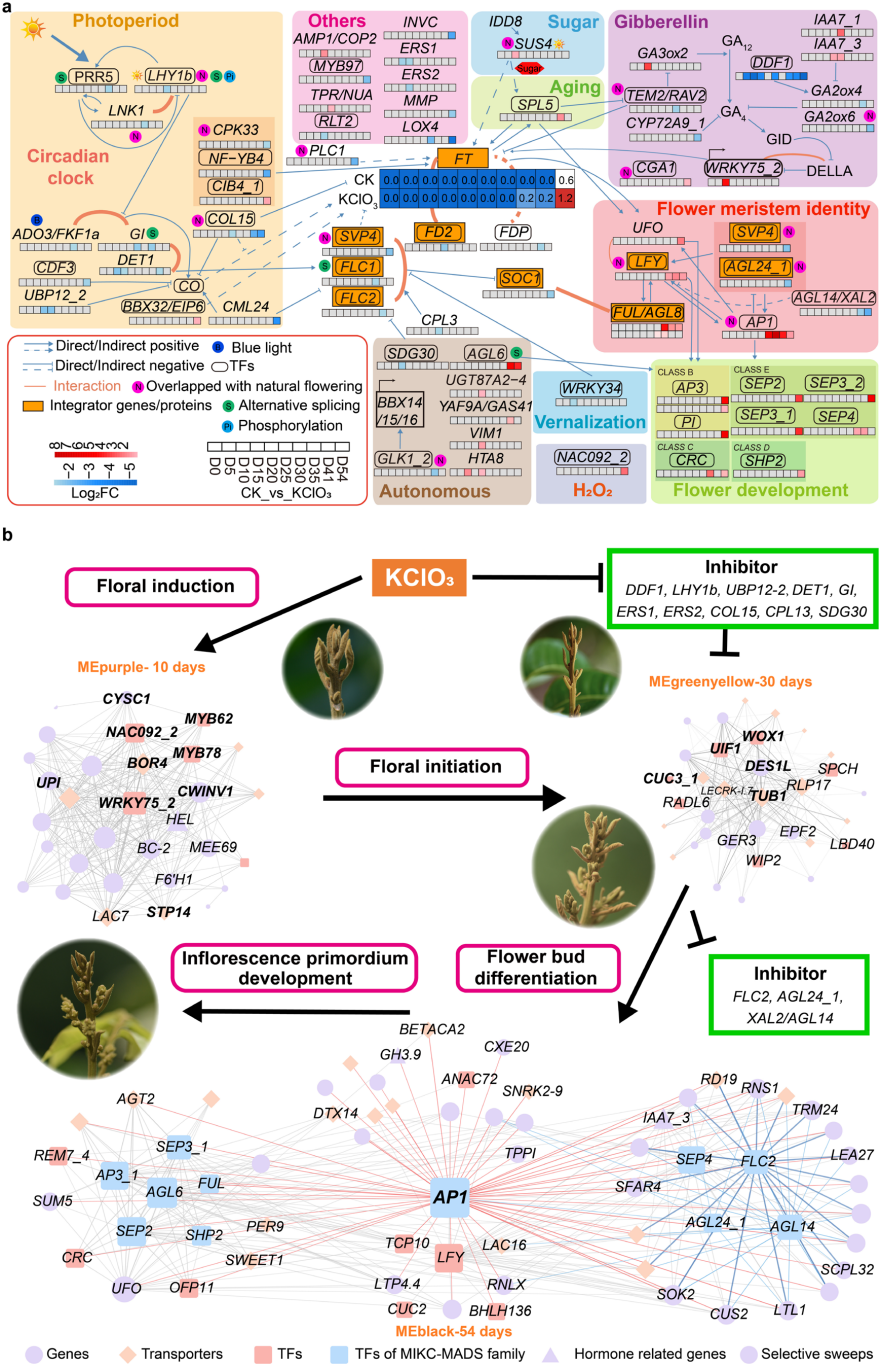
Transcriptome analysis of KClO_3_ induced early flowering in longan. (a) Integrated gene regulatory network (GRN) of floral pathways under KClO_3_‐induced flowering. Differentially expressed flowering genes between KClO_3_ treatment and CK (control) at the same time points were used to construct the network. Heatmaps display log_2_FC values for flowering genes, except for *DlFT1*, which is shown as TPM values across different time points and treatments. Arrows indicate positive regulation, bars represent negative regulation and orange lines denote protein interactions between genes. (b) Hypothesised model for the molecular mechanism of KClO_3_‐induced early flowering in longan. The gene correlation networks are categorised into three stages: Early (purple), middle (greenyellow) and late (black). MEPurple includes 31 genes (weight > 0.1), MEGreenyellow includes 33 genes (weight > 0.1) and MEBlack includes 66 genes (weight > 0.2), representing floral induction, floral initiation and flower bud/inflorescence development, respectively.

GRN analysis of 70 floral pathway DEGs revealed early activation of the GA pathway (Figure 4a). *DlDDF1*, a repressor of GA biosynthesis, was down‐regulated by up to 255‐fold from 5 DAT (Figure S43). While *DlGA3ox2* was up‐regulated 13.5‐fold at 10 DAT. The GA signalling gene *DlWRKY75_2* also appeared to be strongly up‐regulated at 10 DAT. Two *INDOLE‐3‐ACETIC ACID 7* homologues (*DlIAA7s*), which inhibit GA_4_ degradation, were also up‐regulated between 20 and 25 DAT. GA_4_‐deactivating genes *DlGA2ox4* and *DlGA2ox6* were down‐regulated from 41 to 54 DAT. Concurrently, the sugar pathway gene *SUCROSE SYNTHASE 4* (*DlSUS4*) was likely activated at 10 DAT.

Between 10 and 41 DAT, autonomous pathway genes (e.g., *DlHTA8*, *DlUGT87A2_1*, *DlVIM1*, *DlAGL6*) were up‐regulated, while floral repressors *DlSVPs* and *DlFLCs* were repressed (Figure 4a, Figure S44). Although vernalisation‐related genes did not show significant differential expression, early changes may contribute to *DlFLCs*' repression (Figure S45). At later stages, *DlFT1* showed moderate likely up‐regulation, followed by strong induction of floral meristem identity genes, *DlAP1*, *DlFUL* and *DlLFY*, and sequential activation of ABC(D)E floral organ identity genes.

Using WGCNA, 12 co‐expression modules were identified from 2851 DEGs, with MEpurple, MEgreenyellow and MEblack associated with different stages of KClO_3_‐induced flowering (Figure S46). MEpurple, which may link to early floral induction (10 DAT), was enriched in stress and metabolic pathways (Figures S47 and S48). This module contained stress‐related flowering genes *DlWRKY75_2*, *DlNAC092_2* and Fe(II)‐ and 2‐oxoglutarate‐dependent dioxygenase family gene *FERULOYL‐COA6‐HYDROXYLASE1* (DlF6’H1) (Figure 4b). *DlDDF1* was co‐expressed with nine 5 DAT CK hub genes in MEbrown, while *DlCUC3_2*, down‐regulated by KClO_3_ similar to natural flowering, was also included (Figure S49).

MEgreenyellow module, may associate with 30 DAT, was related to floral initiation and meristem determination. Key hub genes included *DlUIF1*, *DlWOX1*, *DlCUC3_1* and floral tissue‐specific *DlLECRK‐I.7*. CTK‐signalling repressors were present, including *DlKMD4* and *DlPUP4* (Figure 4b, Figure S50a).

MEblack, likely related to floral transition and inflorescence primordium development, was enriched in genes involved in floral organ formation and reproductive shoot system development (Figure S50b). Key regulatory genes included *DlAP1* and *DlLFY*. Positively correlated genes included *DlAGL6*, *DlUFO* and ABC(D)E class genes, whereas *DlAGL24_1*, *DlFLC2* and *DlAGL14* were negatively correlated.

### 2.5 Functional Validation of *DlDDF1* in Flowering Regulation

**FIGURE 5:**
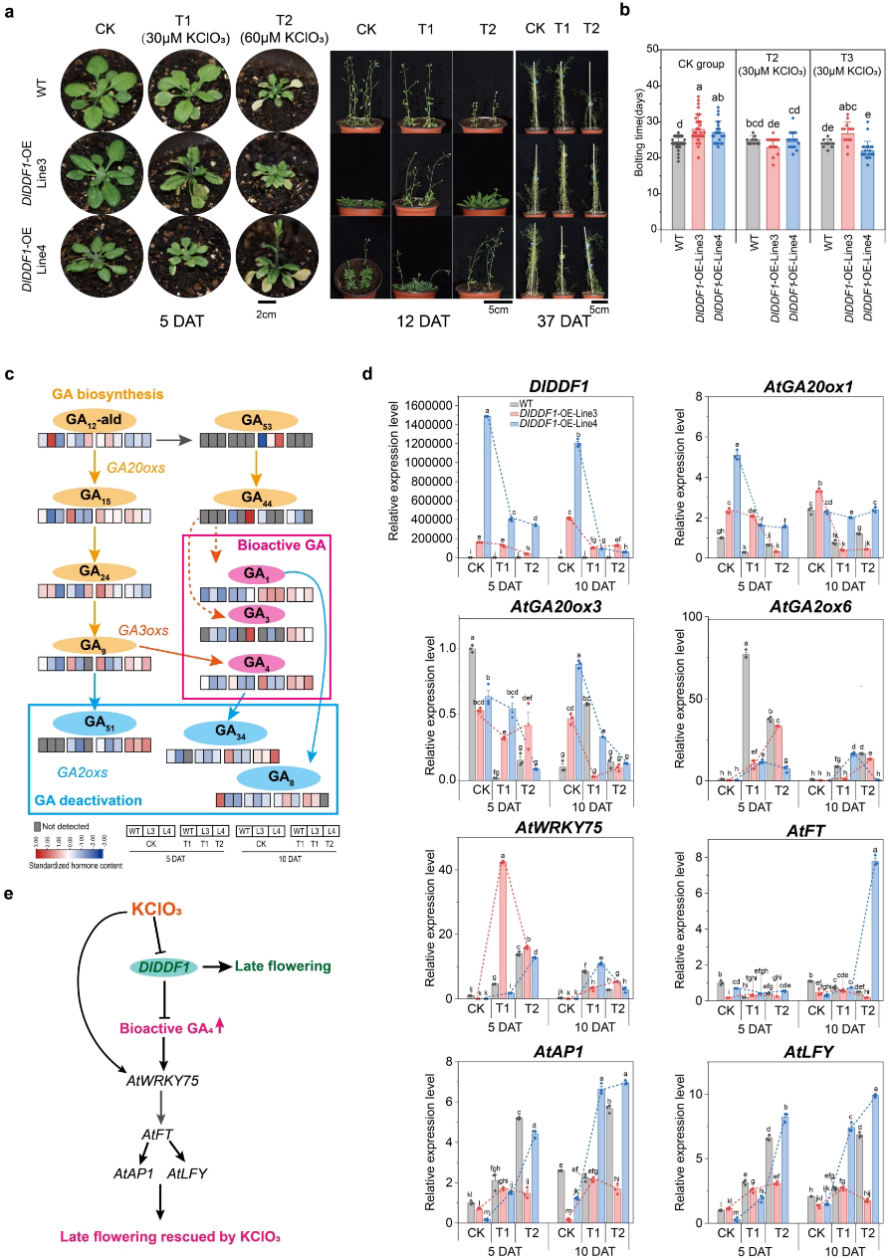
Effects of *DlDDF1* overexpression and KClO_3_ treatment on *Arabidopsis thaliana* . (a) Phenotypes of 16‐day‐old wild‐type (WT) and *DlDDF1*‐overexpression (OE) lines treated with different concentrations of KClO_3_ for 5, 12 and 37 days after treatment (DAT). (b) Bolting time statistics of WT and OE plants under different treatments (*n* ≥ 20). Different letters indicate significant differences (two‐way ANOVA, Tukey's multiple comparisons test, *p* < 0.05). (c) Gibberellin contents in WT and OE plants under different treatments and stages, shown on heatmap (mean of three biological replicates). (d) Relative gene expression levels of flowering‐related genes at 5 DAT and 10 DAT, calculated using the $2^{-∆∆C_{t}}$ method and presented as mean ± SD of three biological replicates (each replicate representing the mean of three technical replicates). (e) Proposed model showing how KClO_3_ treatment rescues the late‐flowering phenotype of *DlDDF1*‐OE plants.

Since *DlDDF1* responded earliest to KClO_3_, it is likely involved in KClO_3_‐induced flowering. To investigate its function, we generated transgenic *Arabidopsis thaliana* plants overexpressing *DlDDF1*. Compared with the wild type (WT), the transgenic plants exhibited a significant delay in flowering, with an average delay of 4 days and a maximum delay of 10 days (*n* ≥ 20, *p* < 0.05). In addition, overexpression lines developed more curled, rod‐like and darker rosette leaves, and produced up to 14 additional rosette leaves relative to WT plants (Figure 5a,b, Figure S53).

For 16‐day‐old plants treated with 30 or 60 μM KClO_3_ (*n* = 60), the delayed flowering phenotype of *DlDDF1*‐overexpressing lines was partially rescued (Figure 5a,b). Specifically, line 3 under 30 μM KClO_3_ and line 4 under both 30 and 60 μM treatments bolted at times comparable to the WT, whereas the 60 μM treatment of line 3 showed no significant change in bolting time (*n* ≥ 20, *p* < 0.05). Notably, 60 μM KClO_3_ markedly inhibited growth in WT plants, resulting in shorter stature and slower development, whereas *DlDDF1*‐overexpressing plants were much less affected (Figure 5a). Endogenous phytohormone profiling indicated that overexpression of *DlDDF1* significantly reduced GA_9_ and bioactive GA_4_ levels; however, this reduction was partially reversed by KClO_3_ treatment (Figure 5c).

Gene expression analysis showed that KClO_3_ treatment significantly reduced *DlDDF1* expression in the overexpression lines, while *AtWRKY75* was likely induced and up‐regulated in both WT and *DlDDF1*‐overexpressing plants after treatment (Figure 5d) (*p* < 0.05). Overexpression of *DlDDF1* repressed the expression of *AtFT*, *AtAP1* and *AtLFY*; however, KClO_3_ treatment caused a pronounced up‐regulation of these flowering‐related genes in the transgenic plants, which may be associated with the observed rescue of the late‐flowering phenotype. In addition, bioactive GA biosynthesis genes *AtGA20ox1*, *AtGA20ox2* and *AtGA20ox3* consistently expressed with *DlDDF1*, while catabolic enzymes *AtGA2ox2*, *AtGA2ox4*, *AtGA2ox6*, *AtGA2ox7* and *AtGA2ox8* exhibited an opposite trend, showing a positive response to KClO_3_ (Figure S54).

## 3 Discussion

KClO_3_ effectively induces year‐round off‐season flowering in longan, providing substantial economic benefits. Our analysis revealed that the GA flowering‐time pathway likely played a central role in KClO_3_‐induced early flowering in spring, with significant gene expression changes observed as early as 5 DAT. These changes appear to include down‐regulation of *DlDDF1*, *DlGA2ox4* and *DlGA2ox6*, and up‐regulation of *DlGA3ox2*, *DlIAA7s*, *DlWRKY75_2* and *DlGASAs*, which may facilitate the conversion of GA_12_ to bioactive GA_4_ (Olszewski et al. 2002; Osnato et al. 2012) (Figure 4a). Identifying the primary pathway triggered by KClO_3_ represents a substantial advancement in understanding the gene network responsible for off‐season flowering in longan. This insight lays the groundwork for future research and applications in flowering regulation and agricultural innovation.

GA_4_ is the most active gibberellin and promotes flowering via *LFY* (Eriksson et al. 2006). Overexpression of *DDF1* in Arabidopsis up‐regulates *AtGA2ox7* and promotes GA degradation and delays flowering (Magome et al. 2008; Yuan et al. 2024). In rice, *OsDDF1* represses floral initiation by negatively regulating *OsAPO2*, an ortholog of *LFY* (Duan et al. 2012). Conversely, *LFY* can be up‐regulated by exogenous GA, leading to early flowering (Blázquez et al. 1997; Yamaguchi et al. 2014). *DlDDF1* was also significantly down‐regulated in the natural flowering of variety ‘Shixia’ (2314‐fold) and three other varieties (Figure S55). *DlDDF1* down‐regulation is a hallmark of both natural and KClO_3_‐induced flowering in longan. KClO_3_ treatment triggered a stronger and faster down‐regulation than natural flowering. Functional characterisation in this study confirmed that *DlDDF1* indeed induces delayed flowering in Arabidopsis, whereas KClO_3_ treatment could rescue the late‐flowering phenotype mainly by suppressing *DlDDF1* expression, increasing GA_4_ content and up‐regulating *AtWRKY75*, *AtFT*, *AtAP1* and *AtLFY* (Figure 5e). In addition, KClO_3_ exerted minimal effects on the flowering time of WT Arabidopsis but markedly promoted flowering in the *DlDDF1*‐overexpressing lines, further supporting the specific responsiveness of *DlDDF1* to KClO_3_.

KClO_3_ is a strong oxidant (Ali et al. 2017), known to induce oxidative stress in longan buds, disrupting photosynthesis and increasing ROS levels (Huang et al. 2006; Yang et al. 2015). Correspondingly, oxidative stress‐responsive genes were up‐regulated, further supporting the occurrence of oxidative stress (Figure S42). Additionally, *DlWRKY75_2* was activated early in response to KClO_3_ (Figure 4b). Its Arabidopsis ortholog promotes *FT* expression by directly binding to its promoter (Zhang et al. 2018). Its activity is repressed by DELLA proteins, which physically interact with *AtWRKY75* and inhibit *FT* activation, placing *AtWRKY75* downstream of GA signalling. Arabidopsis orthologs of KClO_3_‐induced genes *DlNAC092_2* and *DlF6’H1* participate in H_2_O_2_ signalling by mitigating ROS levels and limiting mycotoxin‐induced cell death upon H_2_O_2_ exposure (Balazadeh et al. 2010; Bastow et al. 2004). These findings suggest a potential interplay between oxidative stress and GA pathway‐mediated floral induction.

Natural floral induction in longan depends on chilling, with varieties like ‘Shixia’ requiring temperatures below 18°C (Li 2021). KClO_3_ was applied in November when low temperatures were also present, overlapping with early vernalisation. Compared with natural flowering, KClO_3_ accelerated floral transition and enhanced flowering rate (Figure S39).

Transcriptome analysis revealed that, in addition to GA pathway activation, key floral repressors, *DlFLCs* and *DlSVPs*, associated with vernalisation and autonomous pathways, were down‐regulated earlier under KClO_3_ treatment (Figure S45). Both the autonomous and vernalisation pathways promote flowering by repressing *FLC* (Bastow et al. 2004; Jiang et al. 2009; Streitner et al. 2008; Wang et al. 2012; Yoo et al. 2011; Zhang et al. 2003), which controls flowering time through feedback with FT/FD (Luo et al. 2019). In *Arabidopsis*, *AtFLC* acts as a central floral repressor regulated epigenetically via PRC2‐mediated H3K27me3, non‐coding RNAs (e.g., *COOLAIR*) and DNA methylation in response to photoperiod, vernalisation, autonomous signals and stress‐induced flowering (Shi et al. 2023, 2022; Wu et al. 2020; Yaish et al. 2011). Elevated *AtFLC* expression suppresses floral integrators like *AtFT* and *AtSOC1*, delaying flowering.

Most core vernalisation pathway genes showed no significant expression changes under KClO_3_ treatment compared to natural flowering. However, several *FLC*‐related epigenetic regulators displayed altered timing. For example, *DlPHB3*, *DlVIL3_1* and *DlSKB1* were up‐regulated earlier, while *DlSMZ* and *DlSDG7* were down‐regulated more rapidly (Figure S45). *DlPHB3* also showed elevated expression in floral buds of naturally flowering trees (Figure 3b). These genes are involved in *FLC* repression via histone modification. For instance, *AtSKB1* demethylates histone H4R3 (H4R3sme2) and promotes flowering (Wang et al. 2007). While *AtSDG7* encodes a histone H3K27 methyltransferase, contributing to *AtFLC* repression (Lee et al. 2015).

Autonomous pathway‐related genes, including *DlAGL6*, *DlYAF9A*/*GAS41*, *DlUGT87A2_4*, *DlVIM1* and *DlHTA8*, responded to KClO_3_ treatment. Although some genes were not classified as DEGs, such as *DlGRP7*, they responded rapidly to KClO_3_ treatment (Figure S44). Their Arabidopsis orthologs are known to regulate *FLC* via chromatin remodelling (Crevillén et al. 2019; Zhao et al. 2021).

These results suggest a proposed model in which KClO_3_ may induce flowering by triggering oxidative stress, activating GA signalling and repressing *DlSVPs* and *DlFLCs*. Nevertheless, as this conclusion is based on transcriptome data and preliminary functional validation in Arabidopsis, further studies are required. In particular, functional validation, oxidative stress marker analyses and epigenetic profiling will be necessary to substantiate these mechanisms, especially given that KClO_3_ can induce flowering in non‐chilling seasons. Comprehensive multi‐omics and experimental approaches will therefore be essential to validate this model and to advance our understanding of KClO_3_‐induced flowering, ultimately contributing to improved off‐season flowering in tropical perennials like longan.

Circadian clock genes from the photoperiod pathway, including *DlELF4*, *DlADO3*/*FKF1*, *DlGRP7*, *DlLNK1*, *DlLHY1b*, along with flower meristem identity genes *DlAP1*, *DlLFY*, *DlAGL24_1* and *DlTFL1*, played key roles in natural flowering (Figure 3d). Although these genes showed variable expression under KClO_3_ treatment, only 14 genes shared an expression pattern in both conditions, including the consistent down‐regulation of *DlLHY1b* and *DlLNK1*, suggesting their repression is essential for activating flowering in longan (Figures S51 and S52). In Arabidopsis, *AtELF4* maintains circadian rhythms, while *AtLHY* and *AtCCA1* form a feedback loop that regulates *AtELF4* (Dolzblasz et al. 2016). The *lhy* mutant exhibited early flowering, while overexpression delayed flowering (Moreau et al. 2016), paralleling the observed role of *DlLHY1b* in longan.

In PF of ‘Sijimi’, photoperiod genes *DlCOR28*, *DlCOR27*, *DlADO3*, *DlPRR5* and *DlGI* are centrol regulator (Figure 3e). Arabidopsis orthologs, *AtCOR27*/*28* target core circadian clock genes *AtLHY* and *AtCCA1* to modulating circadian clock and flowering time (Li et al. 2016). Overexpression of *AtCOR27*/*28* s promotes flowering; mutations delay it (Li et al. 2016; Wang et al. 2017). Under long‐day conditions, circadian regulators *AtGI* and *AtADO3* enhance *AtCO* and *AtFT* expression to induce flowering (Song et al. 2014). In longan, flowering integrates photoperiod, vernalisation and circadian cues (Figure 3d). However, elevated *DlCOR27*/*DlCOR28* expression may reduce reliance on photoperiodic and cold, promoting *DlGI* and *DlADO3* expression and enabling flowering.

Invertase (INV) and other SVs like duplications (DUP) and translocations (TRANS) can drive adaptive divergence and reproductive isolation (Feder and Nosil 2009; Lowry and Willis 2010). SVs between homologous chromosomes of longan and lychee resemble those in *Populus* and *Salix* (Hou et al. 2016), *Vigna unguiculata* (Pan et al. 2023), Camelina (Mandáková et al. 2019), Asteraceae (Kong et al. 2023) and *Vernicia montana* (Li et al. 2024). Longan‐specific genes within SV regions are enriched in ROS response, flavone/flavonol biosynthesis and glutathione metabolism. Flavone biosynthesis genes are highly expressed in longan HDRs, contributing to its yellowish‐brown pericarp and stronger antioxidant capacity. Studies report higher antioxidant metabolites, phenolics and vitamin C in longan compared to lychee (Li et al. 2017; Sai‐Ut et al. 2023; Zhu et al. 2019). While the lychee key regulator *LcMYB1* promotes anthocyanin accumulation, its homologue *DlMYB1* shows low expression in longan pericarp (Chen 2021; Lai et al. 2025; Zhao et al. 2012). Pericarp colour differences likely result from differential flavonoid gene expression, supported by a red‐pericarp longan variant and 25 flavonoid‐related genes in HDRs, including *DlMYB1* in an INV region.

Moreover, flavonoids and phenolics are major metabolites in longan flesh, with a higher level in ‘Shixia’ than ‘Chuliang’ (Lai et al. 2021; Shen et al. 2019; Westermann et al. 2024). Under KClO_3_ treatment, 17 flavonoid biosynthesis genes were up‐regulated, suggesting a role in the oxidative stress response. ‘Shixia’ showed higher and more stable flowering rates than ‘Chuliang’ under KClO_3_ treatment, potentially due to stronger antioxidant capacity aiding redox homeostasis and floral transition. These insights are mainly based on transcriptomic and genomic analyses together with preliminary functional validation, and require further investigation into the molecular mechanisms regarding antioxidant activity, flavonoid metabolism, hormone signalling and floral induction.

This study produced a high‐quality longan genome assembly with only three remaining gaps, representing the most complete reference to date. One gap is located in a gene‐rich region, while the other two are in transposable element–rich regions. Although one gap in the genic region has a limited impact at the genome scale, these gaps may introduce local uncertainties in gene annotation and repeat content, as has also been observed in other plant genomes (Gladman et al. 2023; Yue et al. 2023). Future advances in sequencing and scaffolding technologies are expected to close these gaps, further enhancing the application of this genome for functional genomics research and longan improvement.

## 4 Methods

### 4.1 Plant Materials, DNA/RNA Extraction and Library Construction

Genomic DNA of the longan variety *‘Shixia’* was isolated from young leaves collected at the National Fruit Tree Germplasm Longan and Loquat Nursery (Fuzhou, China). DNA extraction was performed using a modified cetyltrimethylammonium bromide (CTAB) method (Clarke 2009), followed by purification with the QIAquick Gel Extraction Kit (QIAGEN). The purified high‐quality DNA was then used for PacBio library construction.

To investigate genes involved in natural floral transition, flower buds and leaf buds were collected from ‘Sijimi’, ‘Shixia’, ‘Dongbao NO. 9’, ‘Biaogui’ and ‘Vietnam Erzao’. To examine KClO_3_‐induced off‐season flowering, 30 ‘Shixia’ trees (14–16 years) were used in a single‐factor randomised experiment with 15 replicates from Maoming, Guangdong, China. On November 18, 2016, KClO_3_ (99% active ingredient) was applied by soil drenching to the treatment group, while the CK group received no treatment but was managed similarly. Bud tissues from five trees were pooled to form a single sample, with three biological replicates per group. Samples were collected on the treatment day and every 5 days for eight samples, with additional collections on Days 41 and 54. Samples were frozen in liquid nitrogen and stored at −80°C until RNA extraction. A paired‐end library was constructed using the Illumina TruSeq RNA Sample Preparation Kit and sequenced on the Illumina X Ten platform.

### 4.2 PacBio High Fidelity Reads (HiFi) Reads Sequencing

Genomic DNA from ‘Shixia’ was fragmented, repaired and ligated to form a DNA library, which was sequenced on the PacBio Sequel‐II third‐generation sequencer to obtain high‐quality sequencing data. Polymerase reads with junctions were converted to circular consensus sequencing (CCS) reads. Quality filtering was applied to retain only CCS reads with a QV ≥ 20, yielding the final valid data for analysis.

### 4.3 Hi‐C Library Construction and Sequencing

Hi‐C libraries were constructed using ~10 g of fresh young leaf tissue from the ‘Shixia’ variety, following BioMarker Technologies' protocol. Leaf samples were fixed in formaldehyde, lysed and digested with Hind III for 12 h. Biotinylated sticky ends were ligated to form chimeric junctions, enriched and fragmented to 500–700 bp. These fragments, representing long‐range interactions, were used to create paired‐end sequencing libraries, yielding approximately 294 million 150 bp paired‐end reads on the Illumina HiSeq X Ten platform.

### 4.4 Genome Assembly

De novo assembly of the ‘Shixia’ longan variety was conducted at the contig level using HiFiasm (v0.19.8‐r603) (Cheng et al. 2021). The assembly used PacBio HiFi reads as input and was performed mostly with default parameters. The key parameters included a k‐mer size of 51 (‐k 51), minimiser window size of 51 (‐w 51), three rounds of error correction (‐r 3), four rounds of assembly graph cleaning (‐a 4) and enabled contig post‐joining (‐u 1). The haploid genome size was automatically estimated (‐‐hg‐size auto). Contigs overlapping > 80% with another were considered redundant and removed, as were those matching * *A. thaliana* * organelle sequences. The final contig‐level assembly included 113 contigs, totalling 442 680 685 bp, with a contig N50 of 28.34 Mb, surpassing the expected haploid genome size and indicating partial heterozygosity resolution.

### 4.5 Hi‐C Scaffolding

Hi‐C data preprocessing began with quality control to remove low‐quality reads and adapter contamination. HiC‐Pro (v.3.1.0) was used to map Hi‐C reads to the 113 contigs assembled by HiFiasm (Cheng et al. 2021), generating a Hi‐C contact matrix to visualise physical interactions between contigs (Servant et al. 2015). Contigs were anchored to chromosomes using EndHiC (v.1.0) with Hi‐C contact data to optimise their order and placement (Wang, Li, et al. 2022; Wang, Wang, et al. 2022). The assembly was visualised with Juicebox (v.2.20.00) for manual refinement. A final quality assessment confirmed accurate contig anchoring and chromosome structure integrity.

### 4.6 Evaluation of Assembly Quality

Genome assembly completeness was assessed using the embryophyta_odb and eudicots_odb databases in BUSCO (Manni et al. 2021) with default parameters. HiFi reads were aligned to the ‘Shixia’ genome using BWA (v.0.7.17), and coverage was calculated with samtools (v.1.19.1). The LTR Assembly Index (LAI) was evaluated using LTR_retriever (v2.9.0) with candidate LTR retrotransposons identified by LTRharvest (v1.6.1) and LTR_FINDER_parallel (v1.1). Key parameters for LTRharvest included LTR length 100–7000 bp, internal distance 1000–15 000 bp, TSD length 4–20 bp, motif TGCA with one mismatch and minimum LTR similarity 85%. LTR_FINDER_parallel was run with 5 Mb genome chunks, maximum runtime 500 s per chunk and parameters matching LTRharvest (LTR length 100–7000 bp, internal distance 1000–15 000 bp, minimum similarity 85%). The combined LTR candidates were input to LTR_retriever for LAI calculation. Multi‐threading and a Singularity container were used to ensure reproducibility.

Assembly accuracy was estimated using 19‐k‐mer consensus quality (QV) with Merqury (v.1.3) and HiFi reads (Rhie et al. 2020). First, 19‐mer counts of the HiFi reads were computed using Meryl with 20 threads and 24 GB memory (*k* = 19, threads = 20, memory = 24G). The resulting k‐mer database was used as input to Merqury to calculate QV and completeness metrics for the genome assembly.

The centromeres and telomeres of the genome were predicted using QuarTeT (Lin et al. 2023). In the longan genome, the telomeric repeat monomer was identified as ‘AAACCCT’, while the centromeres were determined based on regions with the highest region score.

### 4.7 Repeat Annotation

Repetitive elements in the ‘Shixia’ genome were annotated using RepeatMasker (v4.1.2) with a custom repeat library generated by RepeatModeler (v2.0.3), TEsorter (v1.4.6) and DeepTE, relying solely on the de novo repeat library without incorporating Repbase sequences. RepeatModeler (v.2.0.3) was first used to create a de novo repeat library and identify repetitive sequences (Flynn et al. 2020). Unannotated elements were further classified with TEsorter (v1.4.6) (Zhang et al. 2022), and those remaining were classified using DeepTE, applying deep learning techniques (Yan et al. 2020). Annotations from RepeatModeler, TEsorter and DeepTE were integrated and masked with RepeatMasker (v.4.1.2) (www.repeatmasker.org) to highlight repetitive regions. The buildSummary.pl script from RepeatMasker generated detailed statistics on repetitive sequences. LTR retrotransposon (LTR‐RT) insertion times were estimated using *T* = *K*/2*r*, with a base substitution rate of *r* = 1.4 × 10^−8^ (Koch et al. 2000). TE divergence was assessed from identity scores of pairwise TE sequence alignments. Figures illustrating LTR‐RT insertion times and TE divergence were generated with R (v.4.4.1).

### 4.8 Gene Annotation

Gene structures were predicted using the GETA pipeline (https://github.com/chenlianfu/geta), integrating homologous sequences, de novo gene prediction and transcript data. First, RepeatModeler and RepeatMasker were used to annotate and mask repeat sequences in the ‘Shixia’ genome. RNA‐seq data from leaf, bud and aril were processed with Trimmomatic (v.0.39) to remove low‐quality reads (Bolger et al. 2014) and aligned to the reference genome using HISAT2 (v.2.1.1) to identify introns and optimal transcripts. The ‘sam2transfrag’ function converted SAM files to transfrag format for transcriptome analysis, optimising transcript structures. Open reading frames (ORFs) were identified using TransDecoder (v.5.5.0). Homology‐based gene prediction was performed using protein sequences from lychee (Hu et al. 2022), rambutan (Zhang et al. 2021), soapberry (Xue et al. 2022) and longan (‘JDB’) (Wang, Li, et al. 2022, Wang, Wang, et al. 2022). The AUGUSTUS model (v.3.5.0) (Stanke et al. 2006) was trained on 500 protein sequences for de novo prediction. Outputs were validated by alignment with A.hmm from pFAM using HMMER (v.3.1). Genes with coding sequences (CDs) < 150 bp were removed, resulting in a set of high‐confidence, non‐redundant gene models for ‘Shixia’. tRNA annotation was done using tRNAscan‐SE (v.2.0), rRNA with Barrnap (https://github.com/tseemann/barrnap) and miRNA and snRNA using cmscan from INFERNAL against the Rfam database (https://rfam.org/).

### 4.9 Gene Function Annotation

Gene function prediction involved aligning sequences against multiple databases using BlastP with an *E*‐value cutoff of 1e‐5. The NR and Swiss‐Prot databases were hosted locally. Online annotations were performed using eggnog‐mapper (v.2, http://eggnog‐mapper.embl.de/) and Mercator4 (v.6.0, https://www.plabipd.de/mercator_main.html). KEGG pathway assignments were done with BlastKOALA (KEGG BlastKOALA). For floral bud analysis, protein sequences were aligned with the TAIR database (Araport11). Transporters were identified using TCDB (https://www.tcdb.org/), while TFs and protein kinases (PKs) were identified through iTAK (http://itak.feilab.net/cgi‐bin/itak/index.cgi) and PlantTFDB (https://planttfdb.gao‐lab.org/) using local BlastP. Flowering time‐related genes were predicted based on alignments with Arabidopsis and rice genes using the FLOR‐ID ([FLOR‐ID] (Bouché et al. 2016)), PlantCFG ([PlantCFG] (Liu et al. 2024)) and PFGD ([PFGD] (Wu et al. 2024)) databases.

### 4.10 Phylogenetic Analysis, Genomic Comparison and Divergence Time Estimation

Protein sequences of the longest transcripts from 12 Sapindales species ( *Citrus sinensis* , *Pistacia chinensis* , *Pistacia vera* , soapberry, rambutan, longan, lychee, yellowhorn, *Aesculus chinensis*, *Acer yangbiense*, *Dipteronia sinensis*, *Dipteronia dyeriana*) and six outgroup species (*Amborella trichopoda*, *Oryza sativa* , *Vitis vinifera* , apple ( *Malus domestica* ), Arabidopsis, *Carica papaya* ) were used for phylogenetic analysis. Single‐copy orthologs were identified with OrthoFinder (v.2.5.2), and the phylogenetic tree was constructed using ASTRAL‐II (v.5.7.3) (https://github.com/smirarab/ASTRAL). Divergence times were estimated with MCMCtree from the PAML package (Yang 2007) and calibrated using rice divergence data from Timetree (https://timetree.org/). The tree was visualised with FIGTREE (v.1.4.4).

### 4.11 Genomic Comparison and Karyotype Evolution of Longan Genome

Synteny searches compared the longan genome structure with that of lychee (Hu et al. 2022), soapberry (Xue et al. 2022), rambutan (Zhang et al. 2021) and yellowhorn (Liang et al. 2019) genomes. Collinearity was analysed using JCVI with a minimum of 30 synthetic genes (Tang et al. 2024). To model karyotype evolution, we referred to the ancestral chromosome composition of closely related genera based on their collinearity with yellowhorn. Chromosome SVs between longan and lychee were identified using SYRI (v.1.7.0) (https://github.com/schneebergerlab/syri) (Goel et al. 2019).

### 4.12 Gene Family Analysis

Gene family clustering analysis was conducted for longan and lychee based on the longest transcript protein sequences. OrthoFinder (v.2.5.2) clustered genes using default parameters (‘‐S diamond, ‐M msa’). For longan‐specific genes compared to lychee, pairwise protein alignments were performed with BLAST (v.2.2.26; blastp) and *E*‐values < 1 × 10^−5^. CAFE5 (Mendes et al. 2020) analysed gene family changes across 18 species based on ortho‐group gene counts, with *λ* = 0.0034 and a final likelihood of 397 593. NBS gene families were analysed across four genera and three longan cultivars. Conserved domains were sourced from the pFAM database (http://pfam.xfam.org/) and orthologs were identified using HMMER (v.3.4) with an *e*‐value ≤ 0.01. ClustalW (v.2.1) performed multiple sequence alignment, followed by HMMER searches for species‐specific gene family models. Family members were confirmed with BlastP (*e*‐value ≤ 1 × 10^−5^) and domain scans from the InterPro database (https://www.ebi.ac.uk/interpro/search/sequence/).

### 4.13 RNA‐Seq Analysis

Raw reads were trimmed using fastp v0.19.4 (https://github.com/OpenGene/fastp) to remove Illumina adapters and low‐quality bases with default parameters. Trimmed reads were aligned to the ‘Shixia’ genome using HISAT2 (v.2.1.0, https://github.com/infphilo/hisat2). Gene expression was quantified by calculating reads per gene and normalising to Transcripts Per Kilobase Million (TPM) using Stringtie (v.2.1.2) (Kovaka et al. 2019) with default settings. DEGs were identified using the R package DESeq2 (Love et al. 2014) and limma (Ritchie et al. 2015), with thresholds of |log_2_(FC)| > 1 and a false discovery rate (*FDR*) < 0.05 calculated using the Benjamini–Hochberg correction for multiple testing.

### 4.14 Gene Regulatory Networks for Flowering Genes

The GRN in Figure 3d for natural flowering was constructed using DEGs between flower buds and leaf buds across five longan varieties. Genes consistently differentially expressed were selected, including those involved in the flowering pathway, hormone signalling, meristem identity and heat shock response. The GRN in Figure 4a, representing KClO_3_ induced flowering was based on flowering‐related DEGs between treatment and control groups. Regulatory interactions for both networks were derived primarily from the FLOR‐ID database (http://www.phytosystems.ulg.ac.be/florid/).

### 4.15 Gene Co‐Expression Networks Construction and Analysis

Gene co‐expression networks were constructed using the R package WGCNA (Langfelder and Horvath 2008) and Cytoscape (v3.10.1) for network visualisation. Prior to network construction, genes with low expression (mean TPM < 1 across all samples) were removed to reduce noise. We designed multiple co‐expression analyses based on different biological contexts:

#### 4.15.1 Natural Flowering Condition Across Five Varieties

DEGs of flower and leaf buds of each variety that consistently up‐ or down‐regulated across all varieties were subjected to WGCNA. Given their biological significance and the strong association of all identified modules with tissues, as well as the high correlation among module eigengenes (*r* > 0.8), all genes from the relevant modules were combined to construct a unified co‐expression network for further analysis. To retain strongly co‐expressed genes, a stringent co‐expression threshold (> 0.87, top 10%) was applied to the up‐regulated gene set, whereas a lower threshold (> 0.45, top 10%) was used for the down‐regulated set due to generally weaker co‐expression levels.

#### 4.15.2 ‘Sijimi’‐Specific Flowering Network

After filtering low expression genes, transcriptome data of 19,104 genes from flower and leaf buds of five varieties were analysed with WGCNA (*minModuleSize* = 100, *reassignThreshold* = 0.05, *mergeCutHeight* = 0.3). The MEpink module (692 genes) showed the strongest correlation with ‘Sijimi’ floral buds. To retain only the strongest co‐expression relationships, a co‐expression weight threshold of > 0.26 (top 0.15%) was applied to extract 47 highly co‐expressed genes.

#### 4.15.3 Shared Flowering Genes Across Varieties

A curated list of 20 up‐regulated and 16 down‐regulated flowering‐related genes was compiled. Pearson correlation coefficients were calculated separately for each set, and gene pairs with *r* > 0.7 were retained for network construction.

#### 4.15.4 KClO_3_‐Induced Off‐Season Flowering

2581 DEGs of bud tissues from KClO_3_‐treated and control groups were subjected to WGCNA (*minModuleSize* = 20, *reassignThreshold* = 2, *mergeCutHeight* = 0.3), resulting in 12 modules. Four modules that were highly correlated with key time points in either treatment or control groups were extracted for further analysis (Figure S46). Given the relatively smaller module sizes, the following weight thresholds were applied for network visualisation: MEpurple > 0.1, MEgreenyellow > 0.1, MEblack > 0.2 and MEbrown > 0.35. These thresholds were selected to construct networks composed of the most strongly co‐expressed gene pairs, while also ensuring the inclusion of key flowering‐related DEGs, the stability of hub genes and the interpretability of the overall network structure.

### 4.16 Functional Enrichment Analysis

The amino acid sequence of the genome was aligned with the GO and KEGG databases to create a genome background file. Functional enrichment analysis was conducted using R (v.4.3.1) and visualised with OmicShare tools (Mu et al. 2024) and ClueGo plugin in Cytoscape (v.3.10.1) (Bindea et al. 2009).

### 4.17 Generation of *35S::DlDDF1* Transgenic Arabidopsis and Grow Conditions

The full‐length coding sequence (CDS) of *DlDDF1* was cloned from floral tissues of ‘Shixia’ and inserted into a binary vector driven by the CaMV 35S promoter. The construct was amplified in *Escherichia coli* DH5α, transferred into *Agrobacterium tumefaciens* GV3101 and introduced into *A. thaliana* Col‐0 via the floral‐dip method. Transformed plants were grown under long‐day conditions (16 h light/8 h dark, 21°C, 60% relative humidity). Positive T₁ lines were selected based on hygromycin resistance and confirmed by PCR. From the T_2_ generation, the lines exhibiting the strongest late‐flowering phenotype (Line 3 and Line 4) were selected, and T_3_ homozygous lines were used for subsequent experiments.

### 4.18 KClO_3_ Treatment and Sample Collection

To determine a suitable concentration, we first tested different levels of KClO_3_ on WT Arabidopsis and found that 100 μM was a safe, non‐lethal concentration at the flowering stage. For experiments, 16‐day‐old seedlings (four per pot, 60 plants per group in total) were irrigated with 50 mL of water (CK), 30 μM KClO_3_ (T1), or 60 μM KClO_3_ (T2). WT and T_3_ transgenic lines were treated in parallel, and samples were collected at 5 and 10 DAT.

More than 20 plants per group were used for bolting time measurements, with rosette leaf numbers recorded for the same plants. Nine plants, grouped into three biological replicates, were used for RNA extraction and qPCR analysis. Another 30 plants (5 per replicate) were harvested at 5 DAT and 10 DAT for gibberellin quantification.

Statistical analyses were performed using two‐way variance (ANOVA; genotype × treatment) for bolting time and rosette leaf number, and three‐way ANOVA (genotype × treatment × stage) for relative expression levels. Tukey's honestly significant difference (HSD) test was applied for multiple comparisons, and differences were considered statistically significant at *p* < 0.05.

### 4.19 Quantitative Real‐Time PCR

Total RNA was extracted from whole plant tissues using the FastPure Plant Total RNA Isolation Kit (RC401‐01, vazyme, China). First‐strand cDNA was synthesised from RNA using the HiScript III 1st Strand cDNA Synthesis Kit (R213‐01, vazyme, China). Quantitative real‐time PCR (qRT‐PCR) was performed on a Bio‐Rad CFX96 real‐time system (Bio‐Rad, USA) using gene‐specific primers (Table S25) designed with Primer5. The expression of endogenous genes potentially affected by *DlDDF1* overexpression was assessed. Relative expression levels were calculated using the $2^{-∆∆C_{t}}$ method and were presented as mean ± SD of three biological replicates, each consisting of the mean of three technical replicates.

### 4.20 Determination of Gibberellin Content

Approximately 0.5 g of fresh tissue was ground in liquid nitrogen and extracted with 80% methanol containing 1 mM BHT at 4°C overnight. The extract was centrifuged, and the supernatant was evaporated to dryness under vacuum. The residue was re‐dissolved in 50% methanol, filtered through a 0.22 μm membrane and subjected to HPLC‐MS analysis. Separation was performed on a C18 reverse‐phase column using a gradient elution of water (0.1% formic acid) and acetonitrile (0.1% formic acid) at a flow rate of 0.3 mL/min. Gibberellins were detected and quantified using a triple quadrupole mass spectrometer in multiple reaction monitoring (MRM) mode based on standard retention times and mass transitions.

### 4.21 Data Analysis and Visualisation

All statistical analyses were conducted using R (v4.4.02) and Stata (v18.0). Data preprocessing, normalisation and statistical testing were performed as appropriate for each dataset. Graphical visualisations were generated using R (ggplot2 and related packages), GraphPad Prism (v10), OriginPro (v2024) and Adobe Illustrator (AI, v25.4.1) for figure assembly and refinement.

## Author Contributions

R.M., S.Z. and J.L. conceived this genome project, coordinated research activities and designed experiments; S.Z., F.J., J.Z., W.H., X.C. and J.L. collected the plant materials; S.T., G.Z., Q.Z. and B.W. assembled and annotated the genome; R.M., S.Z., J.L. and J.Z. designed RNA‐seq experiments; G.Z., J.M., F.J., B.W. and J.Z. analysed the genome, gene expression and gene network; G.Z. conducted the transgenic plant experiments, KClO_3_ treatments and associated follow‐up analyses; G.Z., W.W., J.Z. and R.M. wrote the manuscript.

## Conflicts of Interest

The authors declare no conflicts of interest.

## Supporting information


**Figure S1:** Workflow of high‐quality chromosome‐level genome assembly for longan ( *Dimocarpus longan* ) cultivar ‘Shixia’.
**Figure S2:** Genome‐wide Hi‐C chromatin interaction map of longan cultivar ‘Shixia’.
**Figure S3:** Telomeres (a) and centromeres (b) predictions in the longan genome.
**Figure S4:** Dot plot of 15 longan chromosomes showing syntenic regions.
**Figure S5:** Collinearity analysis of longan and four closely related species.
**Figure S6:** Dot plot of longan and lychee ( *Litchi chinensis* ) showing collinearity.
**Figure S7:** Dot plot of longan and rambutan ( *Nephelium lappaceum* ).
**Figure S8:** Dot plot of longan and soapberry ( *Sapindus mukorossi* ) showing collinearity.
**Figure S9:** Dot plot of longan and yellowhorn ( *Xanthoceras sorbifolia* ) showing collinearity.
**Figure S10:** Dot plot of longan and five distant species showing low collinearity.
**Figure S11:** Non‐repetitive and repetitive sequence length in the genomes of five closely related species.
**Figure S12:** Density plots of TE insertion times for Copia and Gypsy types in longan, lychee, rambutan, soapberry and yellowhorn.
**Figure S13:** Statistics of transposable element (TE) divergence in longan, lychee, rambutan, soapberry and yellowhorn based on sequence identity.
**Figure S14:** GO and KEGG enrichment analysis of longan‐specific genes identified by BlastP using longan as the query and lychee as the database.
**Figure S15:** GO and KEGG enrichment analysis of longan‐specific genes identified by BlastP using longan as the database and lychee as the query.
**Figure S16:** GO and KEGG enrichment analysis of longan‐specific genes compared to lychee, identified by ortho groups.
**Figure S17:** Phylogenetic tree and gene family expansion/contraction analysis in the evolutionary process of longan.
**Figure S18:** Chromosomal distribution of NBS‐encoding genes in longan and related genera.
**Figure S19:** GO and KEGG enrichment analysis of significantly expanded genes in longan.
**Figure S20:** Expression analysis of flavonoid biosynthesis‐related genes in natural and potassium chlorate‐induced flower buds.
**Figure S21:** Gene function enrichment of longan genes in inversion (INV) regions and structural variation analysis with lychee.
**Figure S22:** Gene function enrichment of longan genes in duplication (DUP) regions of longan and structure variations analysis with lychee.
**Figure S23:** Gene function enrichment of longan genes in translocation (TRANS) regions and structural variation analysis with lychee.
**Figure S24:** Expression comparison of longan genes in HDR and other regions of structural variation between longan and lychee.
**Figure S25:** Abundance of transposable elements (TEs) in inverted regions of the longan genome.
**Figure S26:** Abundant transposable element (TE) counts in chromosomal structural variation regions of longan and lychee.
**Figure S27:** Distribution of connection weights among co‐expressed gene pairs derived from overlapping up‐regulated genes in five longan cultivars under natural flowering conditions.
**Figure S28:** Co‐expression network of overlapping up‐regulated genes in five cultivars under natural flowering conditions.
**Figure S29:** Distribution of connection weights among co‐expressed gene pairs derived from overlapping down‐regulated genes in five longan cultivars under natural flowering conditions.
**Figure S30:** Co‐expression network of overlapping down‐regulated genes in five cultivars under natural flowering conditions.
**Figure S31:** GO enrichment analysis of overlapped up‐regulated genes in flower bud tissues of five cultivars.
**Figure S32:** GO enrichment analysis of uniquely down‐regulated genes in flower bud tissues.
**Figure S33:** GO enrichment analysis of overlapped down‐regulated genes in flower bud tissues of five cultivars.
**Figure S34:** Co‐expression networks of overlapping up‐ and down‐regulated flowering genes in flower bud tissues of five cultivars.
**Figure S35:** Up‐regulated transcription factors (TFs) and hormone‐related genes in the flower bud tissues of all five cultivars.
**Figure S36:** Down‐regulated transcription factors (TFs) and hormone‐related genes in the flower bud tissues of all five cultivars.
**Figure S37:** Specifically expressed DEGs in the flower bud of ‘Sijimi’ compared with other five cultivars.
**Figure S38:** Distribution of connection weights among co‐expressed gene pairs in the ‘Sijimi’ flower bud‐specific MEpink.
**Figure S39:** Comparison of flowering rates between KClO_3_ treatment and control (CK) group.
**Figure S40:** Heat maps of differentially expressed TFs for CK and KClO_3_ treatment at different stages.
**Figure S41:** Heat maps of differently expressed hormone‐related genes for CK and KClO_3_ treatments at different stages.
**Figure S42:** Heat maps of DEGs related to oxidative stress in KClO_3_‐induced flower bud tissues.
**Figure S43:** Expression patterns of gibberellin pathway‐related genes under KClO_3_ treatment and natural flowering conditions (control group).
**Figure S44:** Expression patterns of autonomous pathway‐related genes under KClO_3_ treatment and natural flowering conditions (control group).
**Figure S45:** Expression patterns of vernalisation pathway‐related genes under KClO_3_ treatment and natural flowering conditions (control group).
**Figure S46:** Module‐trait relationships showing the correlation between gene expression levels and trait intensity.
**Figure S47:** Gene function enrichment of gene modules early responded to KClO_3_ treatment.
**Figure 48**. Distribution of connection weights among co‐expressed gene pairs in KClO_3_‐induced flowering‐specific modules.
**Figure S49:** Gene co‐expression network of MEbrown.
**Figure S50:** Gene function enrichment of gene modules involved in floral initiation and flower bud differentiation under KClO_3_ treatment.
**Figure S51:** Venn diagram of flowering time‐related up‐regulated DEGs for KClO_3_‐induced off‐season flowering, natural flowering and perpetual flowering.
**Figure S52:** Venn diagram of flowering time‐related down‐regulated DEGs for KClO_3_‐induced off‐season flowering, natural flowering and perpetual flowering.
**Figure S53:** Rosette leaf analysis of *DlDDF1* overexpression plants.
**Figure S54:** Relative expression levels of genes in different genotypes under KClO_3_ treatments determined by qRT‐PCR.
**Figure S55:** Expression pattern of *DlDDF1* in natural and KClO_3_‐induced flowering.
**Table S1:** Sequencing data for genome assembly of *D. longan* cultivar ‘Shixia’.
**Table S2:** Statistics of the genome assembly for *D. longan* cultivar ‘Shixia’.
**Table S3:** Statistics of contig‐level assembly.
**Table S4:** Statistics for Hi‐C sequencing and mapping analysis.
**Table S5:** Statistics of Hi‐C assembly analysis.
**Table S6:** BUSCO assessment of the ‘Shixia’ genome assembly.
**Table S7:** Consensus quality (QV) and completeness of the ‘Shixia’ genome based on Merqury analysis.
**Table S8:** Merqury quality values of the 15 chromosomes based on HiFi reads.
**Table S9:** Genome consistency assessment.
**Table S10:** BUSCO assessment of protein‐coding genome annotation.
**Table S11:** Genome assembly of ‘Shixia’ longan and comparison with other cultivars and related genera.
**Table S12:** Summary statistics of telomere prediction results of longan genome.
**Table S13:** Statistical summary of the best predicted centromere candidates of longan genome.
**Table S14:** Information on three remaining gaps of the longan genome.
**Table S15:** Summary of genome annotation for longan cultivar ‘Shixia’.
**Table S16:** Gene function annotation statistics.
**Table S17:** Summary of transposable elements (TEs) and other repeats in longan cultivar ‘Shixia’.
**Table S18:** Chromosome size statistics of five closely related species.
**Table S19:** Identification of longan‐specific genes compared with lychee.
**Table S20:** Statistics of NBS‐encoding genes in longan and other four species of closely related genera of Sapindaceae family.
**Table S21:** Distribution of NBS‐encoding genes on the 15 pseudo‐chromosomes of longan.
**Table S22:** Statistics of the chromosome structural variation types in longan compared with lychee.
**Table S23:** Statistics of longan chromosome structural variations in longan and lychee.
**Table S24:** Statistics of transposable element numbers in INV, TRANS and DUP regions of the longan genome.
**Table S25:**. List of qRT‐PCR primer sequences.

## Data Availability

The genome assembly of ‘Shixia’ has been deposited in the China National GeneBank DataBase (CNGBdb) under accession number CNP0006891 (https://db.cngb.org/). The raw sequence data reported in this paper have been submitted to the Genome Sequence Archive (GSA) at the National Genomics Data Center under accession number CRA019839 (https://ngdc.cncb.ac.cn/gsa), which are publicly accessible.

## References

[pbi70433-bib-0001] Ali, S. N. , M. K. Ahmad , and R. Mahmood . 2017. “Sodium Chlorate, a Herbicide and Major Water Disinfectant Byproduct, Generates Reactive Oxygen Species and Induces Oxidative Damage in Human Erythrocytes.” Environmental Science and Pollution Research 24: 1898–1909. 27797001 10.1007/s11356-016-7980-7

[pbi70433-bib-0002] Balazadeh, S. , A. Wu , and B. Mueller‐Roeber . 2010. “Salt‐Triggered Expression of the ANAC092‐Dependent Senescence Regulon in *Arabidopsis thaliana* .” Plant Signaling & Behavior 5: 733–735. 20404534 10.4161/psb.5.6.11694 PMC3001574

[pbi70433-bib-0003] Bastow, R. , J. S. Mylne , C. Lister , Z. Lippman , R. A. Martienssen , and C. Dean . 2004. “Vernalization Requires Epigenetic Silencing of FLC by Histone Methylation.” Nature 427: 164–167. 14712277 10.1038/nature02269

[pbi70433-bib-0004] Bindea, G. , B. Mlecnik , H. Hackl , et al. 2009. “ClueGO: A Cytoscape Plug‐In to Decipher Functionally Grouped Gene Ontology and Pathway Annotation Networks.” Bioinformatics 25: 1091–1093. 19237447 10.1093/bioinformatics/btp101 PMC2666812

[pbi70433-bib-0005] Blázquez, M. A. , L. N. Soowal , I. Lee , and D. Weigel . 1997. “LEAFY Expression and Flower Initiation in Arabidopsis.” Development (Cambridge, England) 124: 3835–3844. 9367439 10.1242/dev.124.19.3835

[pbi70433-bib-0006] Bolger, A. M. , M. Lohse , and B. Usadel . 2014. “Trimmomatic: A Flexible Trimmer for Illumina Sequence Data.” Bioinformatics 30: 2114–2120. 24695404 10.1093/bioinformatics/btu170 PMC4103590

[pbi70433-bib-0007] Bouché, F. , G. Lobet , P. Tocquin , and C. Périlleux . 2016. “FLOR‐ID: An Interactive Database of Flowering‐Time Gene Networks in *Arabidopsis thaliana* .” Nucleic Acids Research 44: D1167–D1171. 26476447 10.1093/nar/gkv1054 PMC4702789

[pbi70433-bib-0008] Buerki, S. , M. W. Callmander , P. Acevedo‐Rodriguez , et al. 2021. “An Updated Infra‐Familial Classification of Sapindaceae Based on Targeted Enrichment Data.” American Journal of Botany 108: 1234–1251. 34219219 10.1002/ajb2.1693 PMC8361682

[pbi70433-bib-0009] Chen, Q. 2021. “Regulatory Mechanism of DlMYB1 on Anthocyanin Biosynthesis in Longan Pericarp”.

[pbi70433-bib-0010] Chen, Z. , X. Lu , L. Zhu , et al. 2023. “Chromosomal‐Level Genome and Multi‐Omics Dataset Provides New Insights Into Leaf Pigmentation in *Acer palmatum* .” International Journal of Biological Macromolecules 227: 93–104. 36470439 10.1016/j.ijbiomac.2022.11.303

[pbi70433-bib-0011] Cheng, H. , G. T. Concepcion , X. Feng , H. Zhang , and H. Li . 2021. “Haplotype‐Resolved De Novo Assembly Using Phased Assembly Graphs With Hifiasm.” Nature Methods 18: 170–175. 33526886 10.1038/s41592-020-01056-5 PMC7961889

[pbi70433-bib-0012] Clarke, J. D. 2009. “Cetyltrimethyl Ammonium Bromide (CTAB) DNA Miniprep for Plant DNA Isolation.” Cold Spring Harbor Protocols 2009: pdb‐prot5177. 10.1101/pdb.prot5177 20147112

[pbi70433-bib-0013] Crevillén, P. , Á. Gómez‐Zambrano , J. A. López , J. Vázquez , M. Piñeiro , and J. A. Jarillo . 2019. “Arabidopsis YAF9 Histone Readers Modulate Flowering Time Through NuA4‐Complex‐Dependent H4 and H2A.Z Histone Acetylation at FLC Chromatin.” New Phytologist 222: 1893–1908. 30742710 10.1111/nph.15737

[pbi70433-bib-0014] Dolzblasz, A. , J. Nardmann , E. Clerici , et al. 2016. “Stem Cell Regulation by Arabidopsis WOX Genes.” Molecular Plant 9: 1028–1039. 27109605 10.1016/j.molp.2016.04.007

[pbi70433-bib-0015] Duan, Y. , S. Li , Z. Chen , et al. 2012. “Dwarf and Deformed Flower 1, Encoding an F‐Box Protein, Is Critical for Vegetative and Floral Development in Rice (*Oryza sativa* L.).” Plant Journal 72: 829–842. 10.1111/j.1365-313X.2012.05126.x 22897567

[pbi70433-bib-0016] Eriksson, S. , H. Böhlenius , T. Moritz , and O. Nilsson . 2006. “GA4 Is the Active Gibberellin in the Regulation of LEAFY Transcription and Arabidopsis Floral Initiation.” Plant Cell 18: 2172–2181. 16920780 10.1105/tpc.106.042317 PMC1560906

[pbi70433-bib-0017] Feder, J. L. , and P. Nosil . 2009. “Chromosomal Inversions and Species Differences: When Are Genes Affecting Adaptive Divergence and Reproductive Isolation Expected to Reside Within Inversions?” Evolution 63: 3061–3075. 19656182 10.1111/j.1558-5646.2009.00786.x

[pbi70433-bib-0018] Flynn, J. M. , R. Hubley , C. Goubert , et al. 2020. “RepeatModeler2 for Automated Genomic Discovery of Transposable Element Families.” Proceedings of the National Academy of Sciences of the United States of America 117: 9451–9457. 32300014 10.1073/pnas.1921046117 PMC7196820

[pbi70433-bib-0019] Gladman, N. , S. Goodwin , K. Chougule , W. Richard McCombie , and D. Ware . 2023. “Era of Gapless Plant Genomes: Innovations in Sequencing and Mapping Technologies Revolutionize Genomics and Breeding.” Current Opinion in Biotechnology 79: 102886. 36640454 10.1016/j.copbio.2022.102886 PMC9899316

[pbi70433-bib-0020] Goel, M. , H. Sun , W.‐B. Jiao , and K. Schneeberger . 2019. “SyRI: Finding Genomic Rearrangements and Local Sequence Differences From Whole‐Genome Assemblies.” Genome Biology 20: 277. 31842948 10.1186/s13059-019-1911-0 PMC6913012

[pbi70433-bib-0021] Hau, T. V. , and T. S Hieu . 2019. “Induction of Flowering in Longan in the Mekong Delta, Vietnam.” Acta Horticulturae 1293: 193–202.

[pbi70433-bib-0022] Hou, J. , N. Ye , Z. Dong , M. Lu , L. Li , and T. Yin . 2016. “Major Chromosomal Rearrangements Distinguish Willow and Poplar After the Ancestral “Salicoid” Genome Duplication.” Genome Biology and Evolution 8: 1868–1875. 27352946 10.1093/gbe/evw127 PMC4943198

[pbi70433-bib-0023] Hu, G. , J. Feng , X. Xiang , et al. 2022. “Two Divergent Haplotypes From a Highly Heterozygous Lychee Genome Suggest Independent Domestication Events for Early and Late‐Maturing Cultivars.” Nature Genetics 54: 73–83. 34980919 10.1038/s41588-021-00971-3 PMC8755541

[pbi70433-bib-0024] Huang, X. M. , J. M. Lu , H. C. Wang , et al. 2006. “Nitrate Reduces the Detrimental Effect of Potassium Chlorate on Longan (*Dimocarpus longan* Lour.) Trees.” Scientia Horticulturae 108: 151–156.

[pbi70433-bib-0025] Jiang, D. , X. Gu , and Y. He . 2009. “Establishment of the Winter‐Annual Growth Habit via Frigda‐Mediated Histone Methylation at Flowering Locus C in Arabidopsis.” Plant Cell 21: 1733–1746. 19567704 10.1105/tpc.109.067967 PMC2714927

[pbi70433-bib-0026] Jue, D. , X. Sang , L. Liu , et al. 2019. “Comprehensive Analysis of the Longan Transcriptome Reveals Distinct Regulatory Programs During the Floral Transition.” BMC Genomics 20: 1–18. 30744552 10.1186/s12864-019-5461-3 PMC6371577

[pbi70433-bib-0027] Koch, M. A. , B. Haubold , and T. Mitchell‐Olds . 2000. “Comparative Evolutionary Analysis of Chalcone Synthase and Alcohol Dehydrogenase Loci in Arabidopsis, Arabis, and Related Genera (Brassicaceae).” Molecular Biology and Evolution 17: 1483–1498. 11018155 10.1093/oxfordjournals.molbev.a026248

[pbi70433-bib-0028] Kong, X. , Y. Zhang , Z. Wang , et al. 2023. “Two‐Step Model of Paleohexaploidy, Ancestral Genome Reshuffling and Plasticity of Heat Shock Response in Asteraceae.” Horticulture Research 10: 1–14. 10.1093/hr/uhad073 PMC10251138 37303613

[pbi70433-bib-0029] Kovaka, S. , A. V. Zimin , G. M. Pertea , R. Razaghi , S. L. Salzberg , and M. Pertea . 2019. “Transcriptome Assembly From Long‐Read RNA‐Seq Alignments With StringTie2.” Genome Biology 20: 1–13. 31842956 10.1186/s13059-019-1910-1 PMC6912988

[pbi70433-bib-0030] Lai, C. , X. Zhang , Y. Guo , et al. 2025. “Identification and Verification of Key Metabolites in Polyamine‐Regulated Flavonoid Production in Longan Embryogenic Calli Using Widely Targeted Metabolomics.” Industrial Crops and Products 231: 121191.

[pbi70433-bib-0031] Lai, T. , L. Shuai , D. Han , et al. 2021. “Comparative Metabolomics Reveals Differences in Primary and Secondary Metabolites Between “Shixia” and “Chuliang” Longan ( *Dimocarpus longan* Lour.) Pulp.” Food Science & Nutrition 9: 5785–5799. 34646546 10.1002/fsn3.2552 PMC8498058

[pbi70433-bib-0032] Langfelder, P. , and S. Horvath . 2008. “WGCNA: An R Package for Weighted Correlation Network Analysis.” BMC Bioinformatics 9: 559. 19114008 10.1186/1471-2105-9-559 PMC2631488

[pbi70433-bib-0033] Lee, J. , J. Y. Yun , W. Zhao , W. H. Shen , and R. M. Amasino . 2015. “A Methyltransferase Required for Proper Timing of the Vernalization Response in Arabidopsis.” Proceedings of the National Academy of Sciences of the United States of America 112: 2269–2274. 25605879 10.1073/pnas.1423585112 PMC4343098

[pbi70433-bib-0034] Li, H. 2021. “Longan Fruit Tree Physiology and Its Flowering Induction.” In Handbook of Plant and Crop Physiology, 4th ed., 77–97. CRC Press.

[pbi70433-bib-0035] Li, P. , X. Yu , and B. Xu . 2017. “Effects of UV‐C Light Exposure and Refrigeration on Phenolic and Antioxidant Profiles of Subtropical Fruits (Litchi, Longan, and Rambutan) in Different Fruit Forms.” Journal of Food Quality 2017: 1–12.

[pbi70433-bib-0036] Li, W. , X. Dong , X. Zhang , et al. 2024. “Genome Assembly and Resequencing Shed Light on Evolution, Population Selection, and Sex Identification in *Vernicia montana* .” Horticultural Research 11: 1–15. 10.1093/hr/uhae141 PMC11233859 38988615

[pbi70433-bib-0037] Li, X. , D. Ma , S. X. Lu , et al. 2016. “Blue Light‐ and Low Temperature‐Regulated COR27 and COR28 Play Roles in the Arabidopsis Circadian Clock.” Plant Cell 28: 2755–2769. 27837007 10.1105/tpc.16.00354 PMC5155342

[pbi70433-bib-0038] Liang, Q. , H. Li , S. Li , et al. 2019. “The Genome Assembly and Annotation of Yellowhorn (*Xanthoceras sorbifolium* Bunge).” GigaScience 8: 1–15. 10.1093/gigascience/giz071 PMC6593362 31241155

[pbi70433-bib-0039] Lin, Y. , J. Min , R. Lai , et al. 2017. “Genome‐Wide Sequencing of Longan (*Dimocarpus longan* Lour.) Provides Insights Into Molecular Basis of Its Polyphenol‐Rich Characteristics.” GigaScience 6: 1–14. 10.1093/gigascience/gix023 PMC5467034 28368449

[pbi70433-bib-0040] Lin, Y. , C. Ye , X. Li , et al. 2023. “QuarTeT: A Telomere‐to‐Telomere Toolkit for Gap‐Free Genome Assembly and Centromeric Repeat Identification.” Horticultural Research 10: 1–7. 10.1093/hr/uhad127 PMC10407605 37560017

[pbi70433-bib-0041] Liu, D. , J. Li , S. Wang , et al. 2024. “PlantCFG: A Comprehensive Database With Web Tools for Analyzing Candidate Flowering Genes in Multiple Plants.” Plant Communications 5: 100733. 37849251 10.1016/j.xplc.2023.100733 PMC10873886

[pbi70433-bib-0042] Love, M. I. , S. Anders , and W. Huber . 2014. “Differential Analysis of Count Data – The DESeq2 Package.”

[pbi70433-bib-0043] Lowry, D. B. , and J. H. Willis . 2010. “A Widespread Chromosomal Inversion Polymorphism Contributes to a Major Life‐History Transition, Local Adaptation, and Reproductive Isolation.” PLoS Biology 8: e1000500. 20927411 10.1371/journal.pbio.1000500 PMC2946948

[pbi70433-bib-0044] Luo, X. , T. Chen , X. Zeng , D. He , and Y. He . 2019. “Feedback Regulation of FLC by FLOWERING LOCUS T (FT) and FD Through a 5′ FLC Promoter Region in Arabidopsis.” Molecular Plant 12: 285–288. 30685381 10.1016/j.molp.2019.01.013

[pbi70433-bib-0045] Magome, H. , S. Yamaguchi , A. Hanada , Y. Kamiya , and K. Oda . 2008. “The DDF1 Transcriptional Activator Upregulates Expression of a Gibberellin‐Deactivating Gene, GA2ox7, Under High‐Salinity Stress in Arabidopsis.” Plant Journal 56: 613–626. 10.1111/j.1365-313X.2008.03627.x 18643985

[pbi70433-bib-0046] Mandáková, T. , M. Pouch , J. R. Brock , I. A. Al‐Shehbaz , and M. A. Lysak . 2019. “Origin and Evolution of Diploid and Allopolyploid Camelina Genomes Were Accompanied by Chromosome Shattering.” Plant Cell 31: 2596–2612. 31451448 10.1105/tpc.19.00366 PMC6881126

[pbi70433-bib-0047] Manni, M. , M. R. Berkeley , M. Seppey , and E. M. Zdobnov . 2021. “BUSCO: Assessing Genomic Data Quality and Beyond.” Current Protocols 1: 1–41. 10.1002/cpz1.323 34936221

[pbi70433-bib-0048] Mendes, F. K. , D. Vanderpool , B. Fulton , and M. W. Hahn . 2020. “CAFE 5 Models Variation in Evolutionary Rates Among Gene Families.” Bioinformatics 36: 5516–5518. 10.1093/bioinformatics/btaa1022 33325502

[pbi70433-bib-0049] Moreau, F. , E. Thévenon , R. Blanvillain , et al. 2016. “The Myb‐Domain Protein ULTRAPETALA1 INTERACTING FACTOR 1 Controls Floral Meristem Activities in Arabidopsis.” Development 143: 1108–1119. 26903506 10.1242/dev.127365

[pbi70433-bib-0050] Mu, H. , J. Chen , W. Huang , et al. 2024. “OmicShare Tools: A Zero‐Code Interactive Online Platform for Biological Data Analysis and Visualization.” iMeta 3: e228. 39429881 10.1002/imt2.228 PMC11488081

[pbi70433-bib-0051] Olszewski, N. , T. P. Sun , and F. Gubler . 2002. “Gibberellin Signaling: Biosynthesis, Catabolism, and Response Pathways.” Plant Cell 14: 61–80. 10.1105/tpc.010476 PMC151248 12045270

[pbi70433-bib-0052] Osnato, M. , C. Castillejo , L. Matías‐Hernández , and S. Pelaz . 2012. “TEMPRANILLO Genes Link Photoperiod and Gibberellin Pathways to Control Flowering in Arabidopsis.” Nature Communications 3: 808. 10.1038/ncomms1810 22549837

[pbi70433-bib-0053] Pan, L. , M. Liu , Y. Kang , et al. 2023. “Comprehensive Genomic Analyses of Vigna Unguiculata Provide Insights Into Population Differentiation and the Genetic Basis of Key Agricultural Traits.” Plant Biotechnology Journal 21: 1426–1439. 36965079 10.1111/pbi.14047 PMC10281604

[pbi70433-bib-0054] Pham, V. T. , M. Herrero , and J. I. Hormaza . 2015. “Phenological Growth Stages of Longan (*Dimocarpus longan*) According to the BBCH Scale.” Scientia Horticulturae 189: 201–207.

[pbi70433-bib-0055] Rhie, A. , B. P. Walenz , S. Koren , and A. M. Phillippy . 2020. “Merqury: Reference‐Free Quality, Completeness, and Phasing Assessment for Genome Assemblies.” Genome Biology 21: 245. 32928274 10.1186/s13059-020-02134-9 PMC7488777

[pbi70433-bib-0056] Ritchie, M. E. , B. Phipson , D. Wu , et al. 2015. “Limma Powers Differential Expression Analyses for RNA‐Sequencing and Microarray Studies.” Nucleic Acids Research 43: e47. 25605792 10.1093/nar/gkv007 PMC4402510

[pbi70433-bib-0057] Sai‐Ut, S. , P. Kingwascharapong , M. A. R. Mazumder , and S. Rawdkuen . 2023. “Optimization of Ethanolic Extraction of Phenolic Antioxidants From Lychee and Longan Seeds Using Response Surface Methodology.” Food 12: 2827. 10.3390/foods12152827 PMC10417469 37569096

[pbi70433-bib-0058] Servant, N. , N. Varoquaux , B. R. Lajoie , et al. 2015. “HiC‐Pro: An Optimized and Flexible Pipeline for Hi‐C Data Processing.” Genome Biology 16: 1–11. 26619908 10.1186/s13059-015-0831-x PMC4665391

[pbi70433-bib-0059] Shen, Y. , T. Sun , Q. Pan , et al. 2019. “RrMYB5‐ and RrMYB10‐Regulated Flavonoid Biosynthesis Plays a Pivotal Role in Feedback Loop Responding to Wounding and Oxidation in *Rosa rugosa* .” Plant Biotechnology Journal 17: 2078–2095. 30951245 10.1111/pbi.13123 PMC6790370

[pbi70433-bib-0060] Shi, M. , C. Wang , P. Wang , et al. 2023. “Role of Methylation in Vernalization and Photoperiod Pathway: A Potential Flowering Regulator?” Horticulture Research 10: uhad174. 37841501 10.1093/hr/uhad174 PMC10569243

[pbi70433-bib-0061] Shi, M. , C. Wang , P. Wang , M. Zhang , and W. Liao . 2022. “Methylation in DNA, Histone, and RNA During Flowering Under Stress Condition: A Review.” Plant Science 324: 111431. 36028071 10.1016/j.plantsci.2022.111431

[pbi70433-bib-0062] Song, Y. H. , D. A. Estrada , R. S. Johnson , et al. 2014. “Distinct Roles of FKF1, GIGANTEA, and ZEITLUPE Proteins in the Regulation of Constans Stability in Arabidopsis Photoperiodic Flowering.” Proceedings of the National Academy of Sciences of the United States of America 111: 17672–17677. 25422419 10.1073/pnas.1415375111 PMC4267339

[pbi70433-bib-0063] South Subtropical Crops Center, M. of A. and R.A . 2024. “Report on the Development of China's Litchi and Longan Industries.” China Tropical Agriculture 3: 5–7.

[pbi70433-bib-0064] Stanke, M. , O. Keller , I. Gunduz , A. Hayes , S. Waack , and B. Morgenstern . 2006. “AUGUSTUS: ab Initio Prediction of Alternative Transcripts.” Nucleic Acids Research 34: 435–439. 10.1093/nar/gkl200 PMC1538822 16845043

[pbi70433-bib-0065] Streitner, C. , S. Danisman , F. Wehrle , J. C. Schöning , J. R. Alfano , and D. Staiger . 2008. “The Small Glycine‐Rich RNA Binding Protein AtGRP7 Promotes Floral Transition in *Arabidopsis thaliana* .” Plant Journal 56: 239–250. 10.1111/j.1365-313X.2008.03591.x 18573194

[pbi70433-bib-0066] Tang, H. , V. Krishnakumar , X. Zeng , et al. 2024. “JCVI: A Versatile Toolkit for Comparative Genomics Analysis.” iMeta 3: 1–13. 10.1002/imt2.211 PMC11316928 39135687

[pbi70433-bib-0067] Wang, B. , S. H. Jin , H. Q. Hu , et al. 2012. “UGT87A2, an Arabidopsis Glycosyltransferase, Regulates Flowering Time via FLOWERING LOCUS C.” New Phytologist 194: 666–675. 22404750 10.1111/j.1469-8137.2012.04107.x

[pbi70433-bib-0068] Wang, J. , H. Hu , X. Liang , et al. 2023. “High‐Quality Genome Assembly and Comparative Genomic Profiling of Yellowhorn (*Xanthoceras sorbifolia*) Revealed Environmental Adaptation Footprints and Seed Oil Contents Variations.” Frontiers in Plant Science 14: 1–10. 10.3389/fpls.2023.1147946 PMC10070836 37025151

[pbi70433-bib-0069] Wang, J. , J. Li , Z. Li , et al. 2022. “Genomic Insights Into Longan Evolution From a Chromosome‐Level Genome Assembly and Population Genomics of Longan Accessions.” Horticultural Research 9: 1–14. 10.1093/hr/uhac021 PMC9071379 35184175

[pbi70433-bib-0070] Wang, P. , X. Cui , C. Zhao , et al. 2017. “COR27 and COR28 Encode Nighttime Repressors Integrating Arabidopsis Circadian Clock and Cold Response.” Journal of Integrative Plant Biology 59: 78–85. 27990760 10.1111/jipb.12512

[pbi70433-bib-0071] Wang, S. , H. Wang , F. Jiang , et al. 2022. “EndHiC: Assemble Large Contigs Into Chromosome‐Level Scaffolds Using the Hi‐C Links From Contig Ends.” BMC Bioinformatics 23: 1–19. 36482318 10.1186/s12859-022-05087-x PMC9730666

[pbi70433-bib-0072] Wang, X. , Y. Zhang , Q. Ma , et al. 2007. “SKB1‐Mediated Symmetric Dimethylation of Histone H4R3 Controls Flowering Time in Arabidopsis.” EMBO Journal 26: 1934–1941. 17363895 10.1038/sj.emboj.7601647 PMC1847673

[pbi70433-bib-0073] Westermann, J. , T. Srikant , A. Gonzalo , H. S. Tan , and K. Bomblies . 2024. “Defective Pollen Tube Tip Growth Induces Neo‐Polyploid Infertility.” Science 383: 1–62. 10.1126/science.adh0755 38422152

[pbi70433-bib-0074] Wu, T. , Z. Liu , T. Yu , et al. 2024. “Flowering Genes Identification, Network Analysis, and Database Construction for 837 Plants.” Horticulture Research 11: 1–13. 10.1093/hr/uhae013 PMC10995624 38585015

[pbi70433-bib-0075] Wu, Z. , X. Fang , D. Zhu , and C. Dean . 2020. “Autonomous Pathway: Flowering Locus c Repression Through an Antisense‐Mediated Chromatin‐Silencing Mechanism.” Plant Physiology 182: 27–37. 31740502 10.1104/pp.19.01009 PMC6945862

[pbi70433-bib-0076] Xue, T. , D. Chen , T. Zhang , et al. 2022. “Chromosome‐Scale Assembly and Population Diversity Analyses Provide Insights Into the Evolution of *Sapindus mukorossi* .” Horticultural Research 9: 1–14. 10.1093/hr/uhac012 PMC8854635 35178562

[pbi70433-bib-0077] Yaish, M. W. , J. Colasanti , and S. J. Rothstein . 2011. “The Role of Epigenetic Processes in Controlling Flowering Time in Plants Exposed to Stress.” Journal of Experimental Botany 62: 3727–3735. 21633082 10.1093/jxb/err177

[pbi70433-bib-0078] Yamaguchi, N. , C. M. Winter , M. F. Wu , et al. 2014. “Gibberellin Acts Positively Then Negatively to Control Onset of Flower Formation in Arabidopsis.” Science 344: 638–641. 24812402 10.1126/science.1250498

[pbi70433-bib-0079] Yan, C. , Z. Zhao , and Z. Zhang . 1998. “Effects of Chemical on Introducing Blossom of Longan.” Journal of the Chinese Society for Horticultural Science 44: 517–518.

[pbi70433-bib-0080] Yan, H. , A. Bombarely , and S. Li . 2020. “DeepTE: A Computational Method for de Novo Classification of Transposons With Convolutional Neural Network.” Bioinformatics 36: 4269–4275. 32415954 10.1093/bioinformatics/btaa519

[pbi70433-bib-0081] Yang, J. , H. M. Wariss , L. Tao , et al. 2019. “De Novo Genome Assembly of the Endangered Acer Yangbiense, a Plant Species With Extremely Small Populations Endemic to Yunnan Province, China.” GigaScience 8: 1–10. 10.1093/gigascience/giz085 PMC6629541 31307060

[pbi70433-bib-0082] Yang, Z. 2007. “PAML 4: Phylogenetic Analysis by Maximum Likelihood.” Molecular Biology and Evolution 24: 1586–1591. 17483113 10.1093/molbev/msm088

[pbi70433-bib-0083] Yang, Z. , C. Xingxing , L. Zhang , H. Jiwang , C. Yeyuan , and L. Songgang . 2015. “Relationship Between Longan Flower and Oxidative Damage.” Chinese Journal of Tropical Crops 036: 120–124.

[pbi70433-bib-0084] Yoo, S. K. , X. Wu , J. S. Lee , and J. H. Ahn . 2011. “AGAMOUS‐LIKE 6 Is a Floral Promoter That Negatively Regulates the FLC/MAF Clade Genes and Positively Regulates FT in Arabidopsis.” Plant Journal 65: 62–76. 10.1111/j.1365-313X.2010.04402.x 21175890

[pbi70433-bib-0085] Yu, T. , Y. Hu , Y. Zhang , et al. 2021. “Whole‐Genome Sequencing of *Acer catalpifolium* Reveals Evolutionary History of Endangered Species.” Genome Biology and Evolution 13: 1–16. 10.1093/gbe/evab271 PMC8677443 34878129

[pbi70433-bib-0086] Yuan, Y. , N. Zhou , S. Bai , et al. 2024. “Evolutionary and Integrative Analysis of the Gibberellin 20‐Oxidase, 3‐Oxidase, and 2‐Oxidase Gene Family in *Paeonia ostii*: Insight Into Their Roles in Flower Senescence.” Agronomy 14: 590.

[pbi70433-bib-0087] Yue, J. , Q. Chen , Y. Wang , et al. 2023. “Telomere‐to‐Telomere and Gap‐Free Reference Genome Assembly of the Kiwifruit *Actinidia chinensis* .” Horticultural Research 10: uhac264. 10.1093/hr/uhac264 PMC9909506 36778189

[pbi70433-bib-0088] Zeng, S. , K. Wang , X. Liu , Z. Hu , and L. Zhao . 2024. “Potential of Longan ( *Dimocarpus longan* Lour.) in Functional Food: A Review of Molecular Mechanism‐Directing Health Benefit Properties.” Food Chemistry 437: 137812. 37897820 10.1016/j.foodchem.2023.137812

[pbi70433-bib-0089] Zhang, H. , C. Ransom , P. Ludwig , and S. Van Nocker . 2003. “Genetic Analysis of Early Flowering Mutants in Arabidopsis Defines a Class of Pleiotropic Developmental Regulator Required for Expression of the Flowering‐Time Switch FLOWERING LOCUS C.” Genetics 164: 347–358. 12750345 10.1093/genetics/164.1.347 PMC1462564

[pbi70433-bib-0090] Zhang, L. , L. Chen , and D. Yu . 2018. “Transcription Factor WRKY75 Interacts With DELLA Proteins to Affect Flowering.” Plant Physiology 176: 790–803. 29133369 10.1104/pp.17.00657 PMC5761768

[pbi70433-bib-0091] Zhang, R. , G. Li , X. Wang , et al. 2022. “TEsorter: An Accurate and Fast Method to Classify LTR‐Retrotransposons in Plant Genomes.” Horticultural Research 9: uhac017. 10.1093/hr/uhac017 PMC9002660 35184178

[pbi70433-bib-0092] Zhang, W. , J. Lin , J. Li , et al. 2021. “Rambutan Genome Revealed Gene Networks for Spine Formation and Aril Development.” Plant Journal 108: 1037–1052. 10.1111/tpj.15491 34519122

[pbi70433-bib-0093] Zhao, F. , H. Zhang , T. Zhao , Z. Li , and D. Jiang . 2021. “The Histone Variant H3.3 Promotes the Active Chromatin State to Repress Flowering in Arabidopsis.” Plant Physiology 186: 2051–2063. 34618105 10.1093/plphys/kiab224 PMC8331167

[pbi70433-bib-0094] Zhao, Z. C. , G. B. Hu , F. C. Hu , H. C. Wang , Z. Y. Yang , and B. Lai . 2012. “The UDP Glucose: Flavonoid‐3‐O‐Glucosyltransferase (UFGT) Gene Regulates Anthocyanin Biosynthesis in Litchi (*Litchi chinesis* Sonn.) During Fruit Coloration.” Molecular Biology Reports 39: 6409–6415. 22447536 10.1007/s11033-011-1303-3

[pbi70433-bib-0095] Zhu, X. R. , H. Wang , J. Sun , B. Yang , X. W. Duan , and Y. M. Jiang . 2019. “Pericarp and Seed of Litchi and Longan Fruits: Constituent, Extraction, Bioactive Activity, and Potential Utilization.” Journal of Zhejiang University. Science. B 20: 503–512. 31090276 10.1631/jzus.B1900161 PMC6568221

