# Supplementary material for: Improved Longan Genome Assembly Reveals Insights Into Flowering Mechanisms
Source: Plant Biotechnol J. 2025 Nov 4;24(3):1678–96. doi: 10.1111/pbi.70433 (PMC12946498; doi:10.1111/pbi.70433)
Supplement: Supplementary file 1 — Figure S1: Workflow of high‐quality chromosome‐level genome assembly for longan ( Dimocarpus longan ) cultivar ‘Shixia’. Figure S2: Genome‐wide Hi‐C chromatin interaction map of longan cultivar ‘Shixia’. Figure S3: Telomeres (a) and centromeres (b) predictions in the longan genome. Figure S4: Dot plot of 15 longan chromosomes showing syntenic regions. Figure S5: Collinearity analysis of longan and four closely related species. Figure S6: Dot plot of longan and lychee ( Litchi chinensis ) showing collinearity. Figure S7: Dot plot of longan and rambutan ( Nephelium lappaceum ). Figure S8: Dot plot of longan and soapberry ( Sapindus mukorossi ) showing collinearity. Figure S9: Dot plot of longan and yellowhorn ( Xanthoceras sorbifolia ) showing collinearity. Figure S10: Dot plot of longan and five distant species showing low collinearity. Figure S11: Non‐repetitive and repetitive sequence length in the genomes of five closely related species. Figure S12: Density plots of TE insertion times for Copia and Gypsy types in longan, lychee, rambutan, soapberry and yellowhorn. Figure S13: Statistics of transposable element (TE) divergence in longan, lychee, rambutan, soapberry and yellowhorn based on sequence identity. Figure S14: GO and KEGG enrichment analysis of longan‐specific genes identified by BlastP using longan as the query and lychee as the database. Figure S15: GO and KEGG enrichment analysis of longan‐specific genes identified by BlastP using longan as the database and lychee as the query. Figure S16: GO and KEGG enrichment analysis of longan‐specific genes compared to lychee, identified by ortho groups. Figure S17: Phylogenetic tree and gene family expansion/contraction analysis in the evolutionary process of longan. Figure S18: Chromosomal distribution of NBS‐encoding genes in longan and related genera. Figure S19: GO and KEGG enrichment analysis of significantly expanded genes in longan. Figure S20: Expression analysis of flavonoid biosynthesis‐related genes in [file PBI-24-1678-s001.docx]

**Supporting figures: Figure S1 ~ S55**


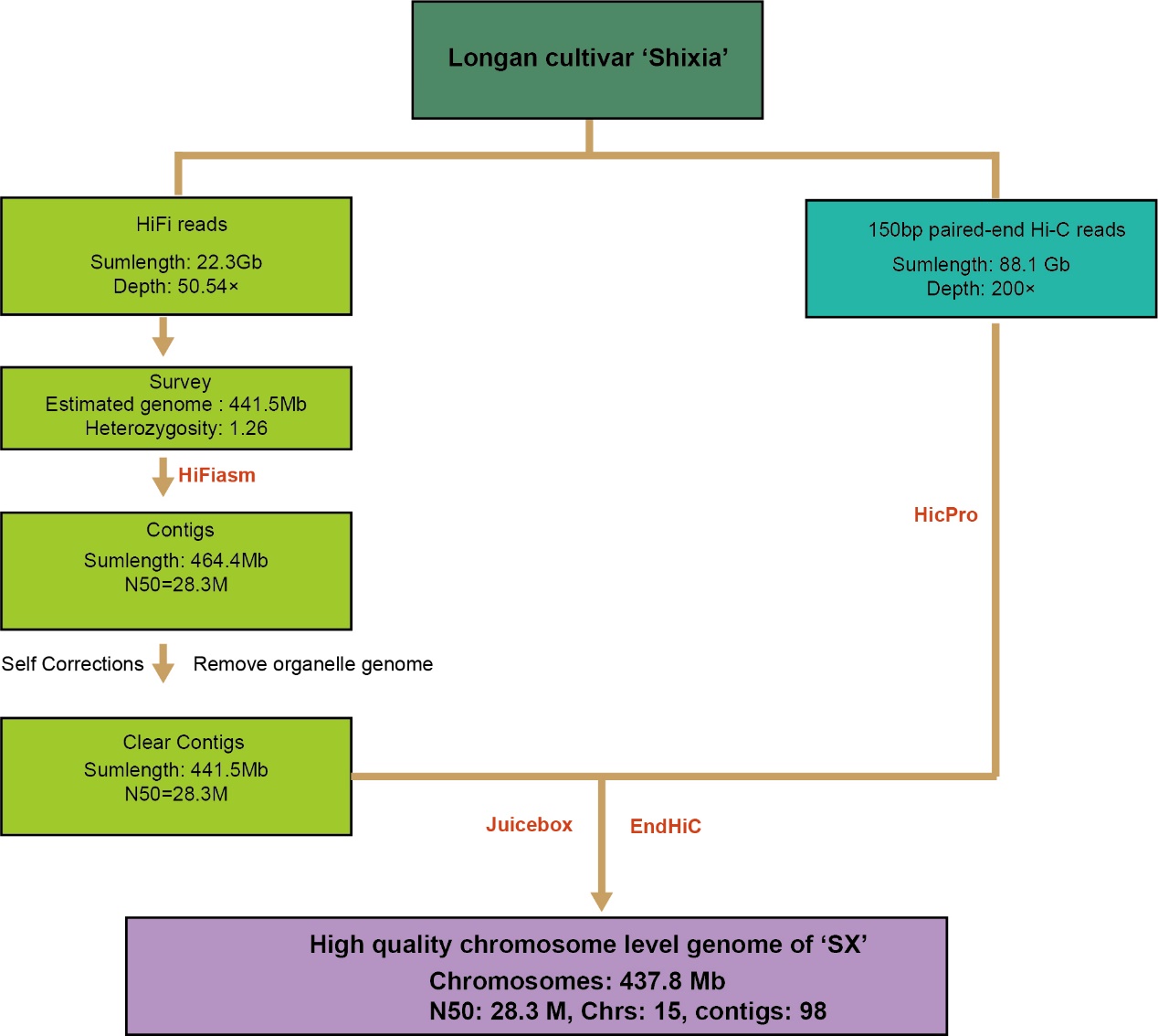


**Figure S1. Workflow of High-quality chromosome-level genome assembly for longan (*Dimocarpus longan*) cultivar ‘Shixia’.**

**
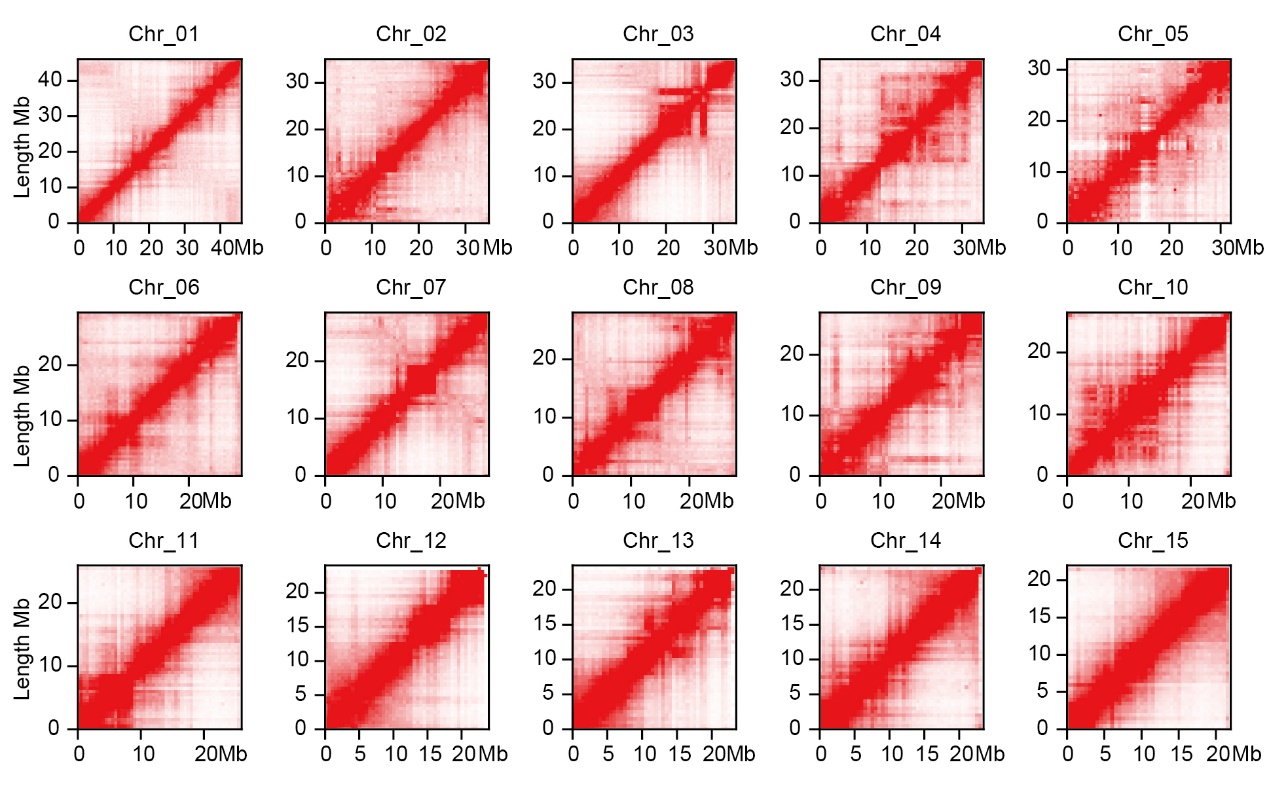
** **Figure S2. Genome-wide Hi-C chromatin interaction map of longan cultivar ‘Shixia’.**


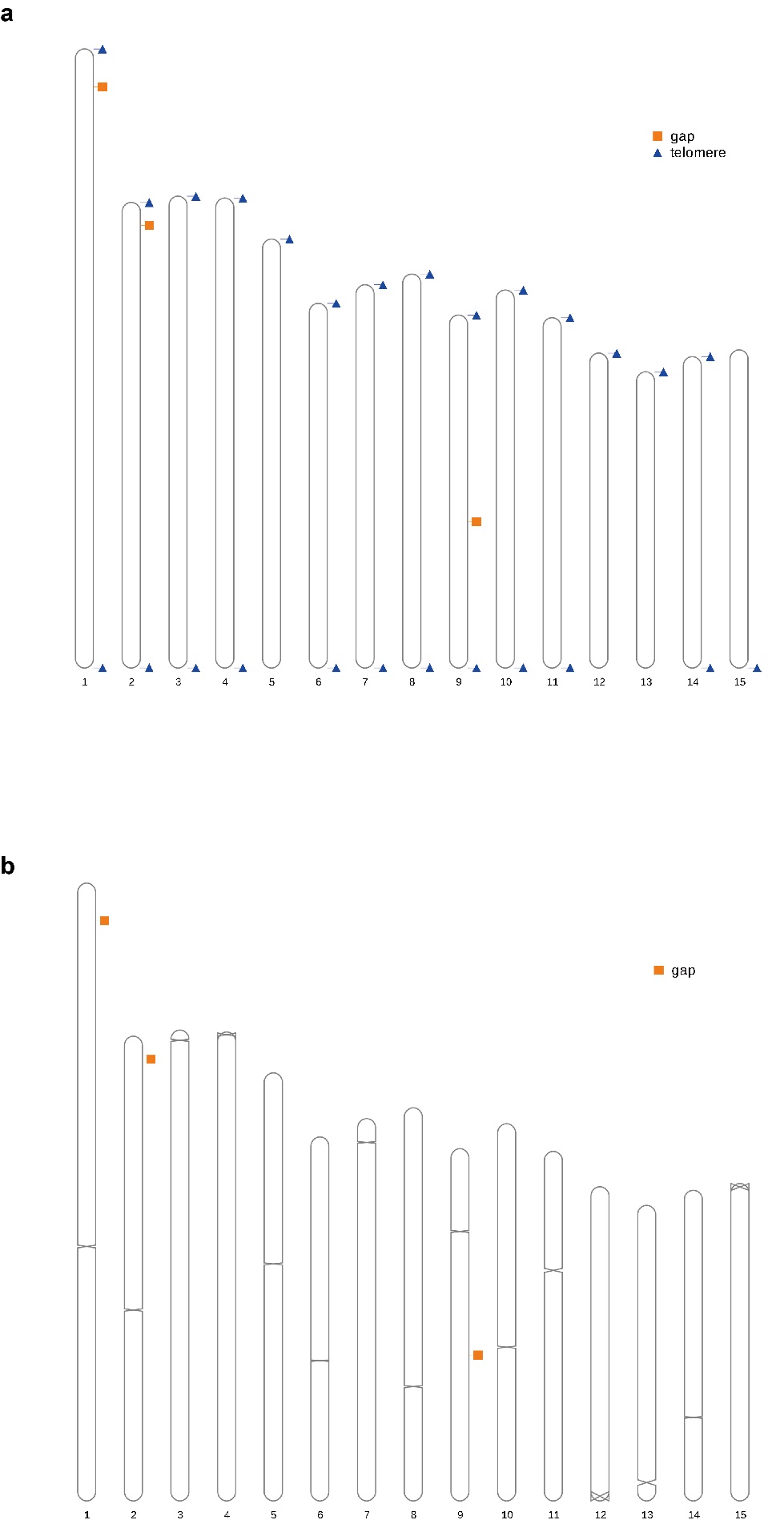


Figure S3. Telomeres (a) and centromeres (b) predictions in the longan genome.**
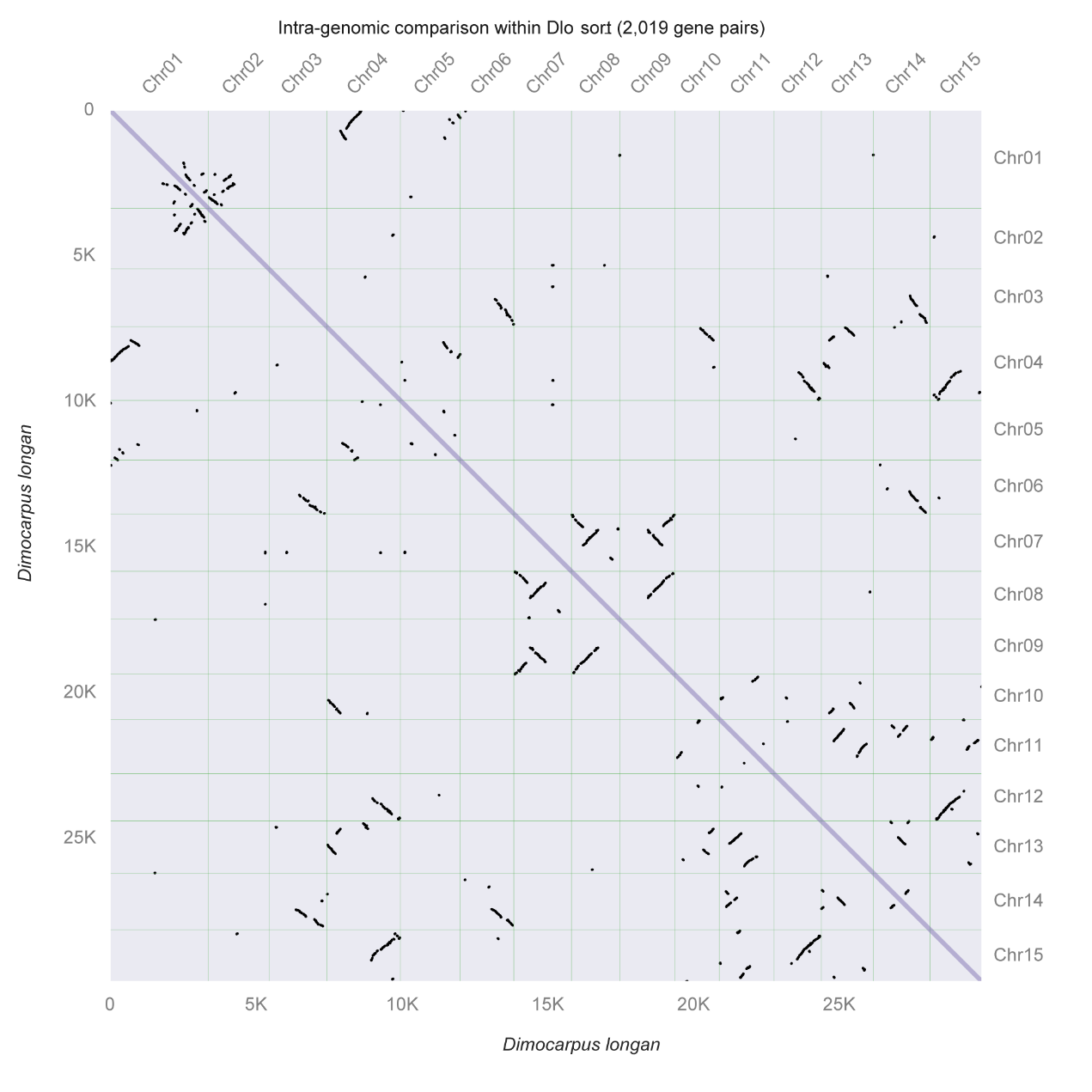
**

**Figure S4. Dot plot of 15 longan chromosomes showing syntenic regions.** Each chromosome exhibits syntenic regions with two other chromosomes, with major syntenic blocks spanning more than 30 genes.


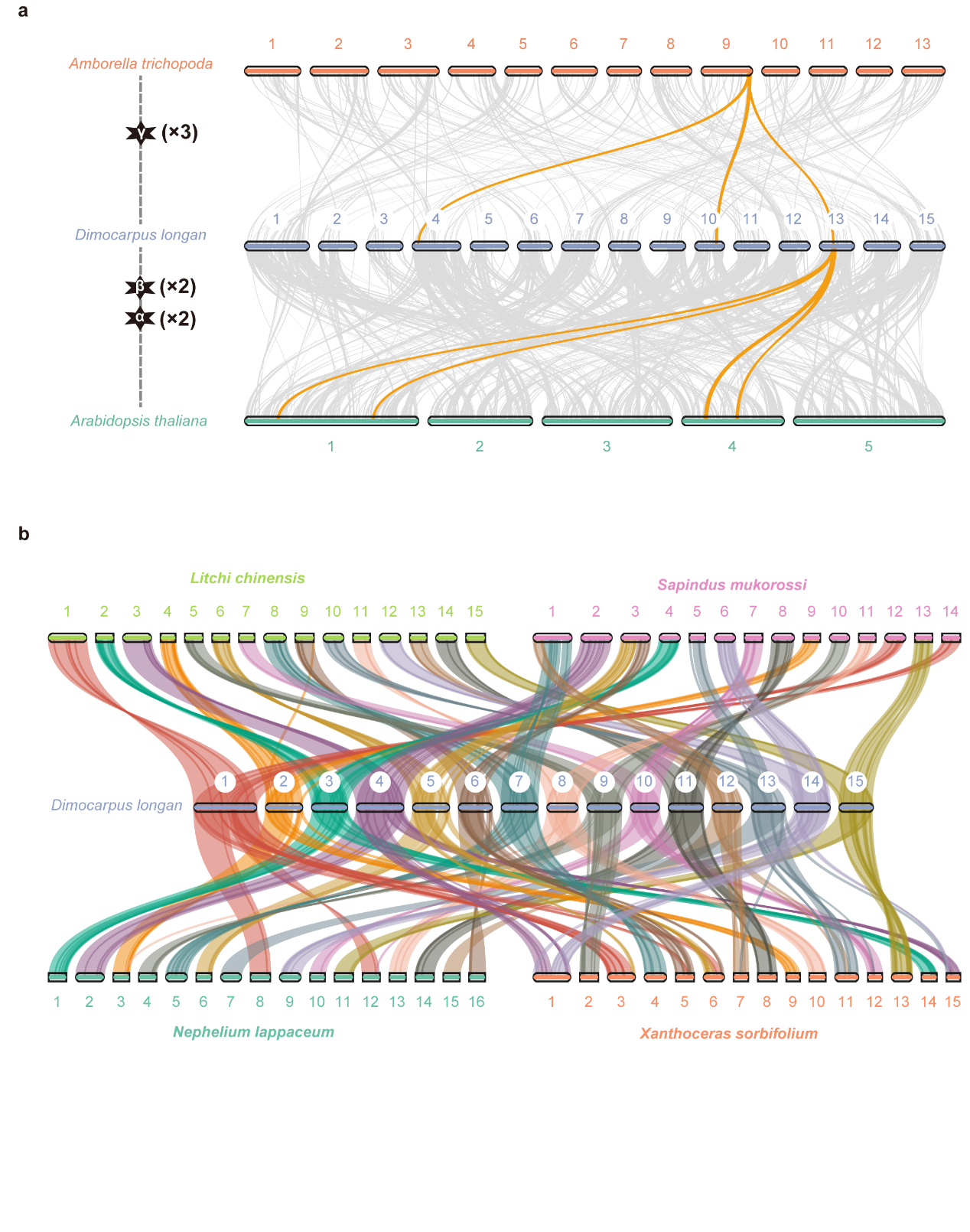
 **Figure S5. Collinearity analysis of longan and four closely related species.** Wedges highlight major syntenic blocks spanning more than 30 genes between the genomes.

**
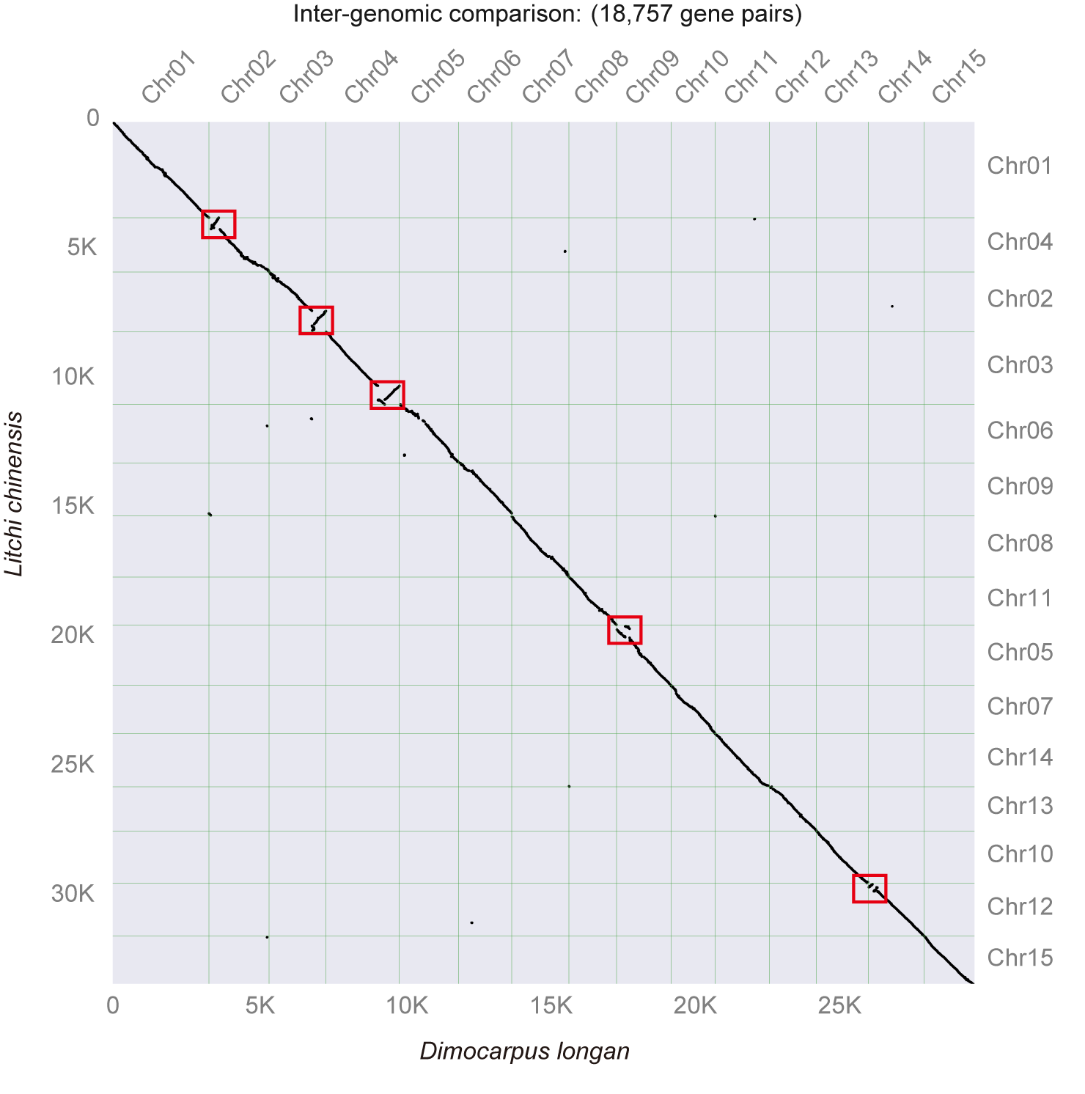
**

**Figure S6. Dot plot of longan and lychee (*Litchi chinensis*) showing collinearity.**

Longan and lychee exhibited high collinearity, except for four large inversion regions on Chr02, Chr03, Chr05, and Chr14, and a duplication on Chr09 in longan (indicated by the box). Major syntenic blocks spanning more than 30 genes are observed between the chromosomes.

**
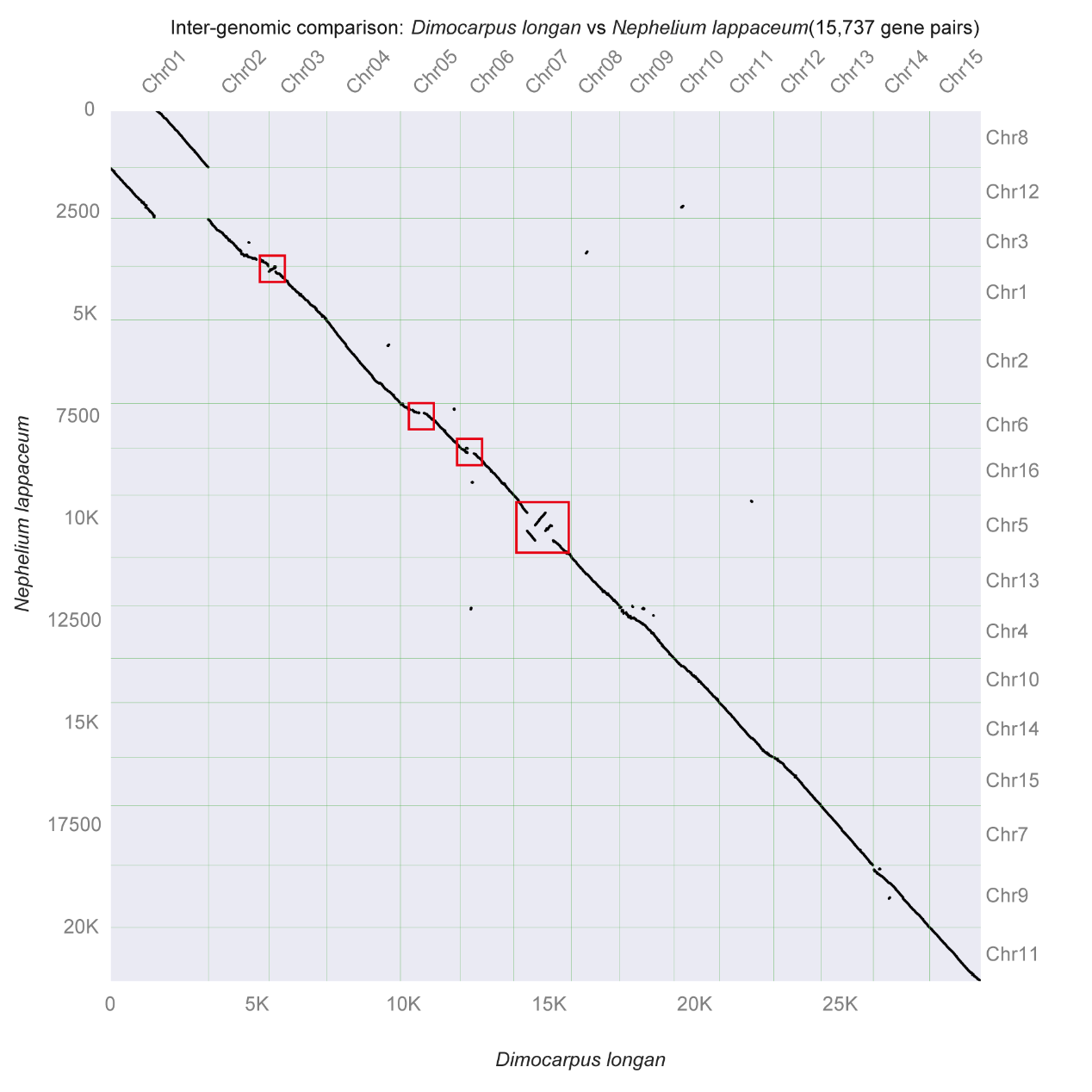
** **Figure S7.** **Dot-plot of longan and rambutan** (*Nephelium lappaceum*)**.** It indicated that longan and rambutan had significant collinearity, but Chr01 of longan had completely collinearity with 2 chromosomes of rambutan, and Chr03, Chr05, Chr06 and Chr07 of longan had significant inversion, translocation and deletion compared with rambutan (red box). The major syntenic blocks spanning more than 30 genes between the chromosomes.

**
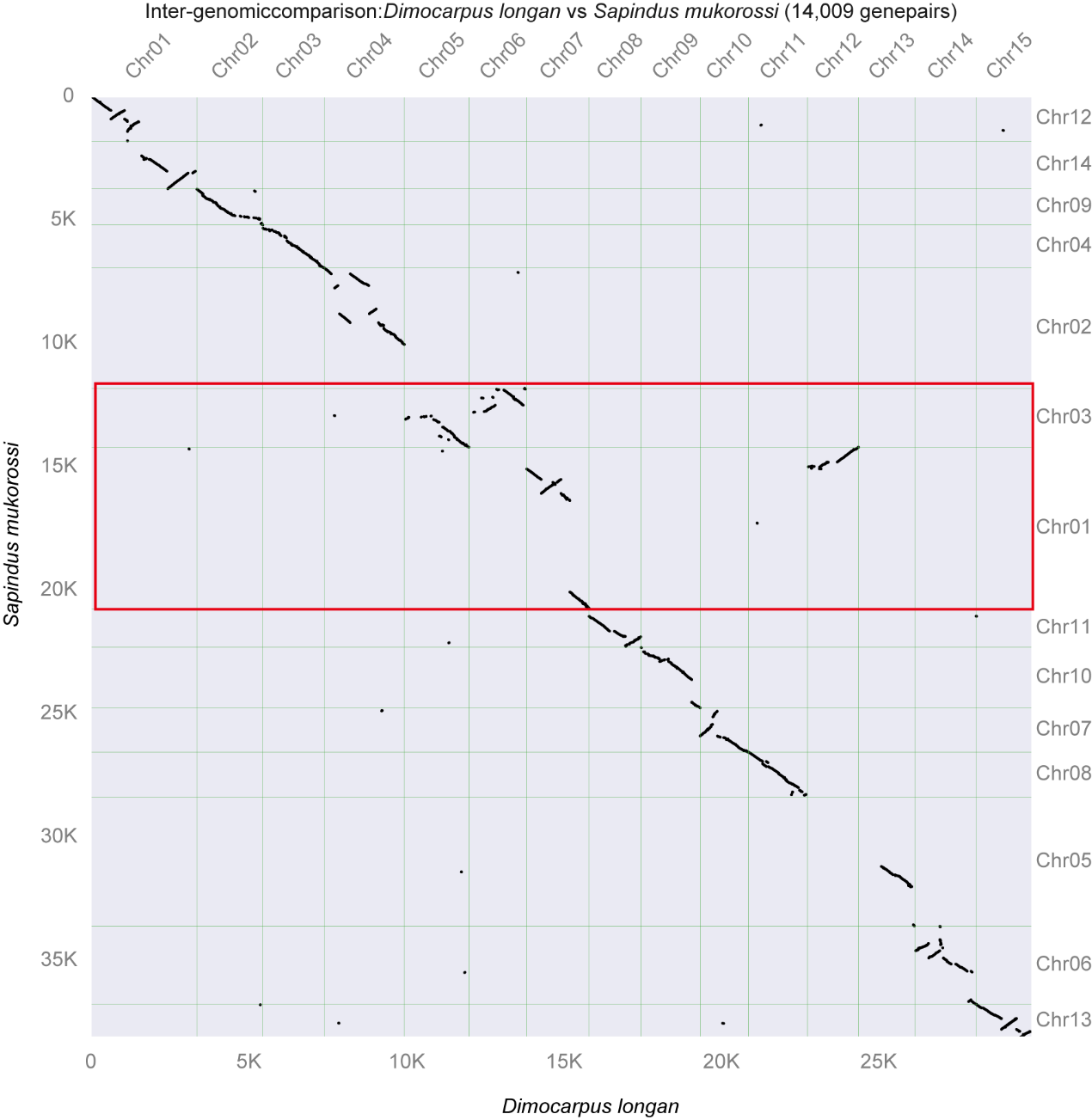
**

**Figure S8. Dot plot of longan and soapberry (*Sapindus mukorossi*) showing collinearity.** Longan and soapberry exhibited some collinearity, but significant chromosome inversions and translocations are observed in almost all chromosomes, except Chr02 and Chr03 of longan. Chr01 of longan was collinear with two chromosomes of soapberry, and Chr05, Chr06, Chr07, and Chr12 were collinear with one chromosome in soapberry (indicated by the red box). This suggested chromosome splitting and fusion in longan after its divergence from soapberry. Major syntenic blocks spanning more than 30 genes are observed between the chromosomes.

**
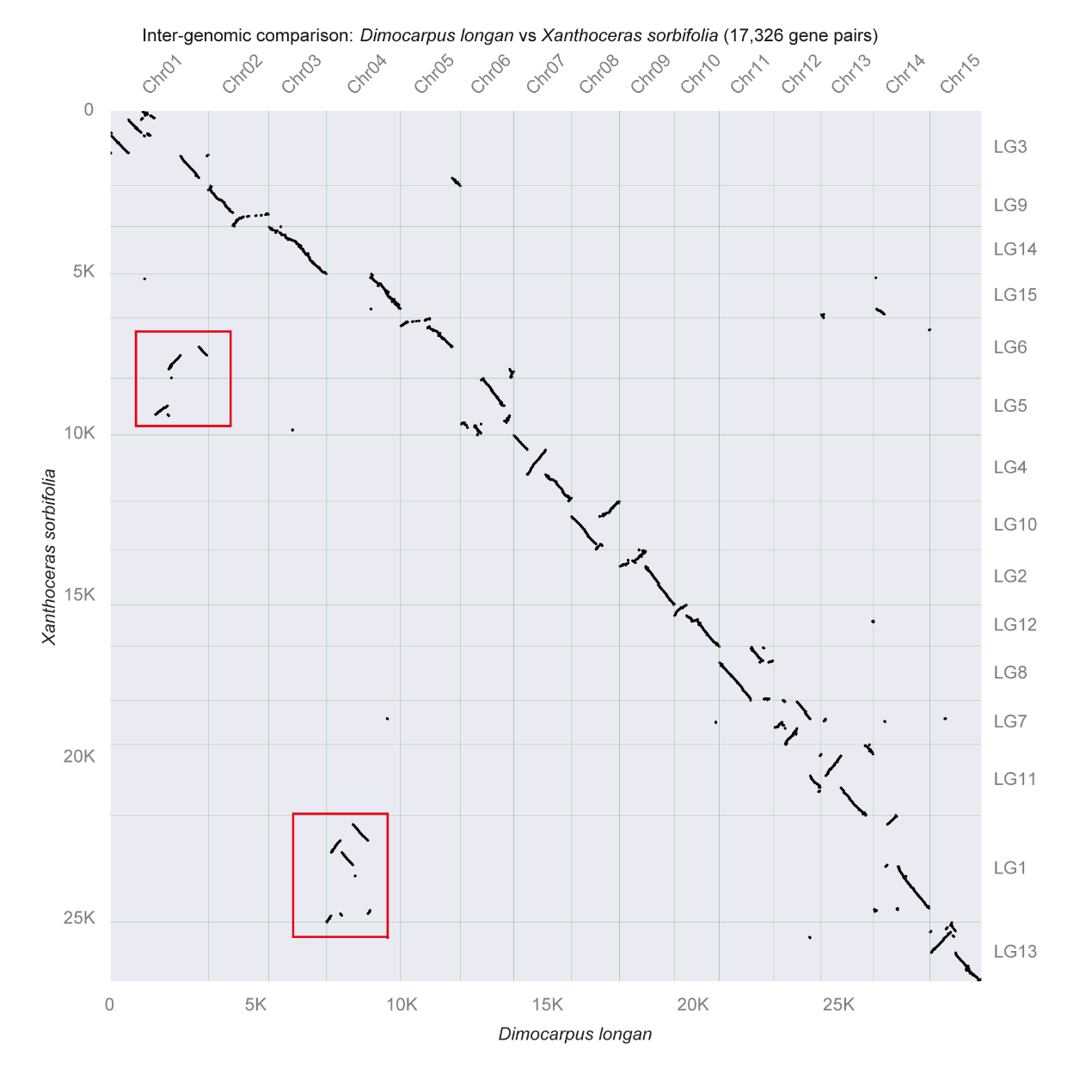
**

**Figure S9. Dot plot of longan and yellowhorn (*Xanthoceras sorbifolia*) showing collinearity.** Although longan and yellowhorn had the same number of chromosomes, they exhibited complex chromosome inversions and translocations in nearly all chromosomes, except for Chr03 in longan. Chr01 of longan was collinear with L3, LG6, and LG5 of yellowhorn, while Chr04 was collinear with LG15 and LG1 (indicated by the red box). Major syntenic blocks spanning more than 30 genes are observed between the chromosomes.

**
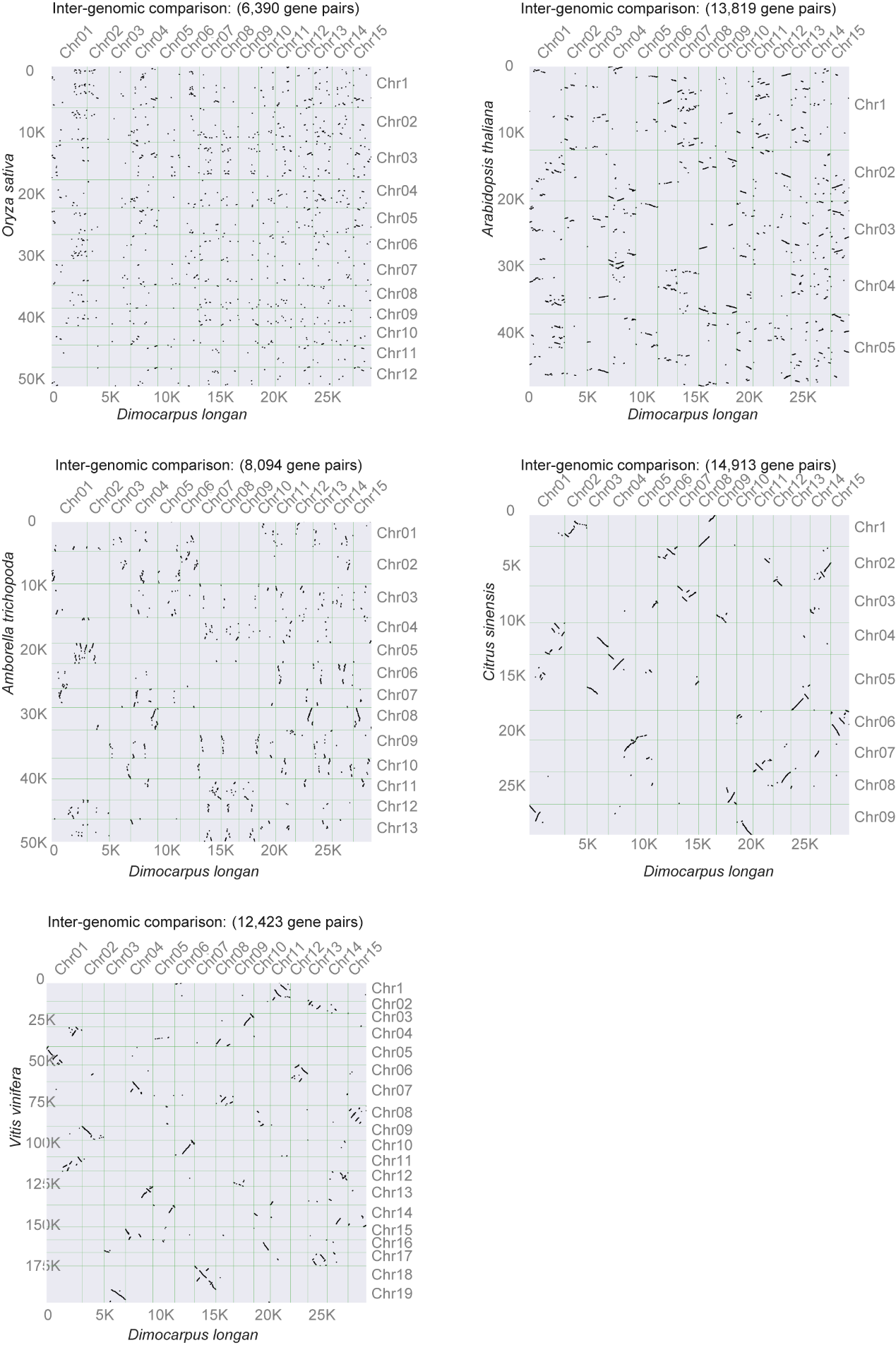
 Figure S10. Dot plot of longan and five distant species showing low collinearity.** Major syntenic blocks spanning more than 30 genes were observed between the chromosomes.

**
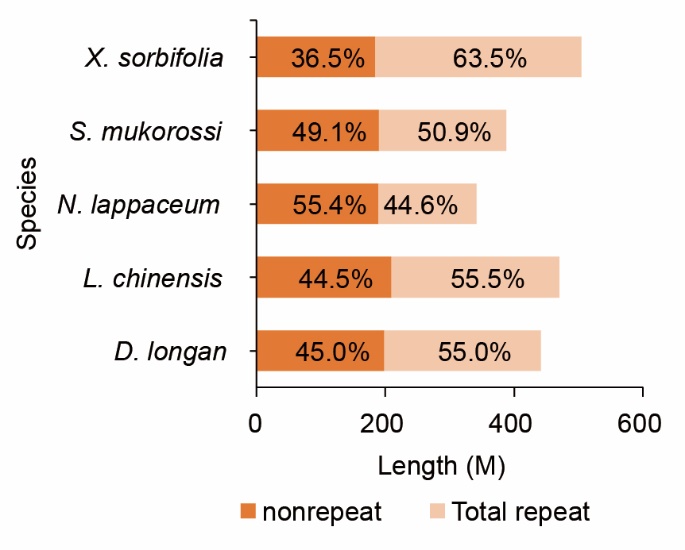
**

**Figure S11. Non-repetitive and repetitive sequence length in the genomes of five closely related species.** The species include lychee (*Litchi chinensis*), rambutan (*Nephelium lappaceum*), soapberry (*Sapindus mukorossi*) and yellowehorn (*Xanthoceras sorbifolia*).


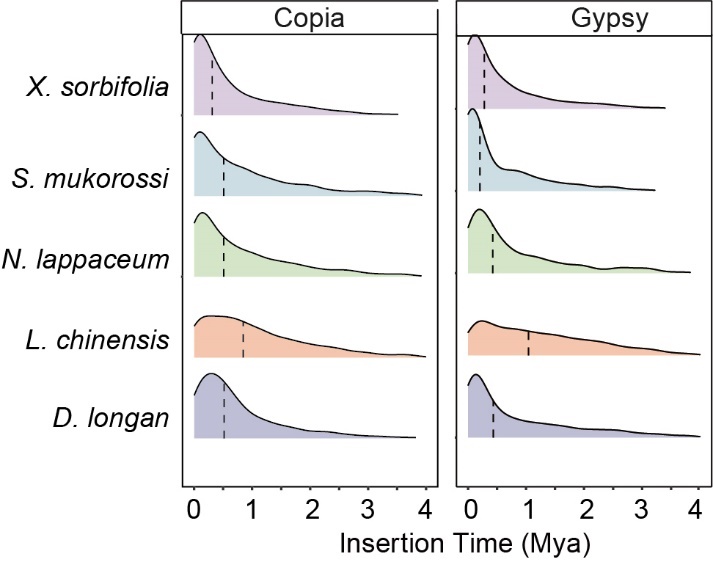


**Figure S12. Density plots of TE insertion times for Copia and Gypsy types in longan, lychee, rambutan, soapberry, and yellowhorn.** Insertion times were estimated based on intact LTR-RT insertions. A higher median in the density map indicates more recent insertion times.**
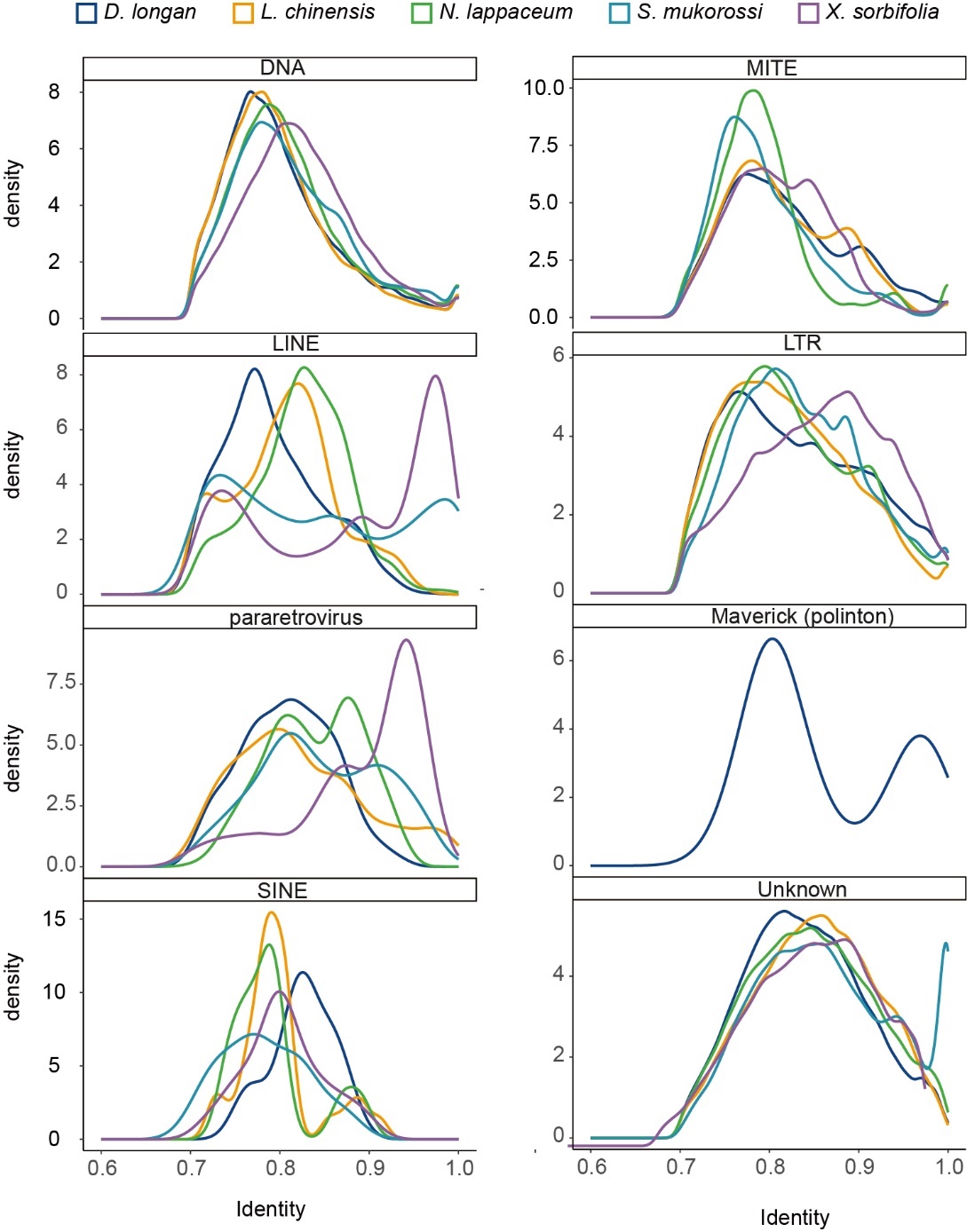
**

**Figure S13. Statistics of transposable element (TE) divergence in longan, lychee, rambutan, soapberry, and yellowhorn based on sequence identity.**

**
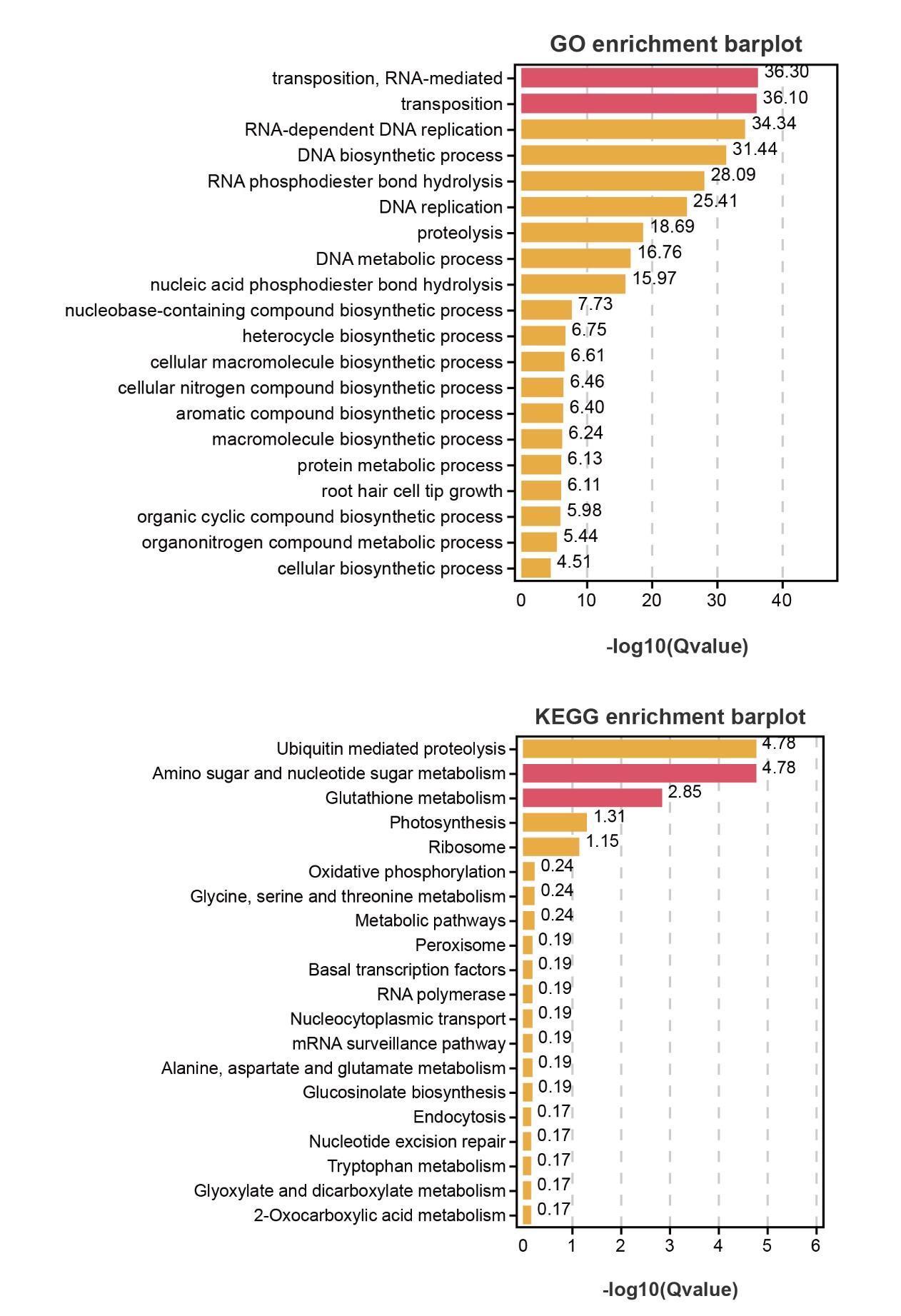
** **Figure S14. GO and KEGG enrichment analysis of longan-specific genes identified by BlastP using longan as the query and lychee as the database.** The top 20 enriched GO terms for biological processes (BP) and KEGG pathways are shown.

**
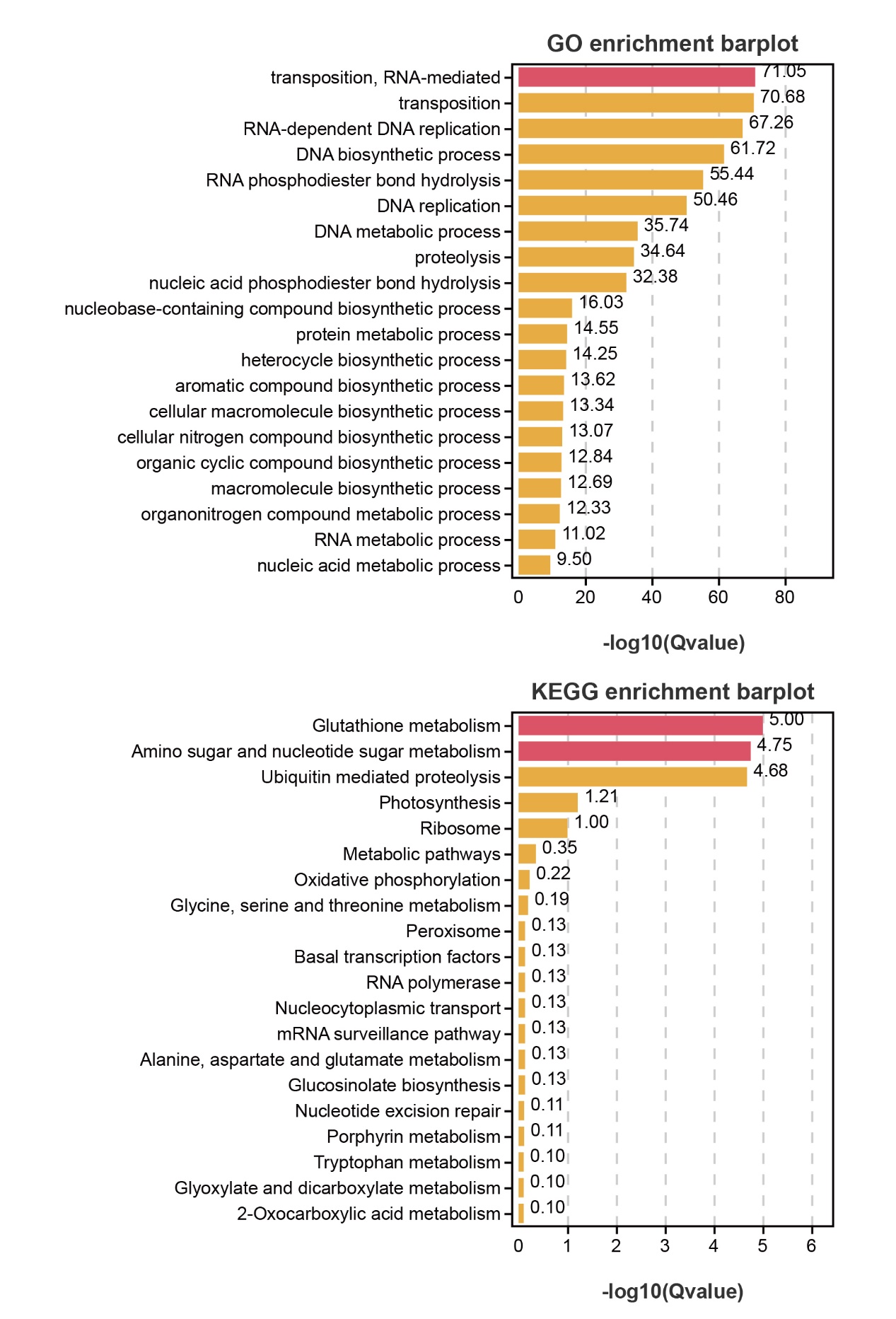
** **Figure S15. GO and KEGG enrichment analysis of longan-specific genes identified by BlastP using longan as the database and lychee as the query.** The top 20 enriched GO terms for biological processes (BP) and KEGG pathways were shown.

**
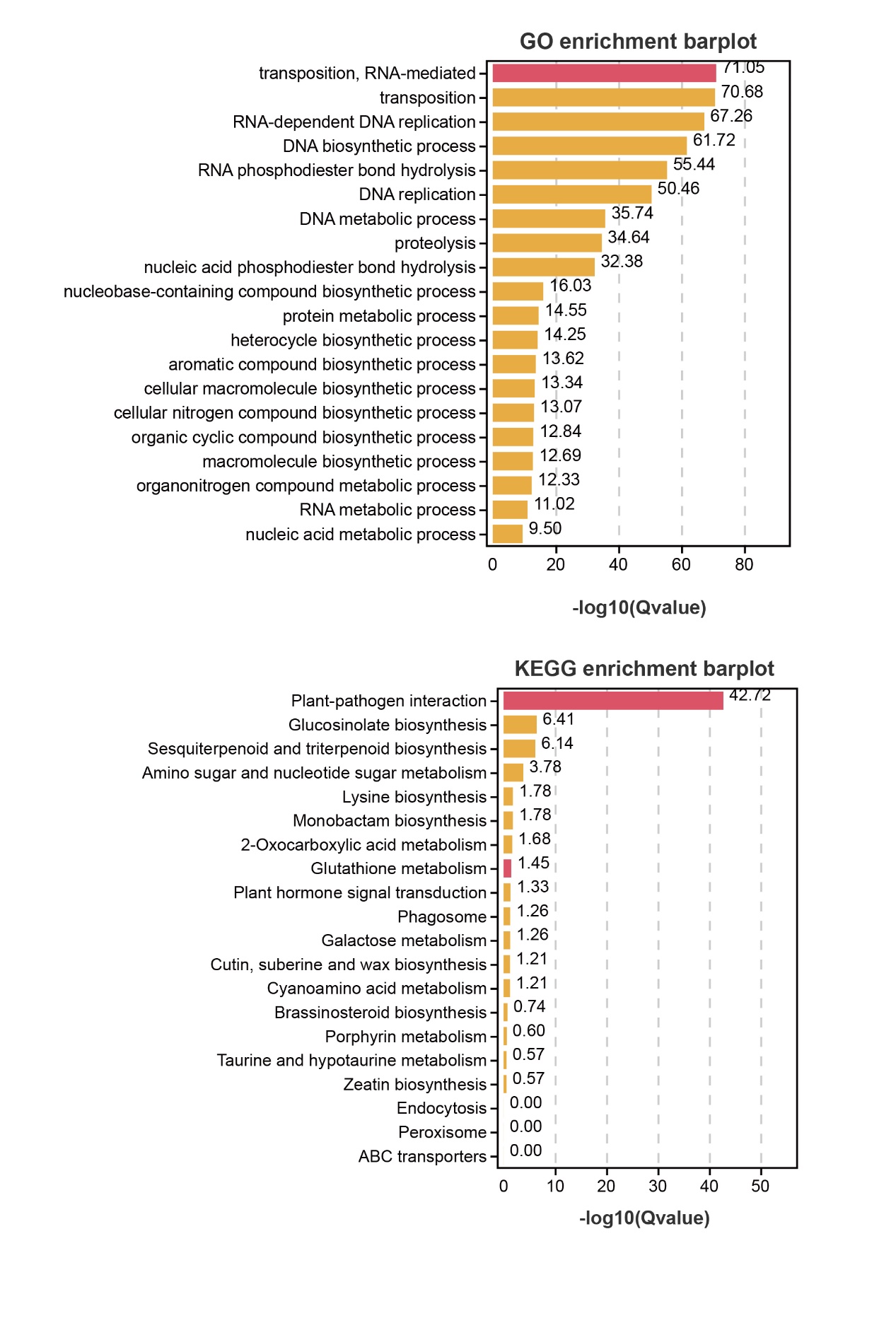
** **Figure S16. GO and KEGG enrichment analysis of longan-specific genes compared to lychee, identified by orthogroups.** The top 20 enriched GO terms for biological processes (BP) and KEGG pathways are shown.

**
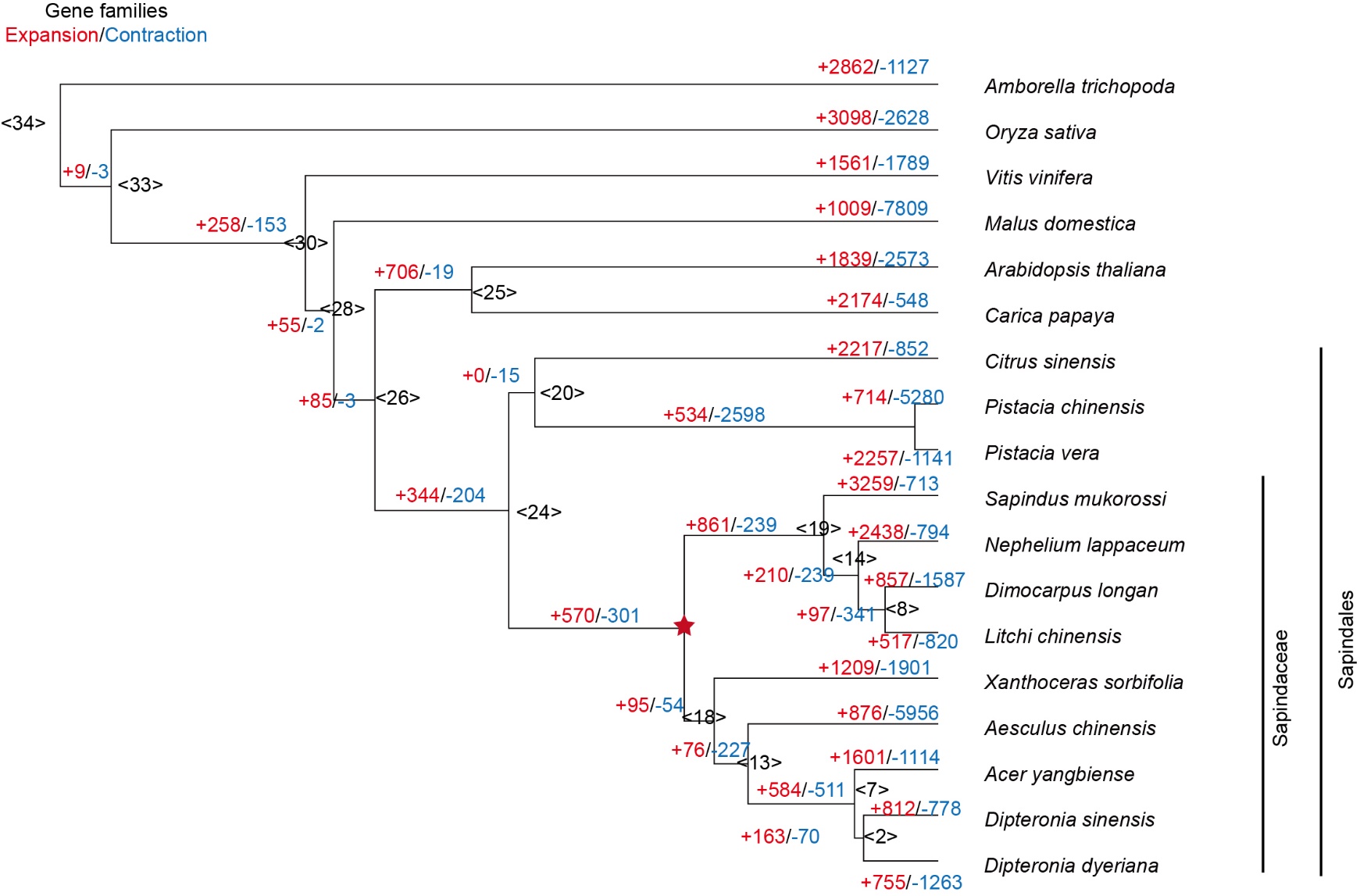
**

**Figure S17. Phylogenetic tree and gene family expansion/contraction analysis in the evolutionary process of longan.**

**
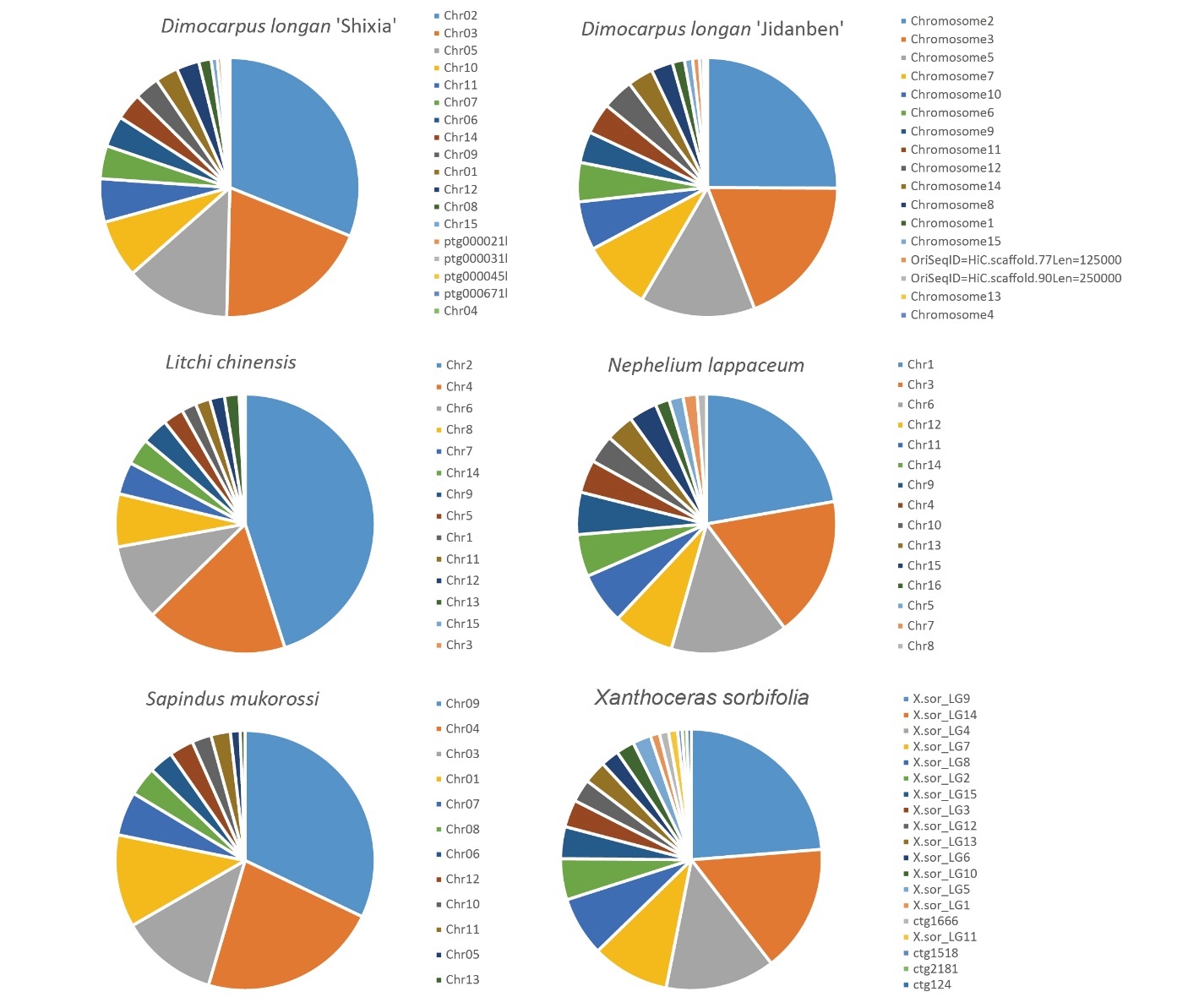
**

**Figure S18. Chromosomal distribution of NBS-encoding genes in longan and related genera.
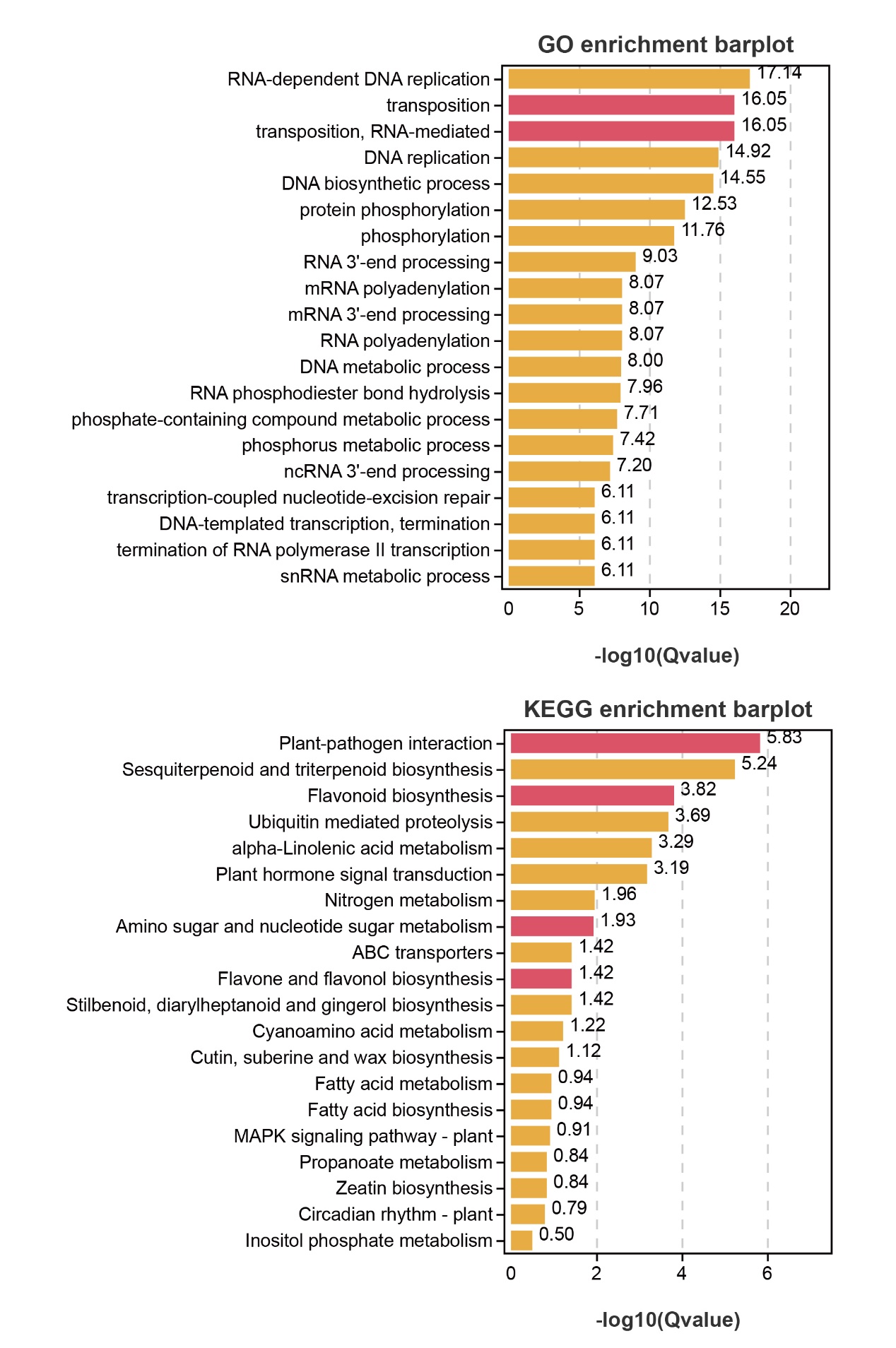
** **Figure S19. GO and KEGG enrichment analysis of significantly expanded genes in longan.** The top 20 enriched GO terms for biological processes (BP) and KEGG pathways are shown.

**
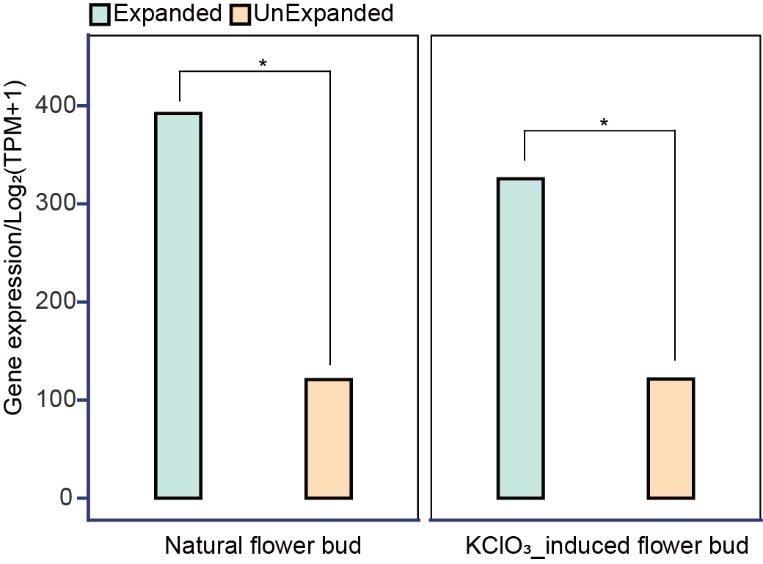
**

**Figure S20. Expression analysis of flavonoid biosynthesis-related genes in natural and potassium chlorate-induced flower buds.** Comparison between specifically expanded and non-significantly expanded genes. Significance was analyzed by *t*-test, with * indicating *P* < 0.05.

**
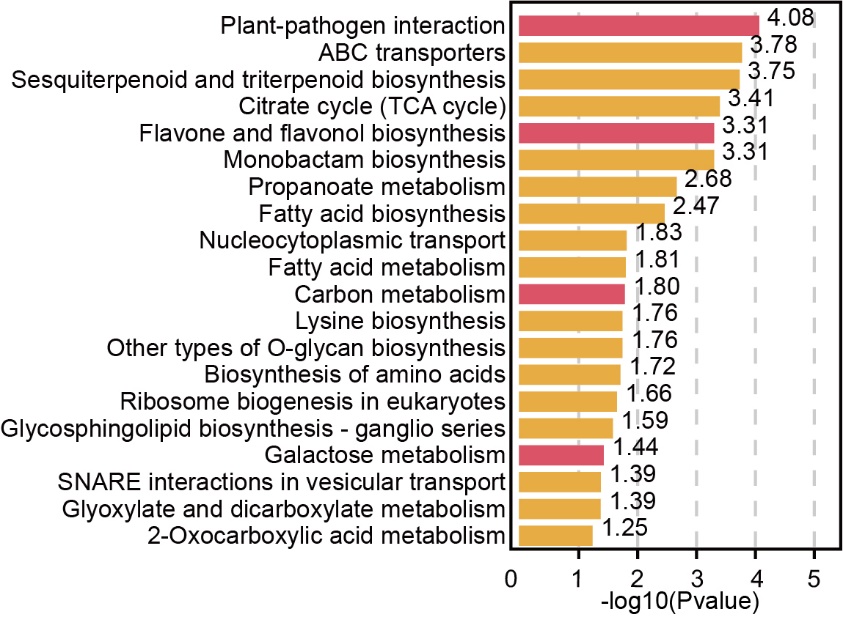
**

**Figure S21. Gene function enrichment of longan genes in inversion (INV) regions and structural variation analysis with lychee.** The top 20 GO terms for biological processes (BP) and KEGG pathways are displayed.

**
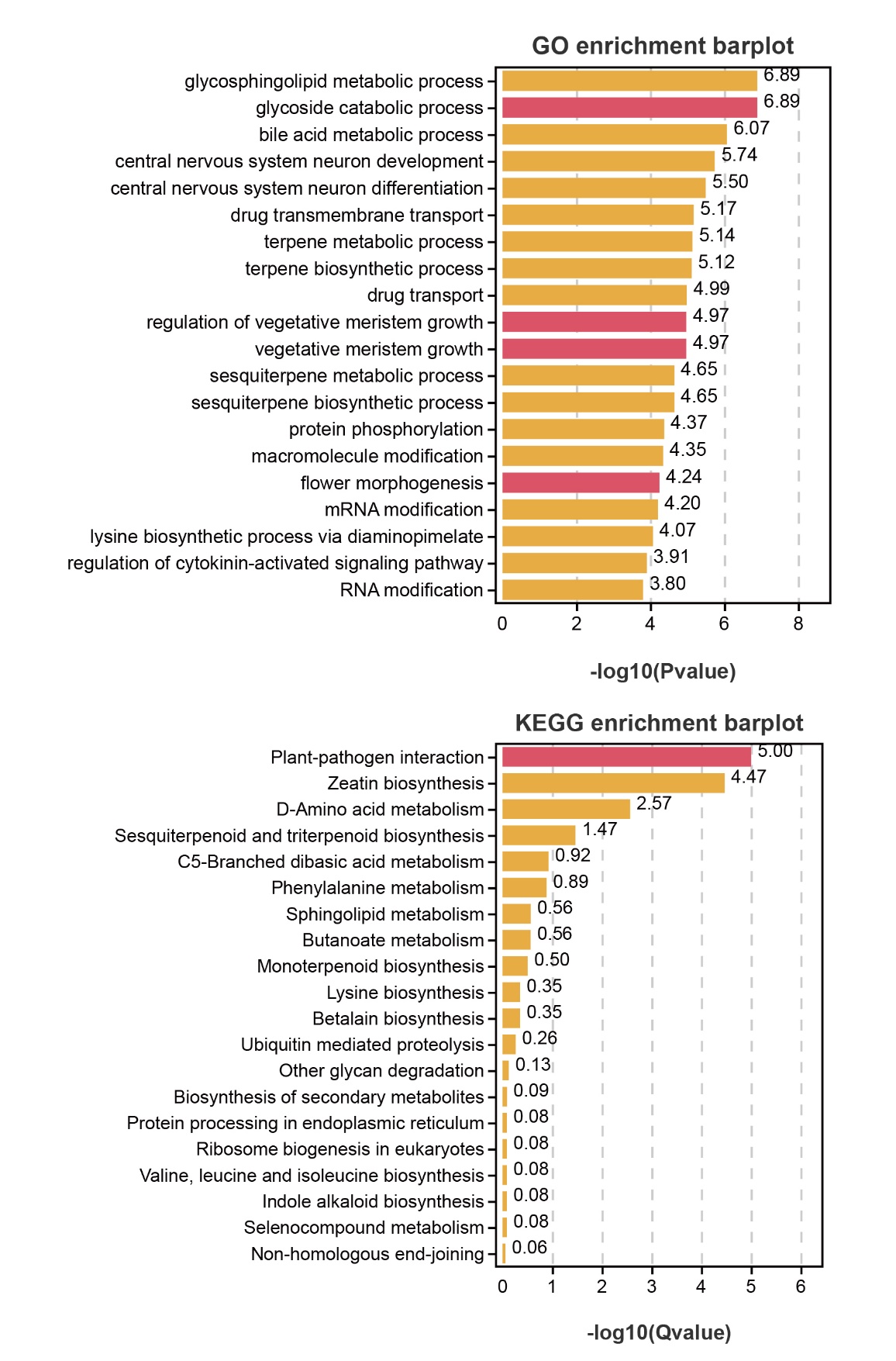
** **Figure S22. Gene function enrichment of longan genes in duplication (DUP) regions of longan and structure variations analysis with lychee.** The top 20 GO terms of biological processes (BP) and pathways of KEGG were exhibited.

**
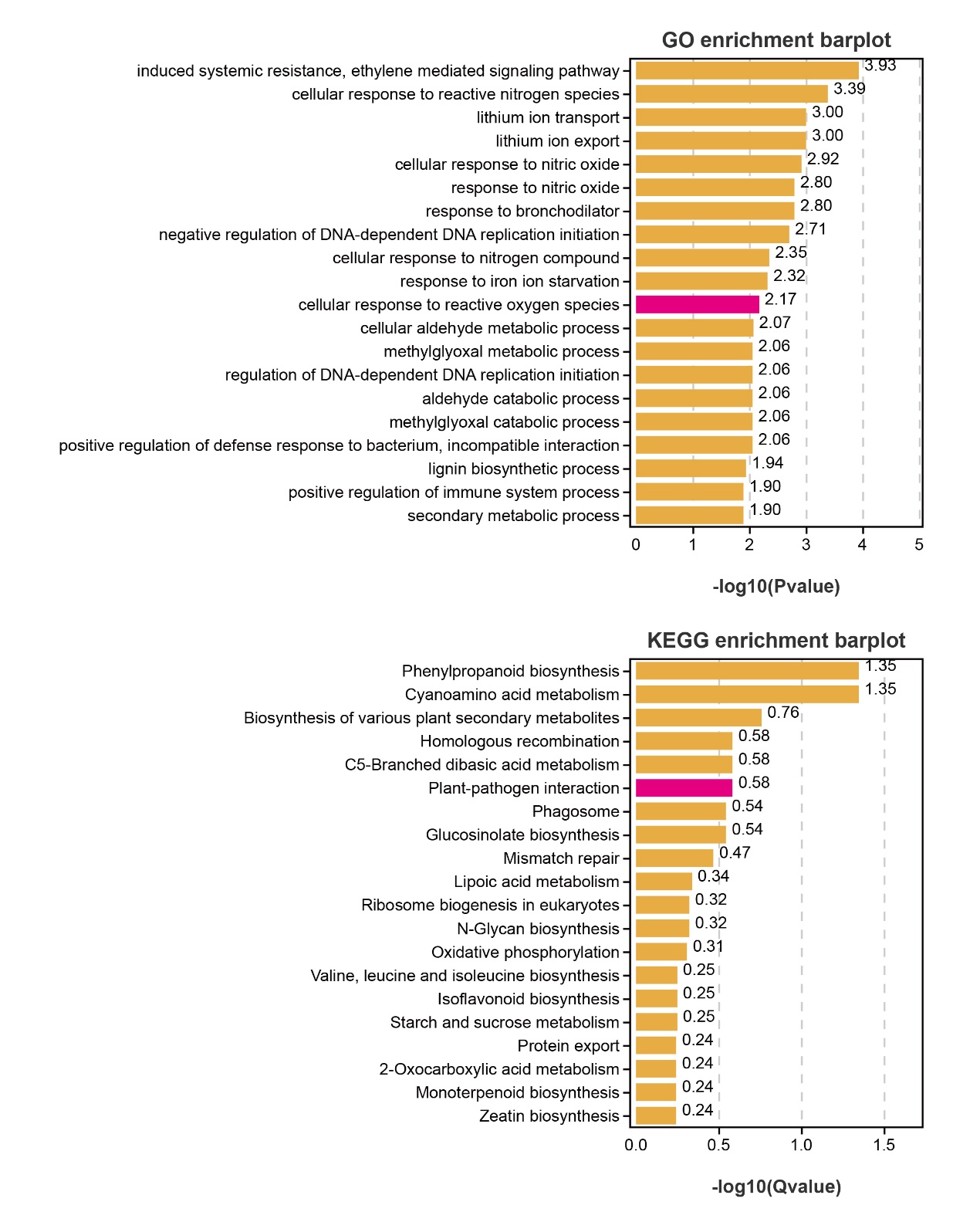
**

**Figure S23. Gene function enrichment of longan genes in translocation (TRANS) regions and structural variation analysis with lychee.** The top 20 GO terms for biological processes (BP) and KEGG pathways are displayed.


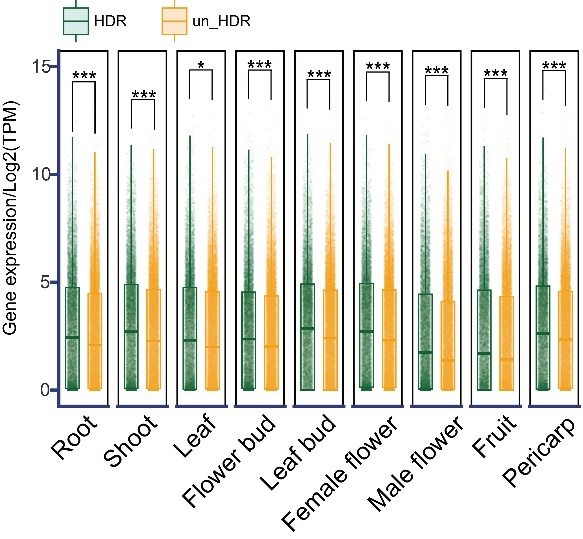


**Figure S24. Expression comparison of longan genes in HDR and other regions of structural variation between longan and lychee.** Significance was analyzed using a *t*-test, with *, **, *** representing *P* < 0.05, *P* < 0.01, and *P* < 0.001, respectively.

**
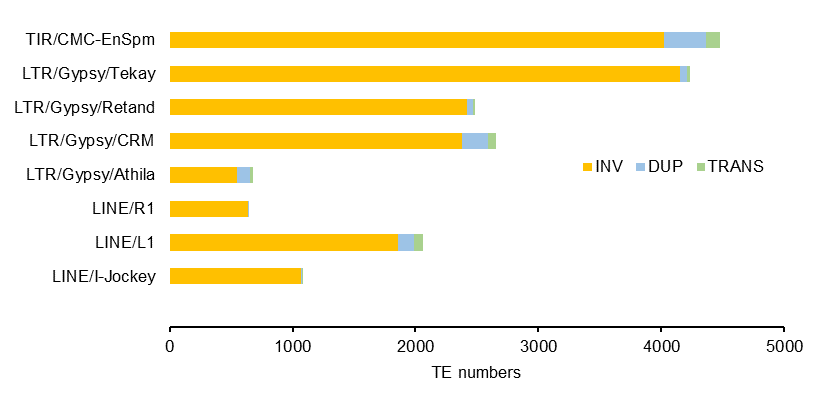
**

**Figure S25. Abundance of transposable elements (TEs) in inverted regions of the longan genome.** DUP, Duplicated region; INV, Inverted region; TRANS, Translocated region.

**
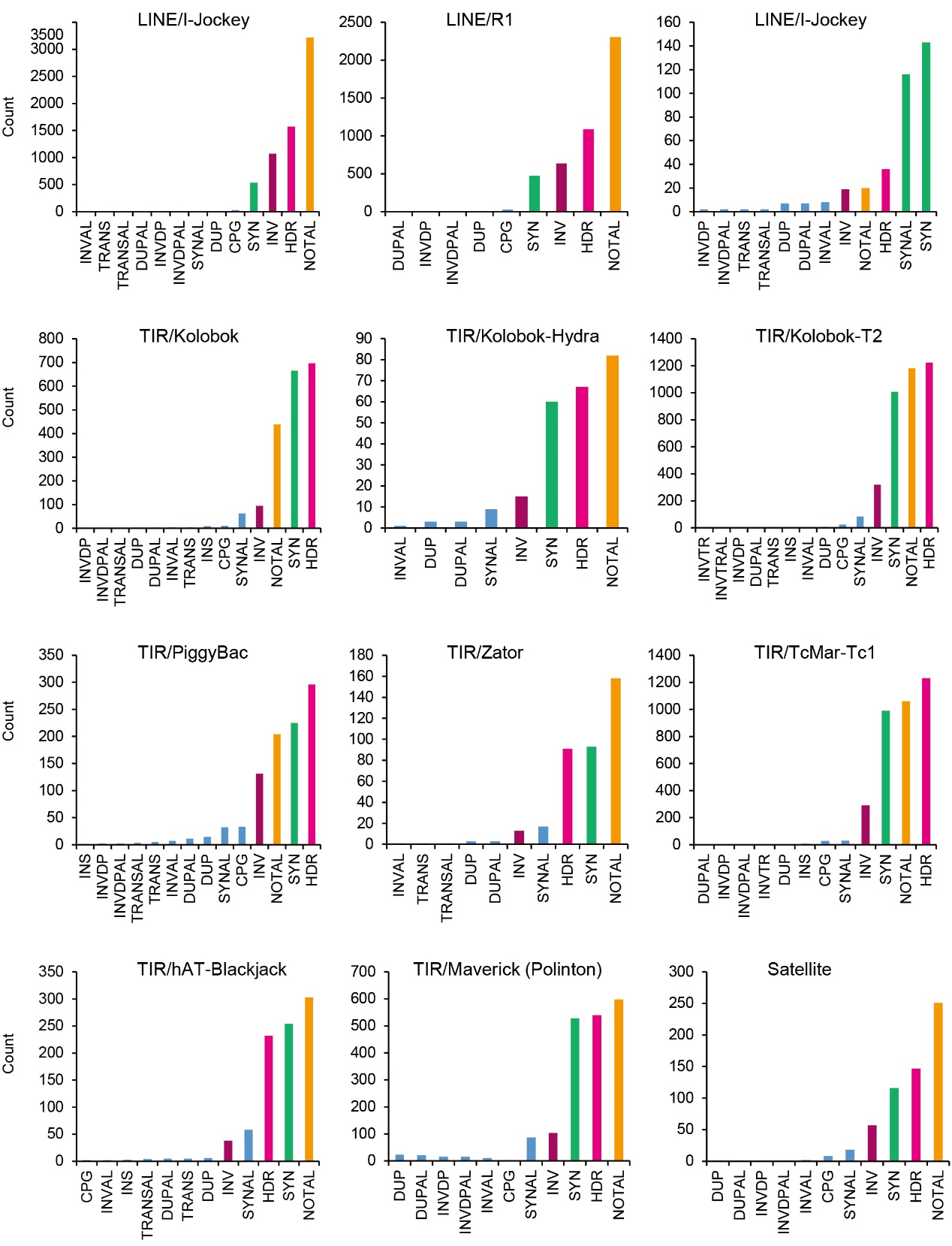
**

**Figure S26. Abundant transposable element (TE) counts in chromosomal structural variation regions of longan and lychee.** CPG, Copy gain in longan genome; CPL, Copy loss in longan genome; DUP, Duplicated region; DUPAL, Alignment in duplicated region; HDR, Highly diverged regions; INS, Insertion in longan genome; INV, Inverted region; INVAL, Alignment in inverted region; INVDP, Inverted duplicated region; INVDPAL, Alignment in inverted duplicated region; INVTR, Inverted translocated region; INVTRAL, Alignment in inverted translocated region; NOTAL, Un-aligned region; TDM, Tandem repeat; TRANS, Translocated region; TRANSAL, Alignment in translocated region; SYN, Syntenic region; SYNAL, Alignment in syntenic region.
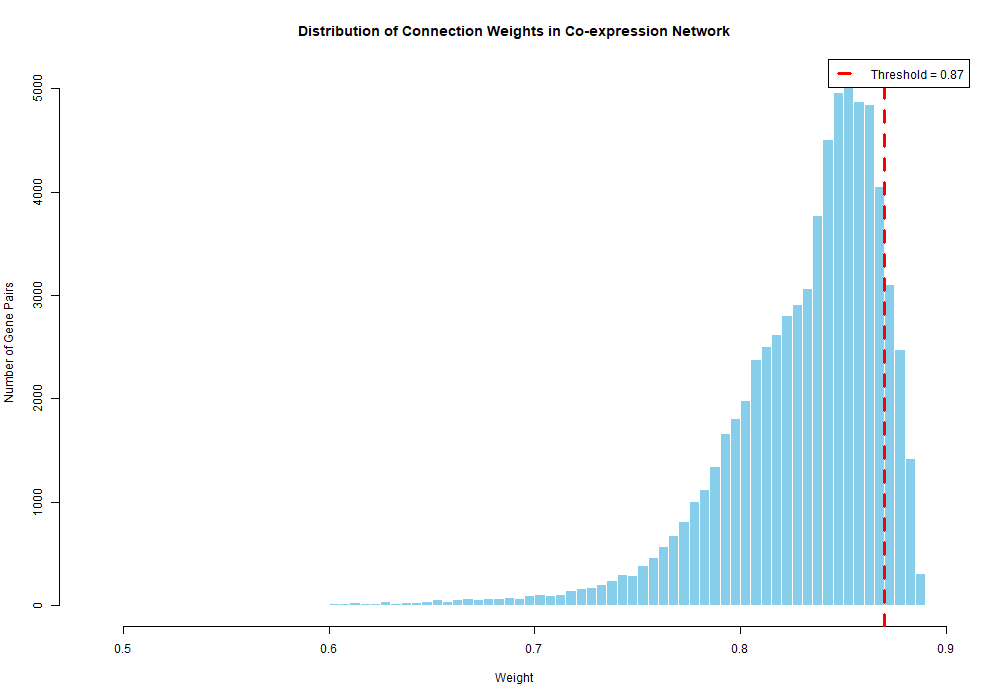
**Figure S27. Distribution of connection weights among co-expressed gene pairs derived from overlapping up-regulated genes in five longan cultivars under natural flowering conditions.** The red dashed line indicates the threshold of 0.87, corresponding to the top 10% of significant co-expression relationships.

**
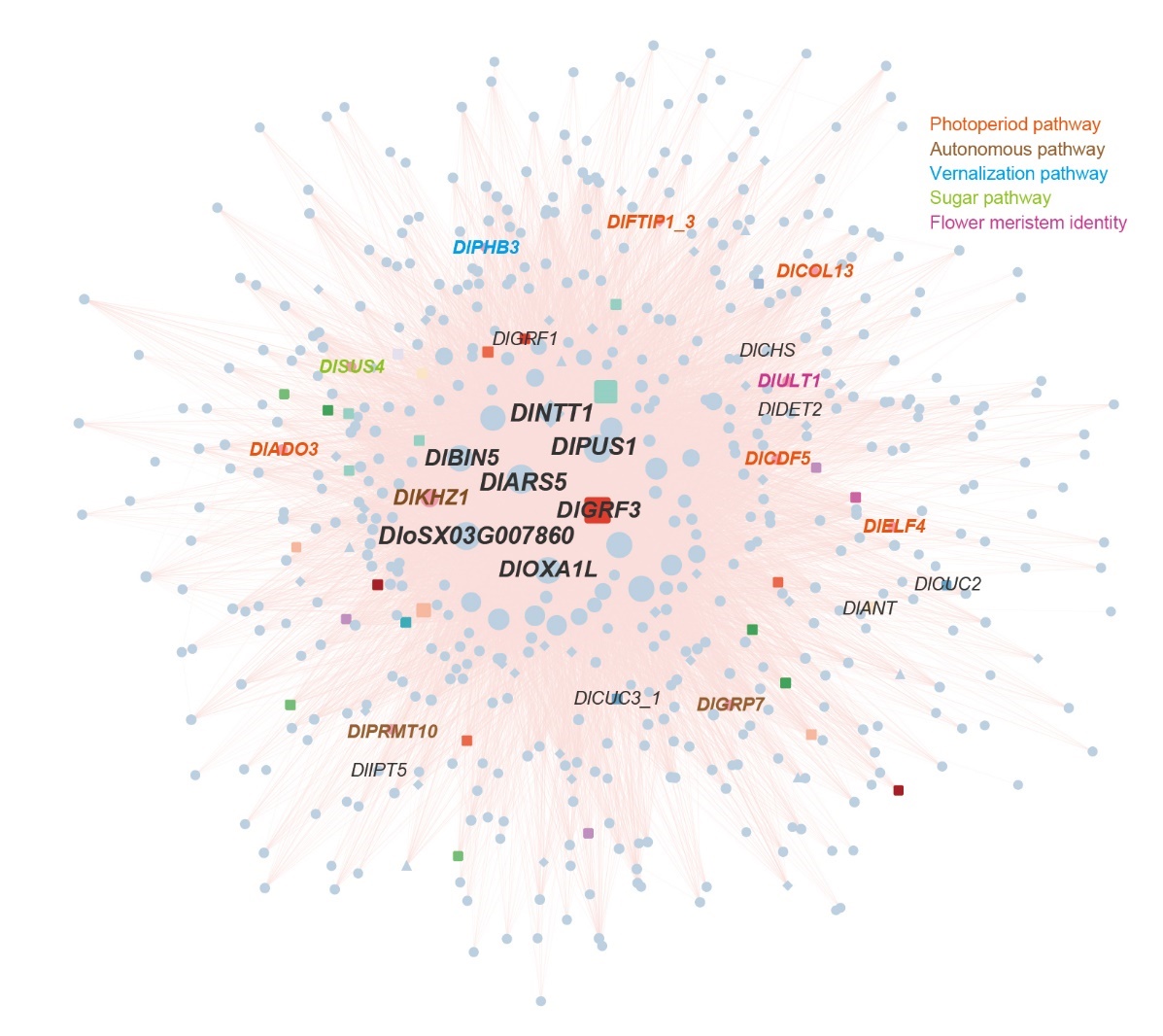
**

**Figure S28. Co-expression network of overlapping up-regulated genes in five** **cultivars under natural flowering conditions.** Weight > 0.87, 557 genes. The central genes, shown in larger font, represent hubs in the co-expression network. Other colored genes were associated with specific flowering pathways, as detailed in the figure legend. Square-shaped nodes indicated transcription factors from various families, triangular nodes represented protein kinases, and diamond-shaped nodes corresponded to plant hormone-related genes.

**
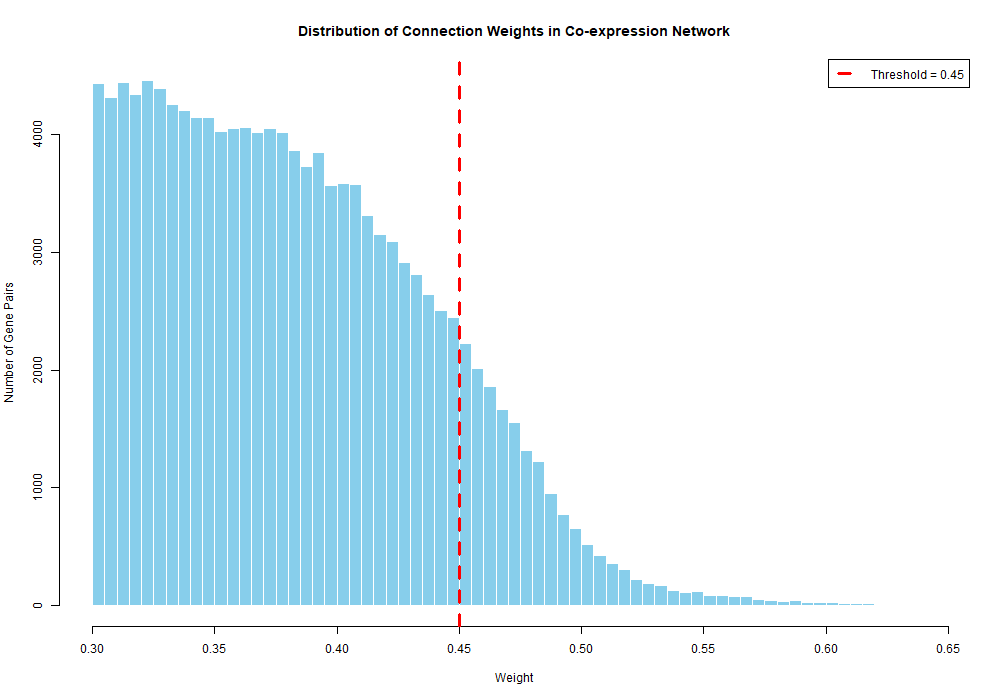
**

**Figure S29. Distribution of connection weights among co-expressed gene pairs derived from overlapping down-regulated genes in five longan cultivars under natural flowering conditions.** The red dashed line indicates the threshold of 0.45, corresponding to the top 10% of significant co-expression relationships.

**
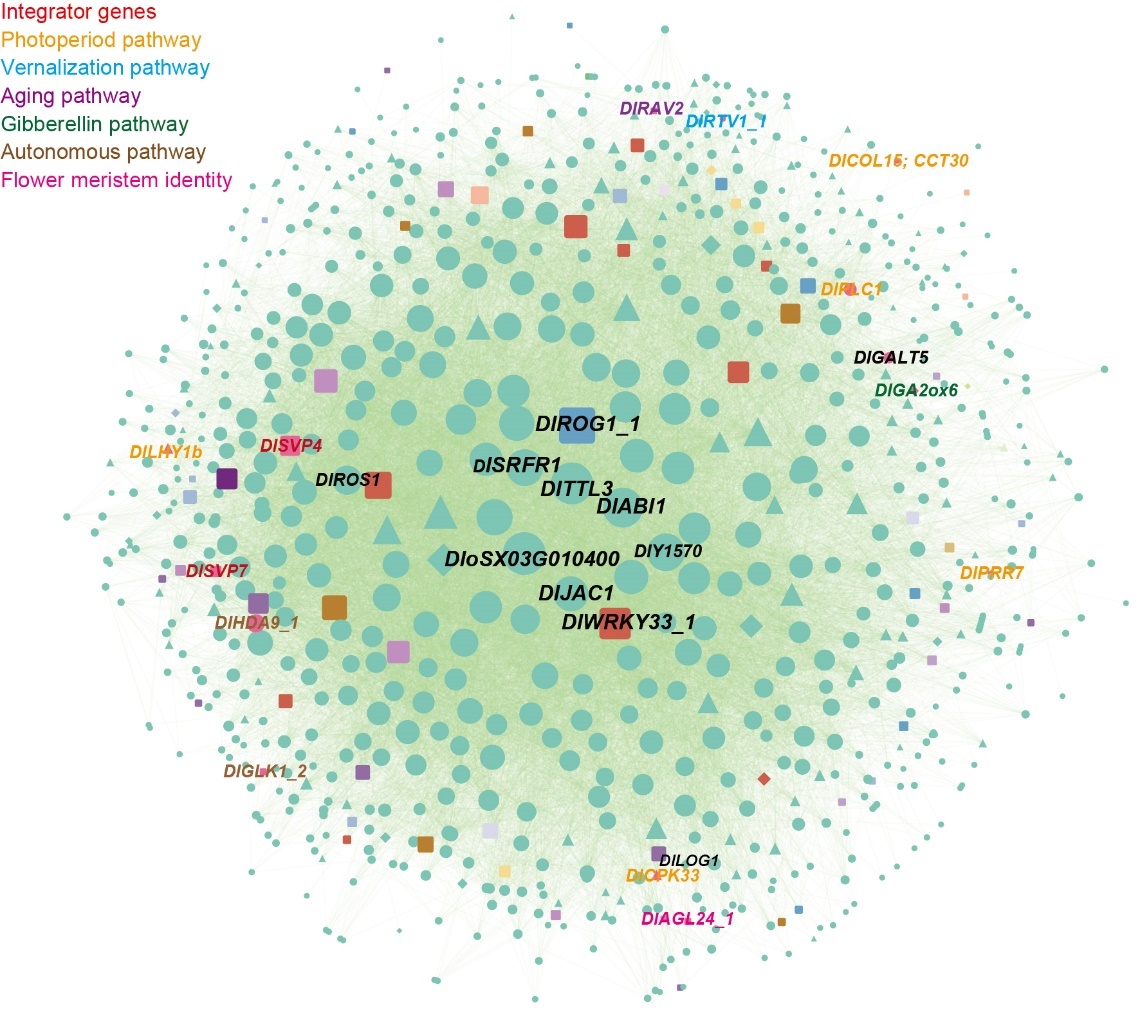
**

**Figure S30. Co-expression network of overlapping down-regulated genes in five cultivars under natural flowering conditions.** Weight > 0.45, 821 genes. The central genes, shown in larger font, represent hubs in the co-expression network. Other colored genes were associated with specific flowering pathways, as detailed in the figure legend. Square-shaped nodes indicated transcription factors from various families, triangular nodes represented protein kinases, and diamond-shaped nodes corresponded to plant hormone-related genes.

**
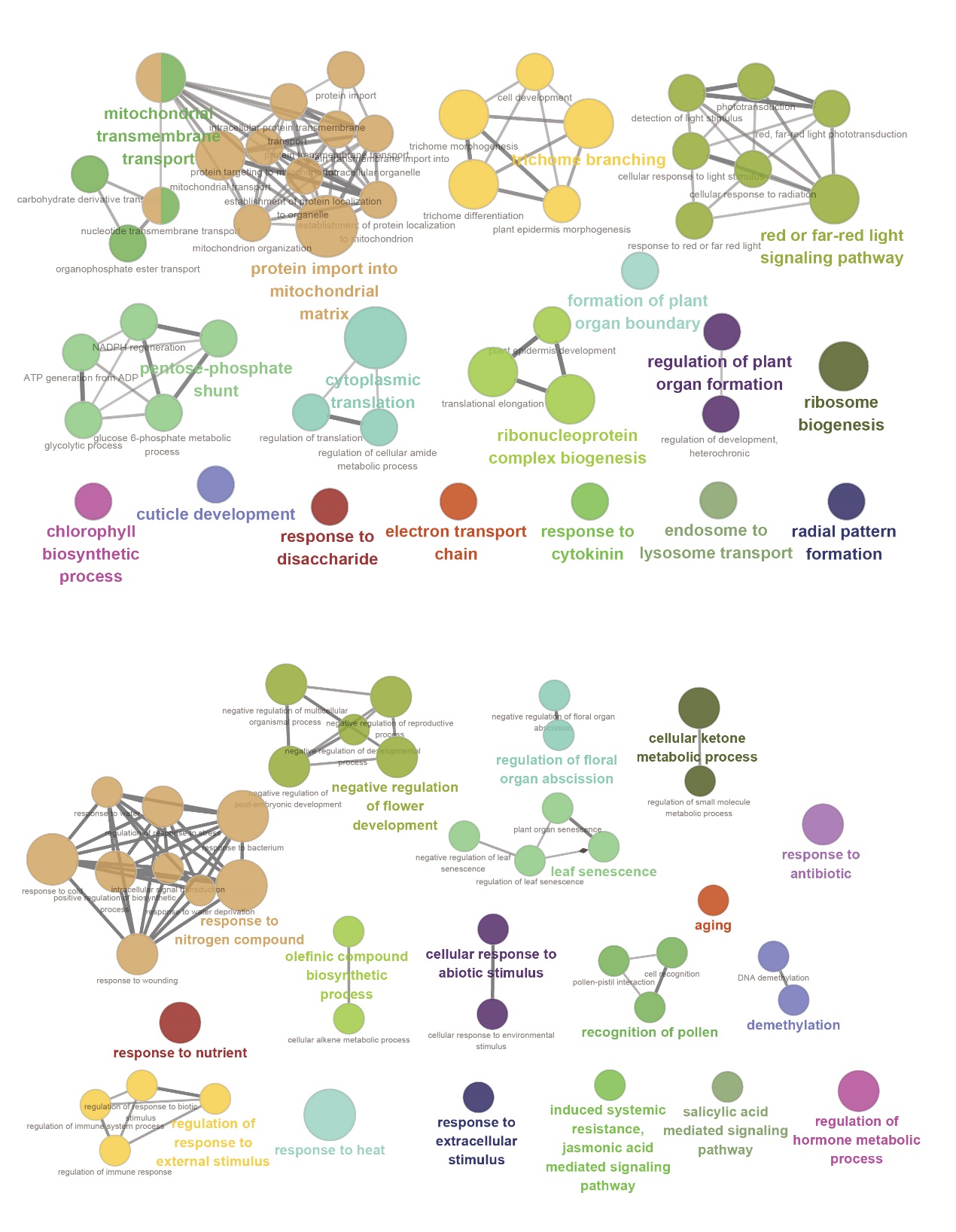
**

**Figure S31. GO enrichment analysis of overlapped up-regulated genes in flower bud tissues of five cultivars.** DEGs were filtered based on the criteria of |Log_2_FC| > 1 and *FDR* < 0.05.

**
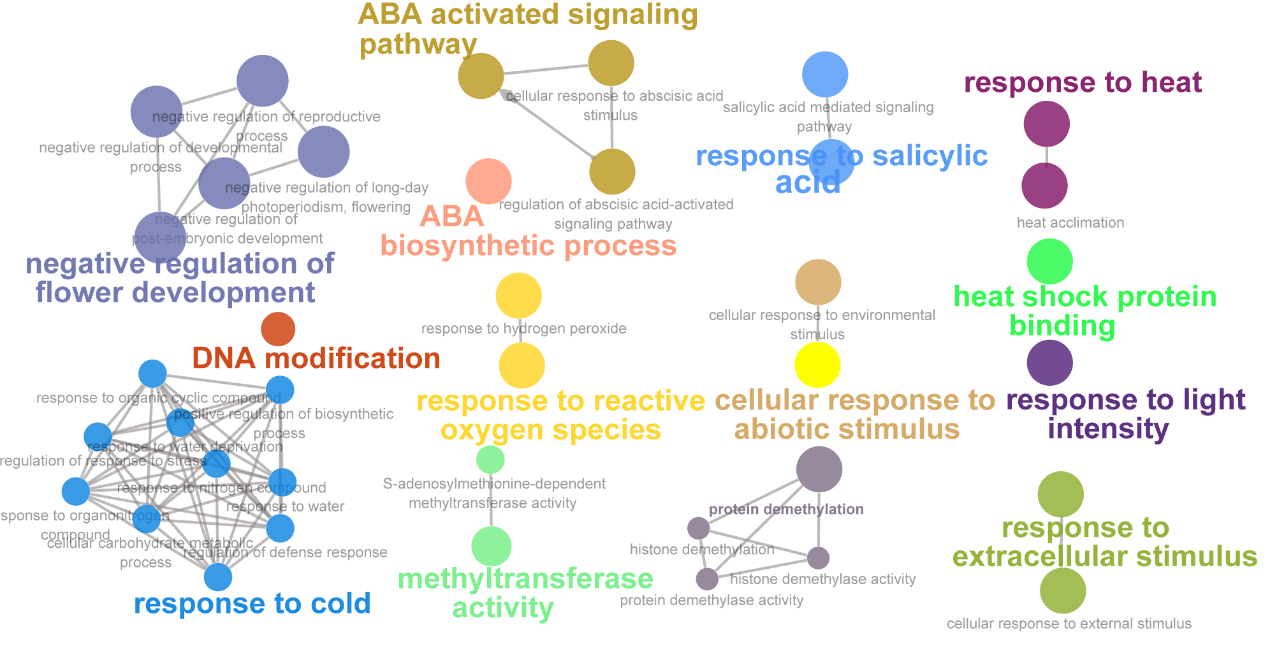
** **Figure S32.** **GO enrichment analysis of uniquely down-regulated genes in flower bud tissues.** DEGs were filtered by the standard of |Log_2_FC| > 2, *FDR* < 0.05.

**
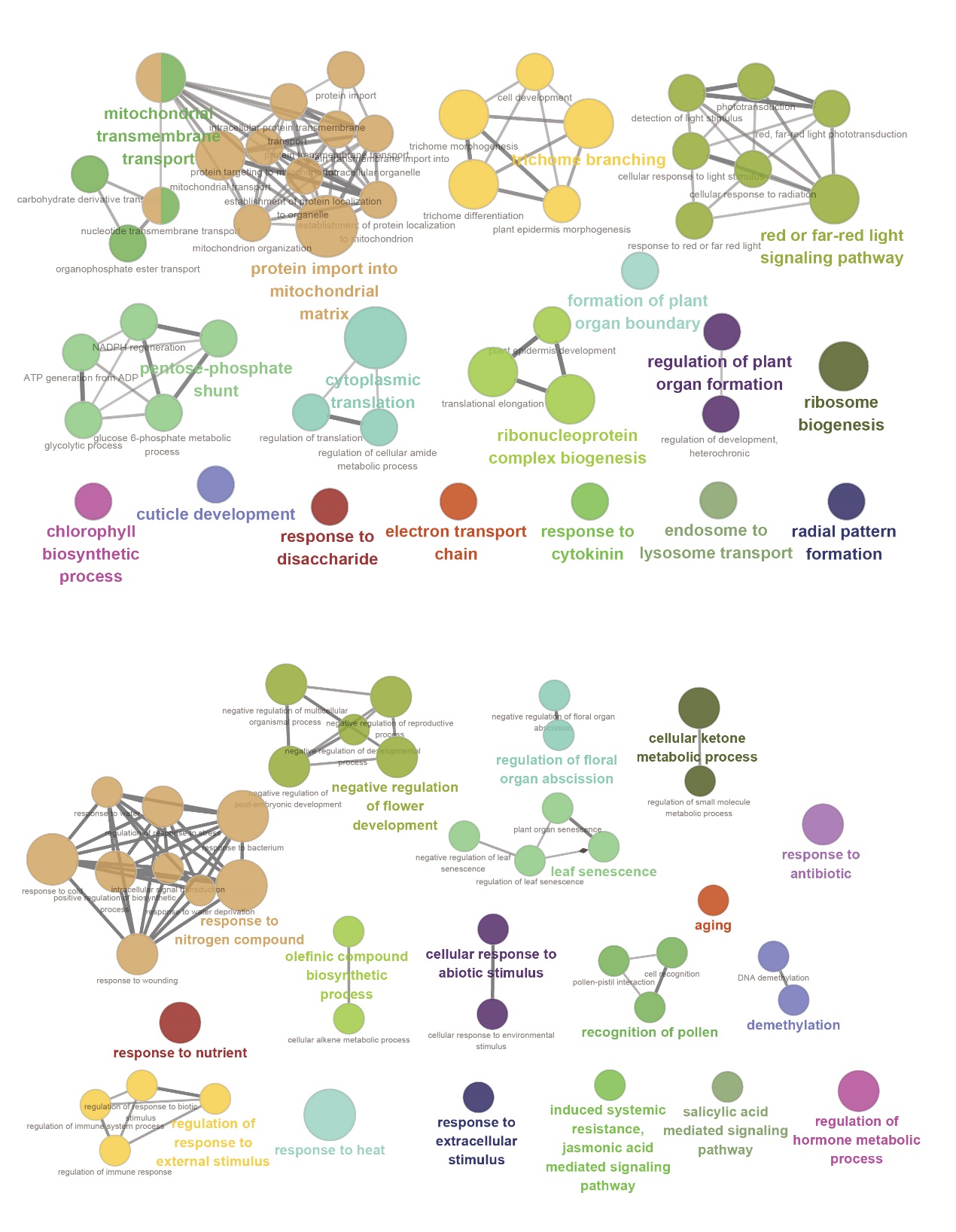
**

**Figure S33.** **GO enrichment analysis of overlapped down-regulated genes in flower bud tissues of five cultivars.** DEGs were filtered by the standard of |Log_2_FC|>1, *FDR* < 0.05.
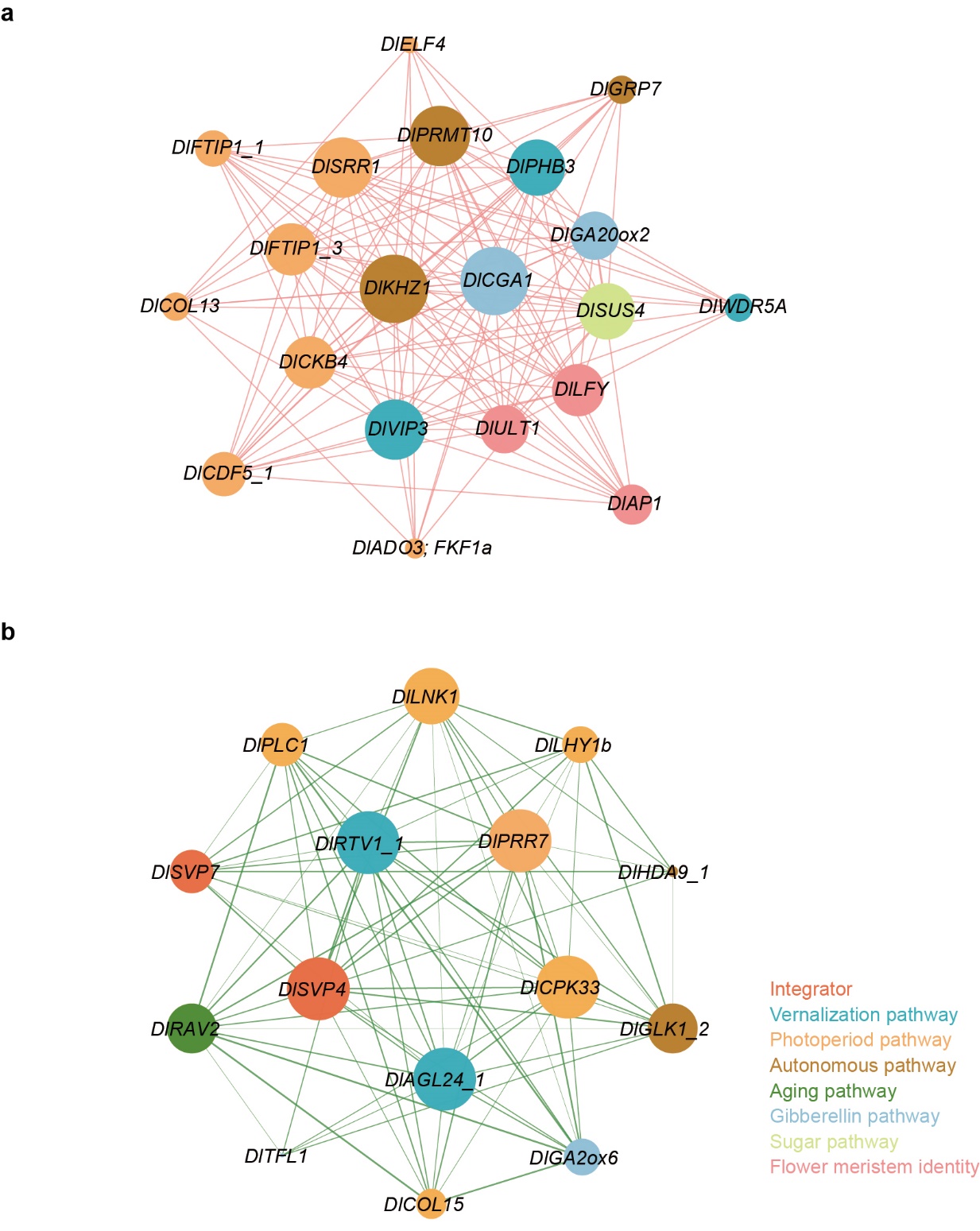


**Figure S34.** **Co-expression networks of overlapping up- and down-regulated flowering genes in flower bud tissues of five cultivars. a.** Overlapping up-regulated flowering genes. **b.** Overlapping down-regulated flowering genes. The networks were constructed based on Pearson correlation coefficients (≥ 0.7) of gene expression levels in flower bud and leaf bud tissues.

**
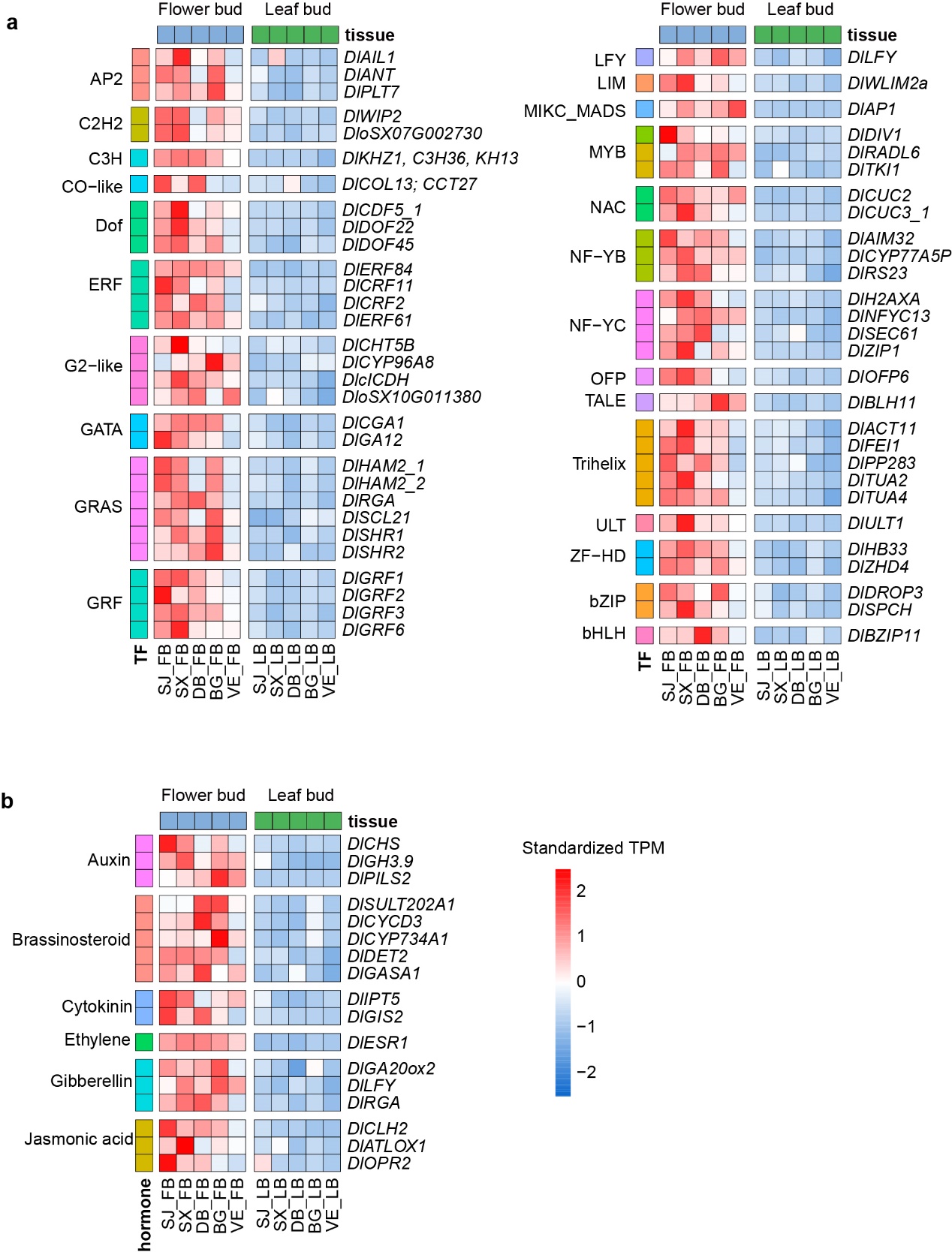
**

**Figure S35. Up-regulated transcription factors (TFs) and hormone-related genes in the flower bud tissues of all five cultivars.** a. TFs. b. Hormone-related genes. DEGs were filtered by the standard of |Log_2_FC| > 1, *FDR* < 0.05.


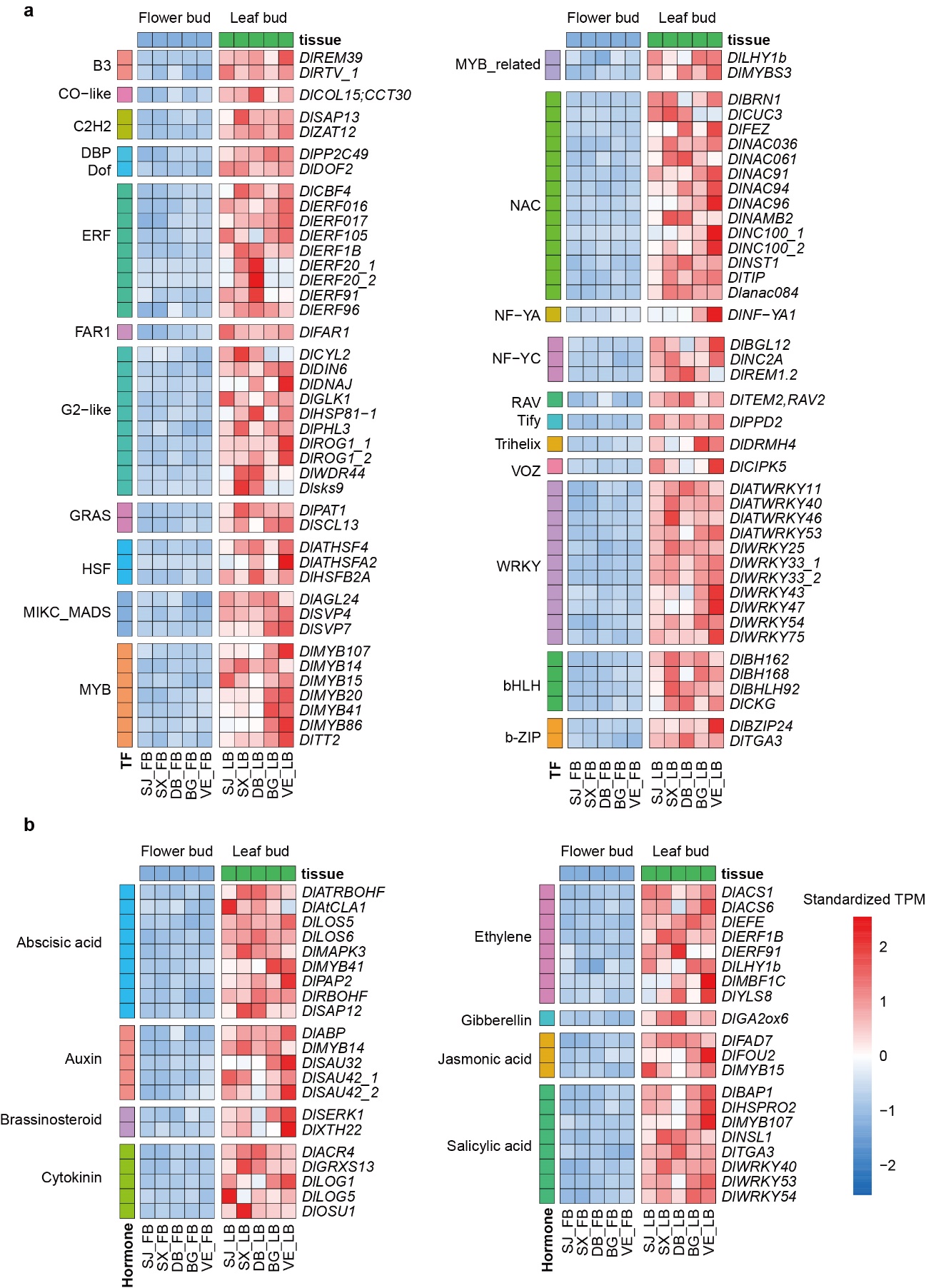


**Figure S36. Down-regulated transcription factors (TFs) and hormone-related genes in the flower bud tissues of all five cultivars. a.** TFs. **b.** Hormone-related genes. DEGs were filtered by the standard of |Log_2_FC| > 1, *FDR* < 0.05.


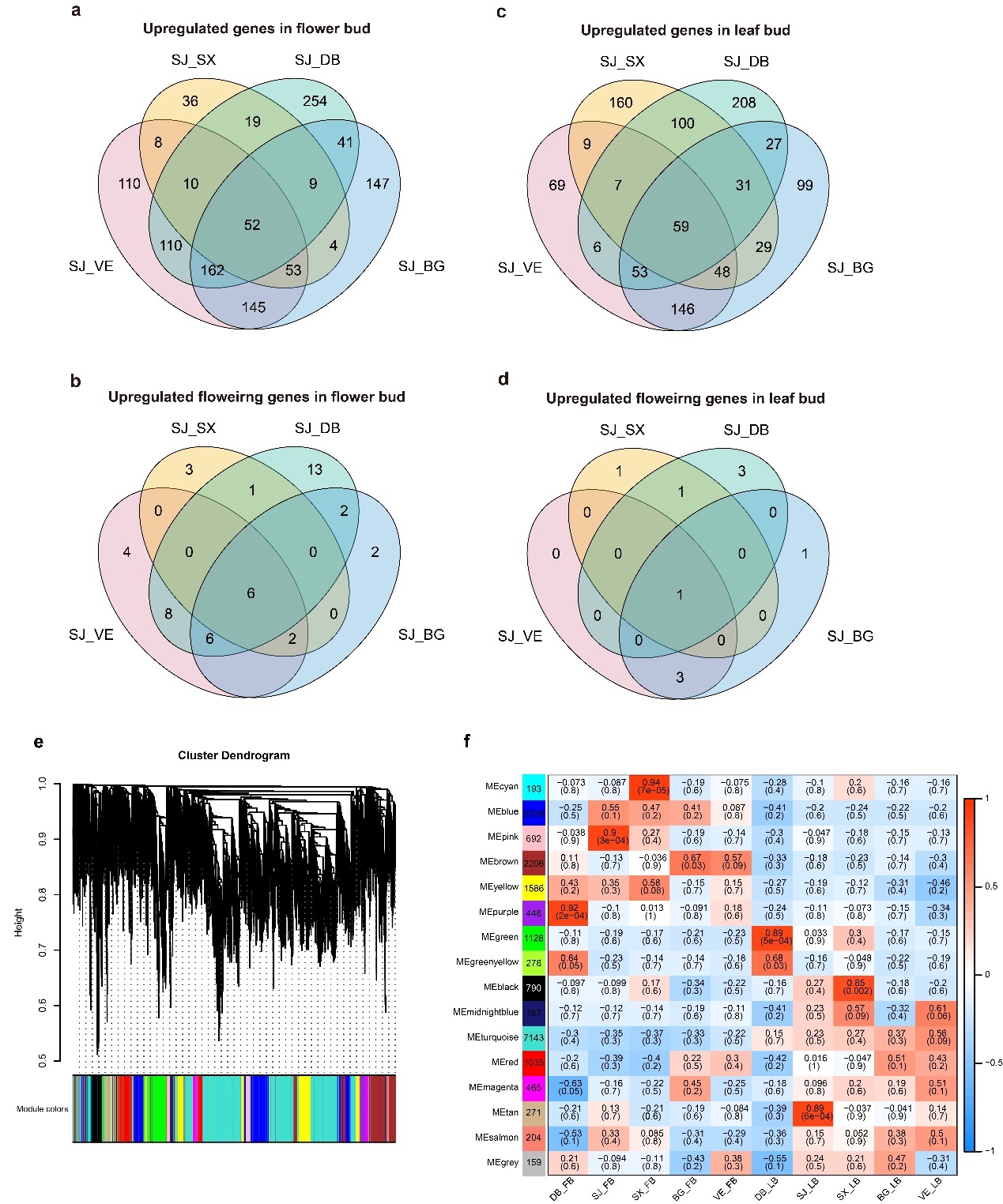


**Figure S37. Specifically expressed DEGs in the flower bud of ‘Sijimi’ compared with other five cultivars. a.** Venn diagram of up-regulated and down-regulated DEGs in the flower buds of **‘Sijimi’** compared with the other four cultivars. **b.** Cluster dendrogram of expressed genes in all the flower and leaf bud tissues. **c.** Correlation heat map of weighted gene co-expression network analysis (WGCNA) modules and tissues.


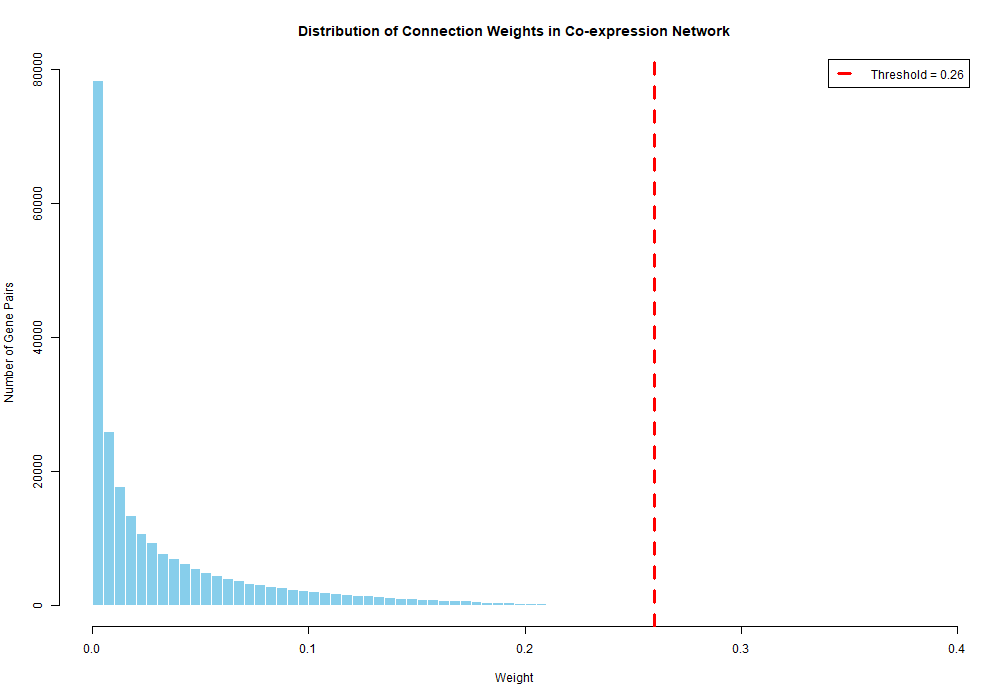
**Figure S38. Distribution of connection weights among co-expressed gene pairs in the ‘Sijimi’ flower bud-specific MEpink.** The red dashed line indicates the threshold of 0.26, corresponding to the top 0.15% of significant co-expression relationships.


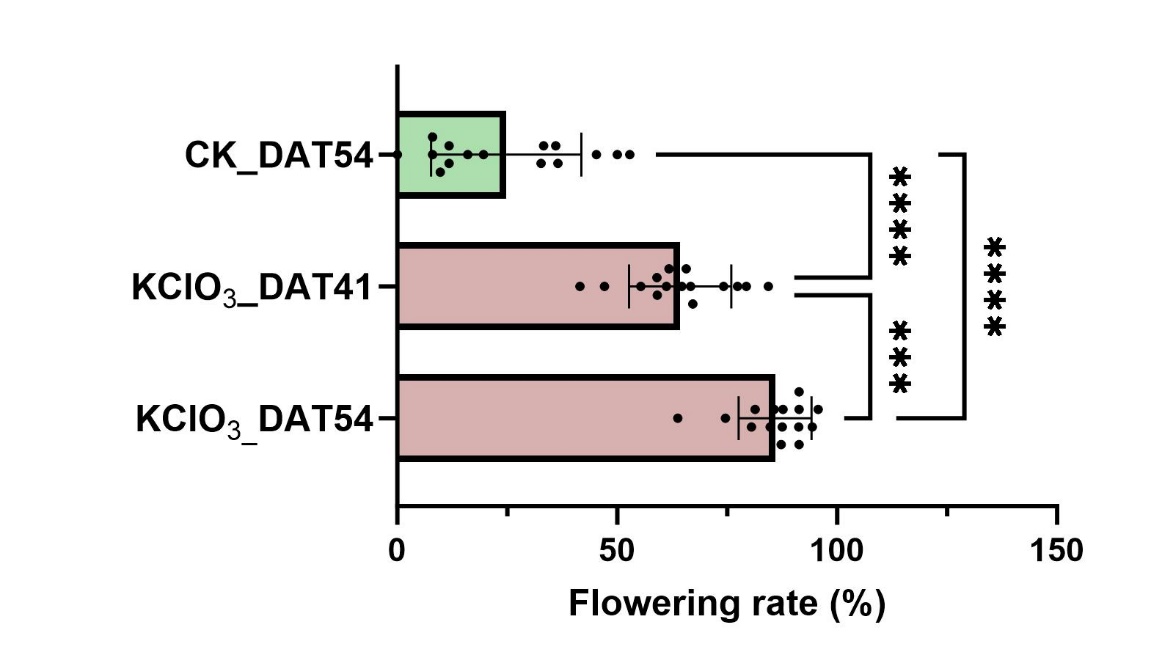


**Figure S39. Comparison of flowering rates between KClO₃ treatment and control (CK) group.** The flowering rate was calculated as the proportion of flower buds that developed into inflorescence primordium relative to the total number of budding buds on newly developed branches of a tree. Statistical analysis was performed using a *t*-test, with **** indicating *P* < 0.0001.


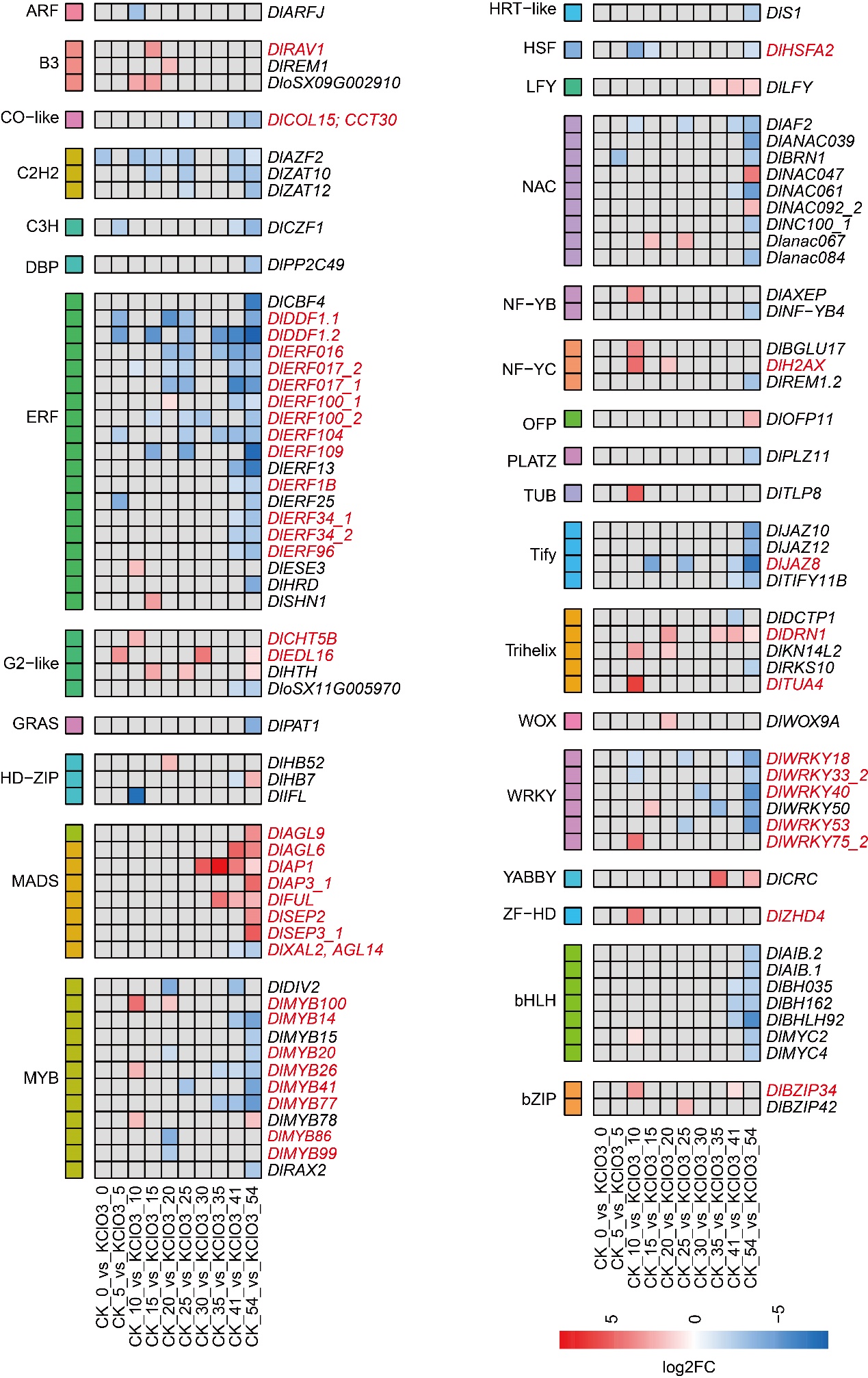


**Figure S40. Heat maps of differentially expressed TFs for CK and KClO_3_ treatment at different stages.** Red squares indicated upregulation after KClO_3_ treatment, while blue squares represented downregulation, and gray squares indicated no significant difference. DEGs were filtered by the criteria of |log_2_(FC)| > 2 and *FDR* < 0.05. Genes highlighted in red showed the most significant differential expression between the two treatments.


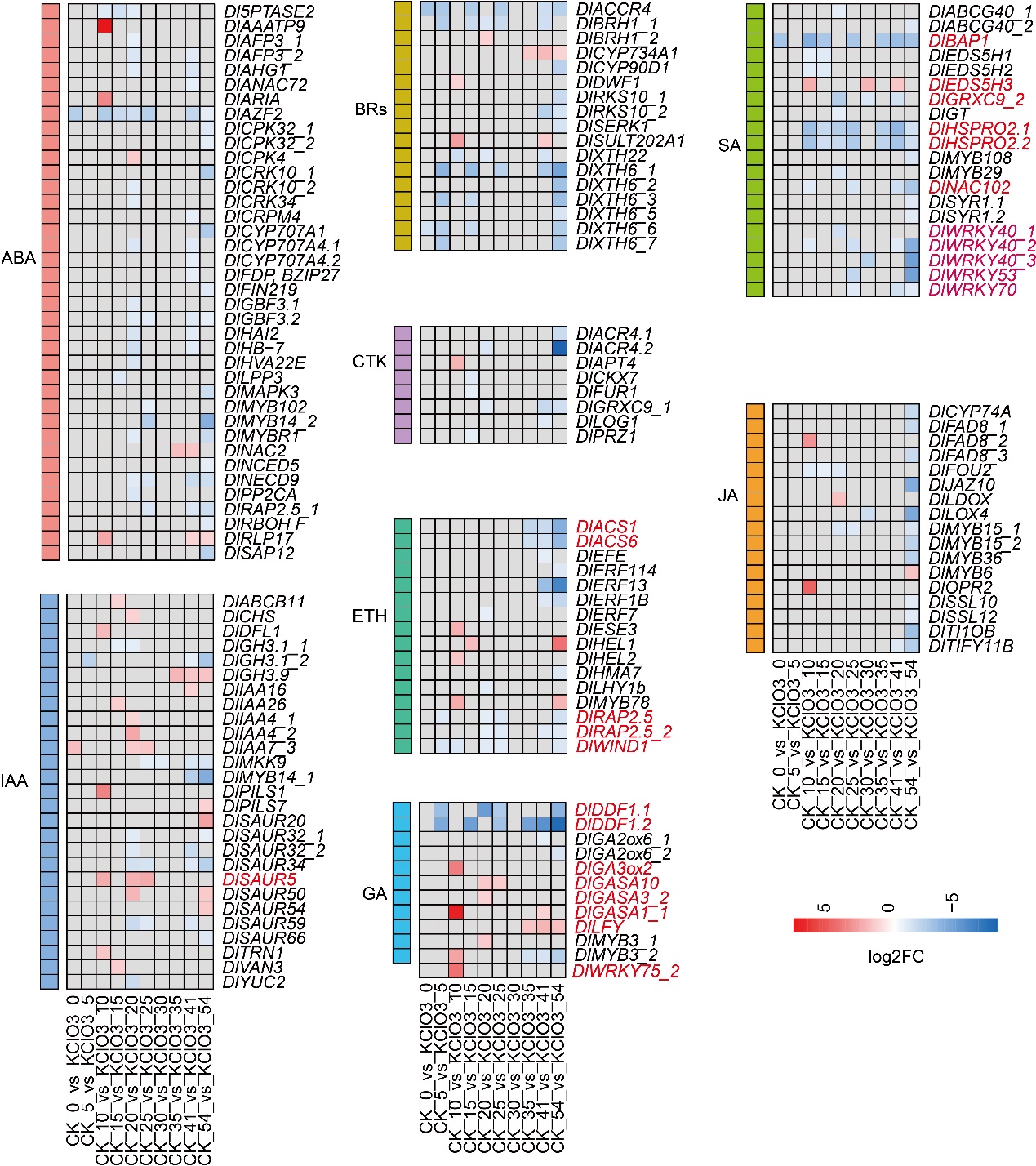


**Figure S41. Heat maps of differently expressed hormone-related genes for CK and KClO_3_ treatments at different stages.** Red squares indicated up-regulation after KClO_3_ treatment, blue squares represented down-regulation, and gray squares indicated no difference. DEGs were filtered by the standard of |log_2_(FC)| > 1 and *FDR* < 0.05. The genes highlighted in red show the most significant differential expression between the two treatments.


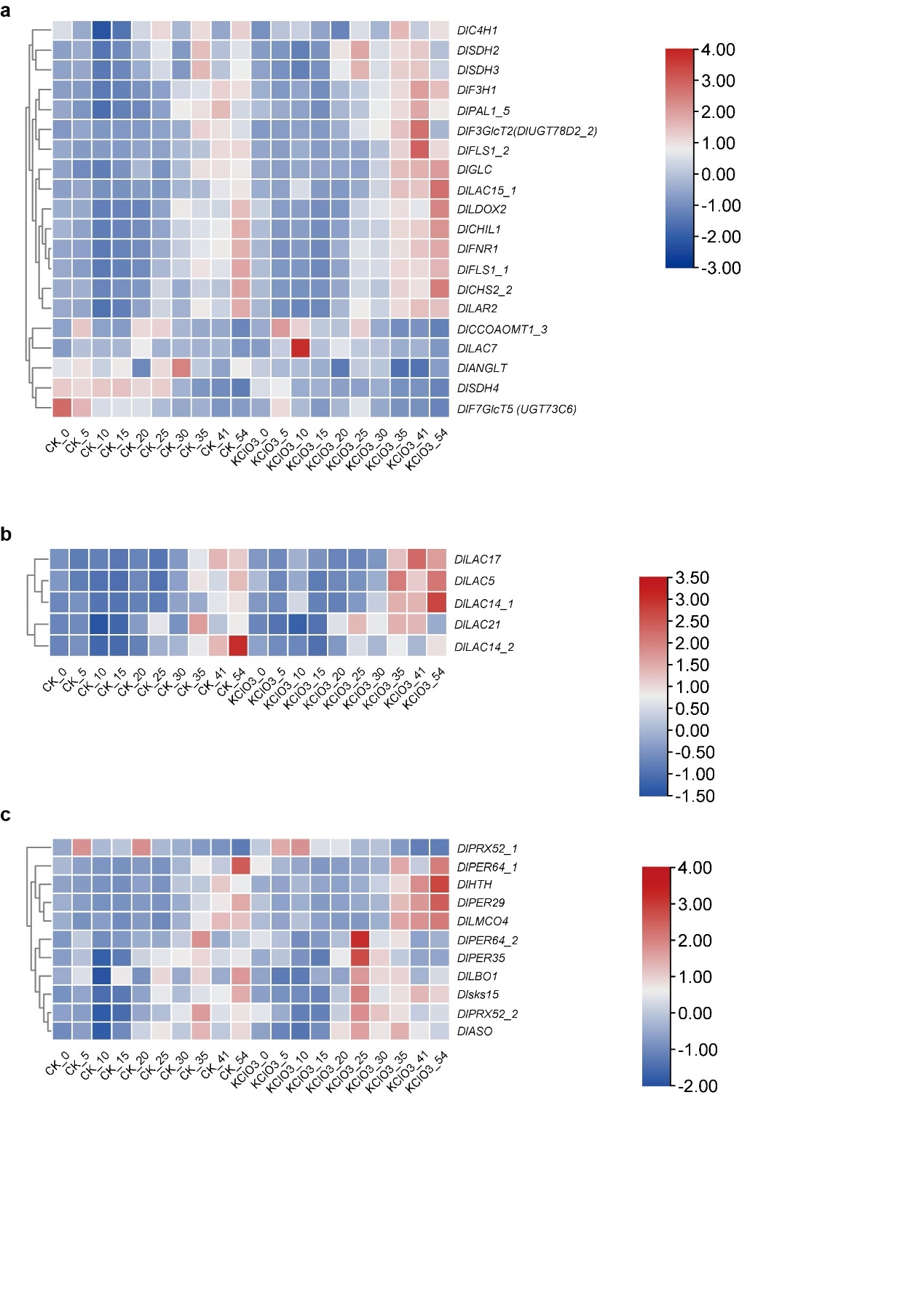


**Figure S42. Heat maps of DEGs related to oxidative stress in KClO_3_-induced flower bud tissues. a.** Flavonoid biosynthesis-related genes; **b.** Redox reaction-related genes; **c.** Lignin degradation and detoxification-related genes.


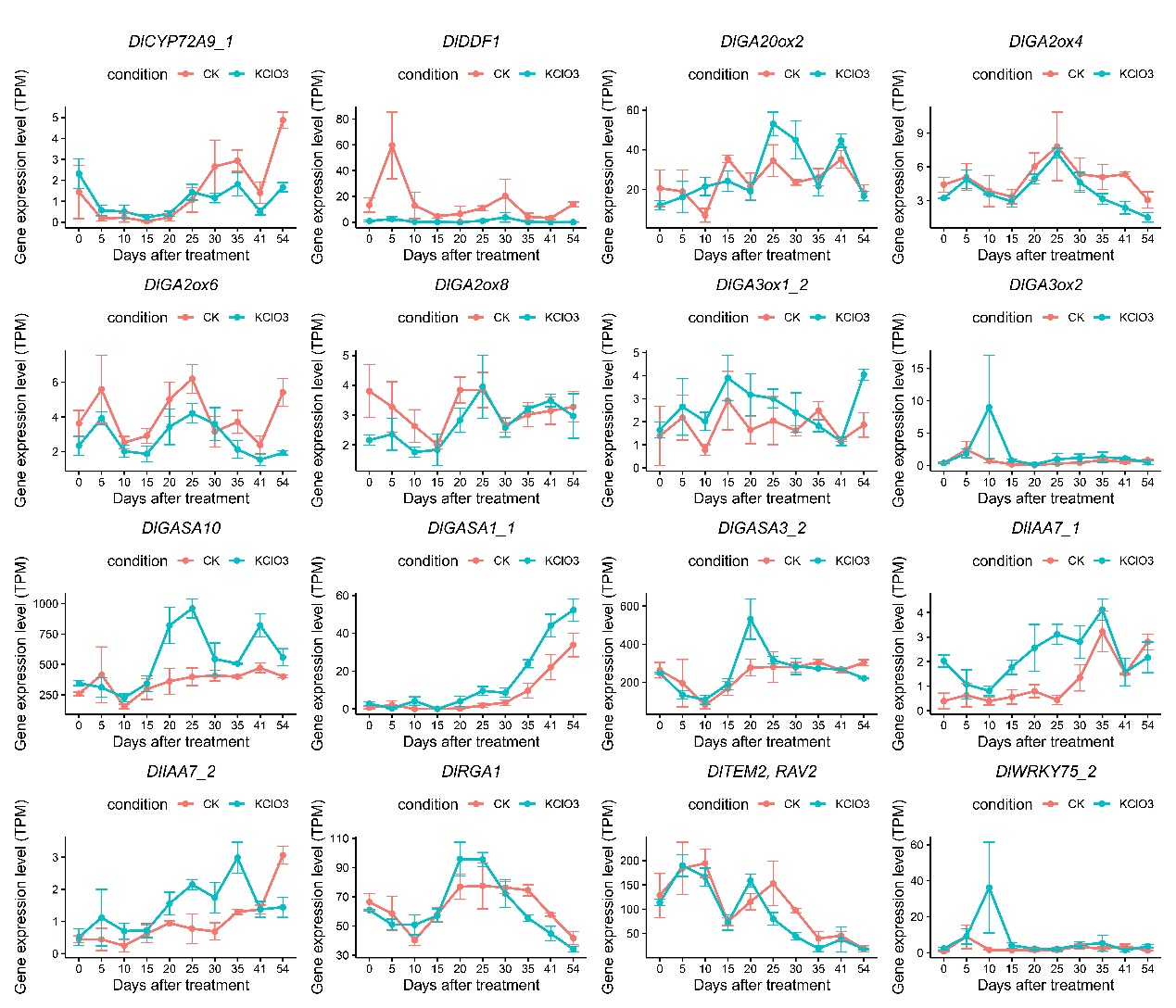


**Figure S43. Expression patterns of gibberellin pathway-related genes under KClO₃ treatment and natural flowering conditions (control group).** The y-axis represents gene expression level, with red indicating the natural flowering control group and green representing KClO₃-treated samples.

**
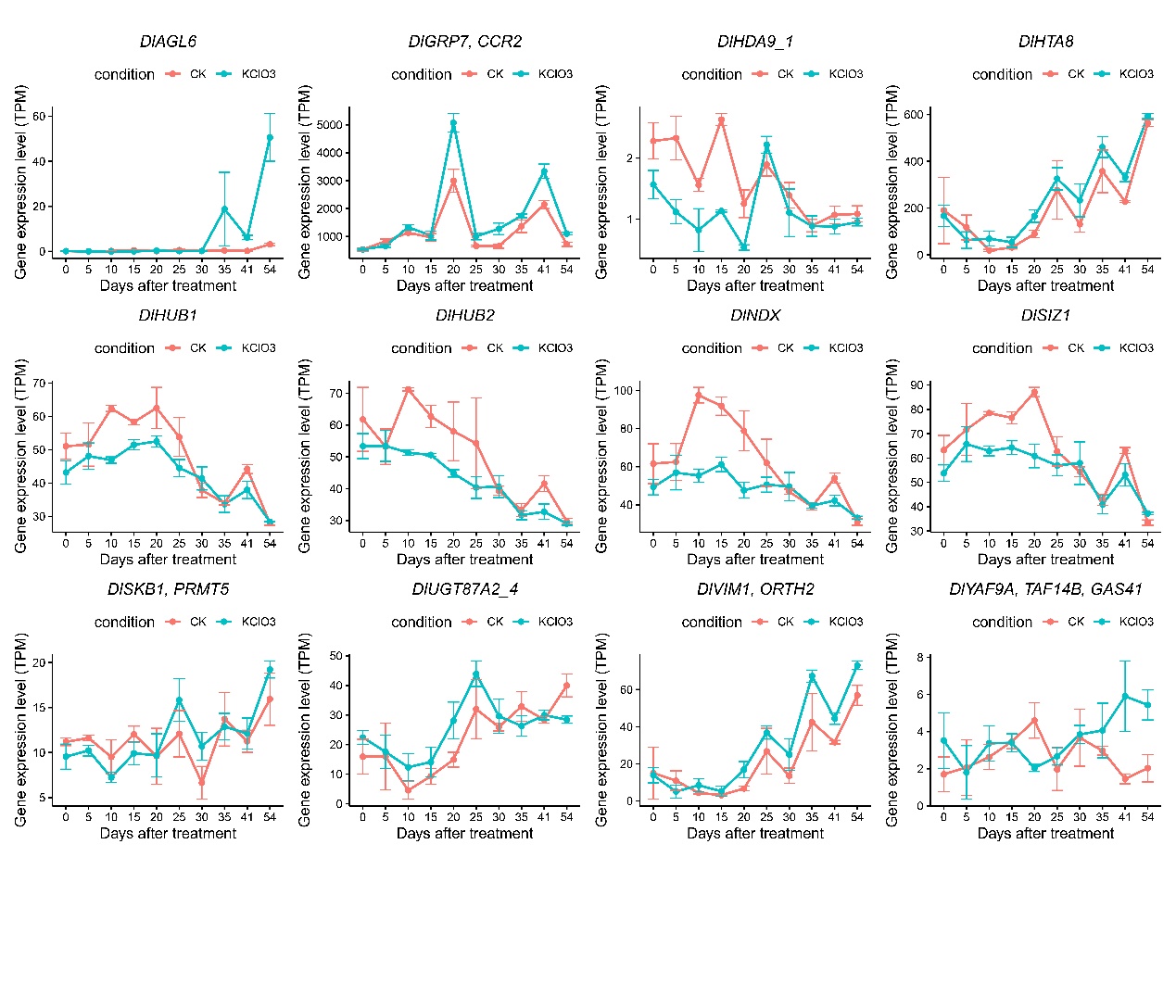
Figure S44. Expression patterns of autonomous pathway-related genes under KClO₃ treatment and natural flowering conditions (control group).** The y-axis represents gene expression level, with red indicating the natural flowering control group and green representing KClO₃-treated samples.

**
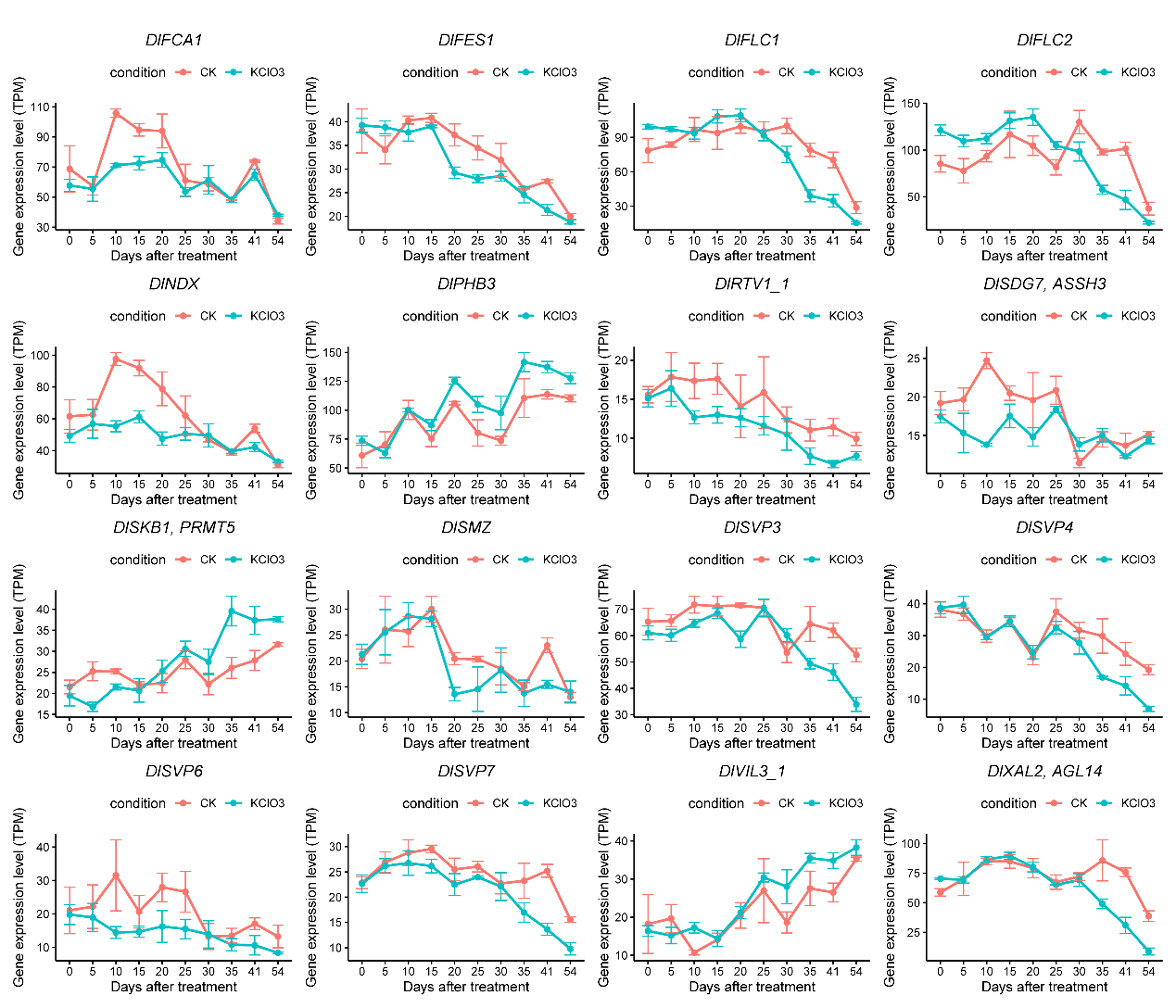
**

**Figure S45. Expression patterns of vernalisation pathway-related genes under KClO₃ treatment and natural flowering conditions (control group).** The y-axis represents gene expression level, with red indicating the natural flowering control group and green representing KClO₃-treated samples.

**
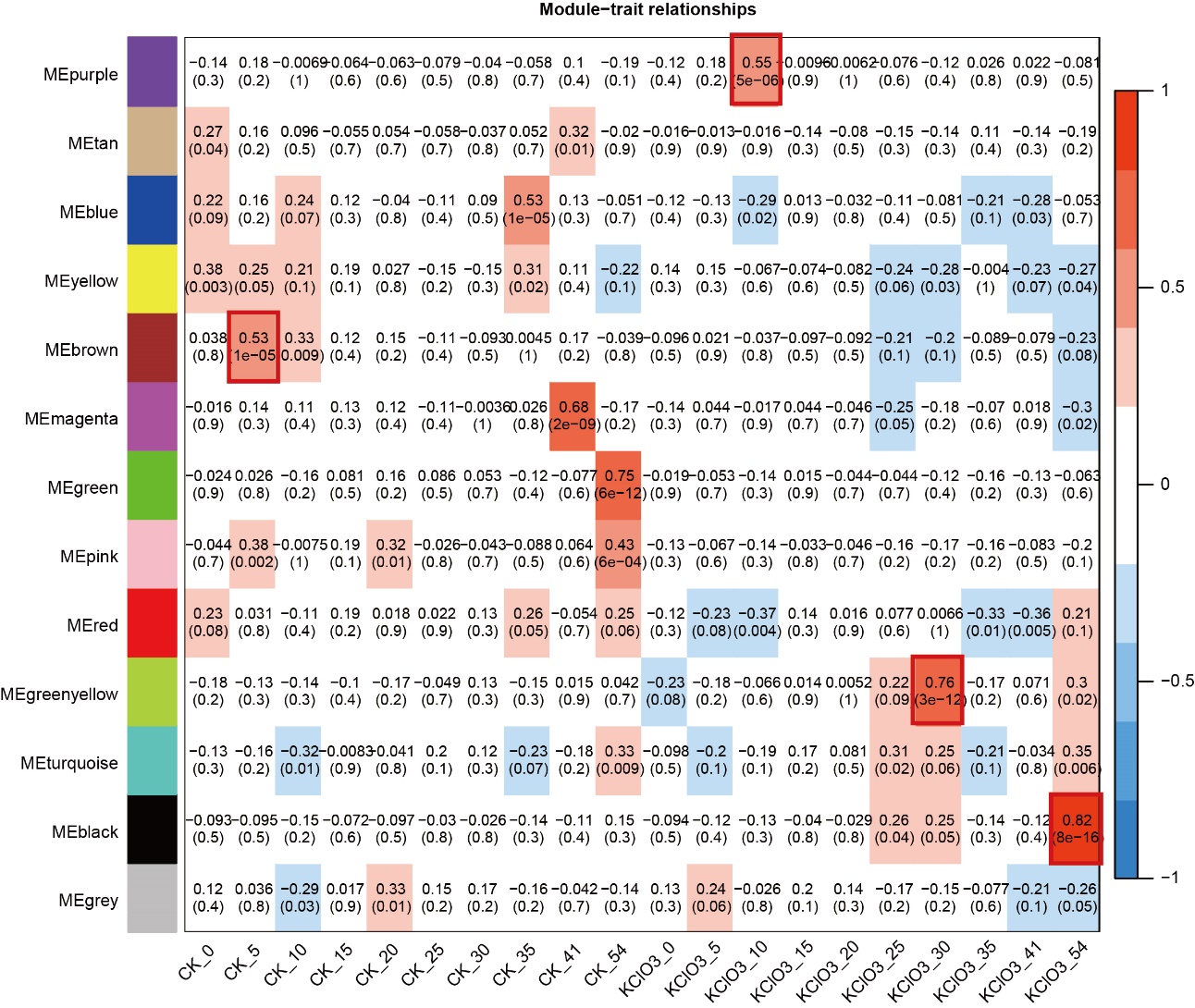
**

**Figure S46. Module-trait relationships showing the correlation between gene expression levels and trait intensity.**

**
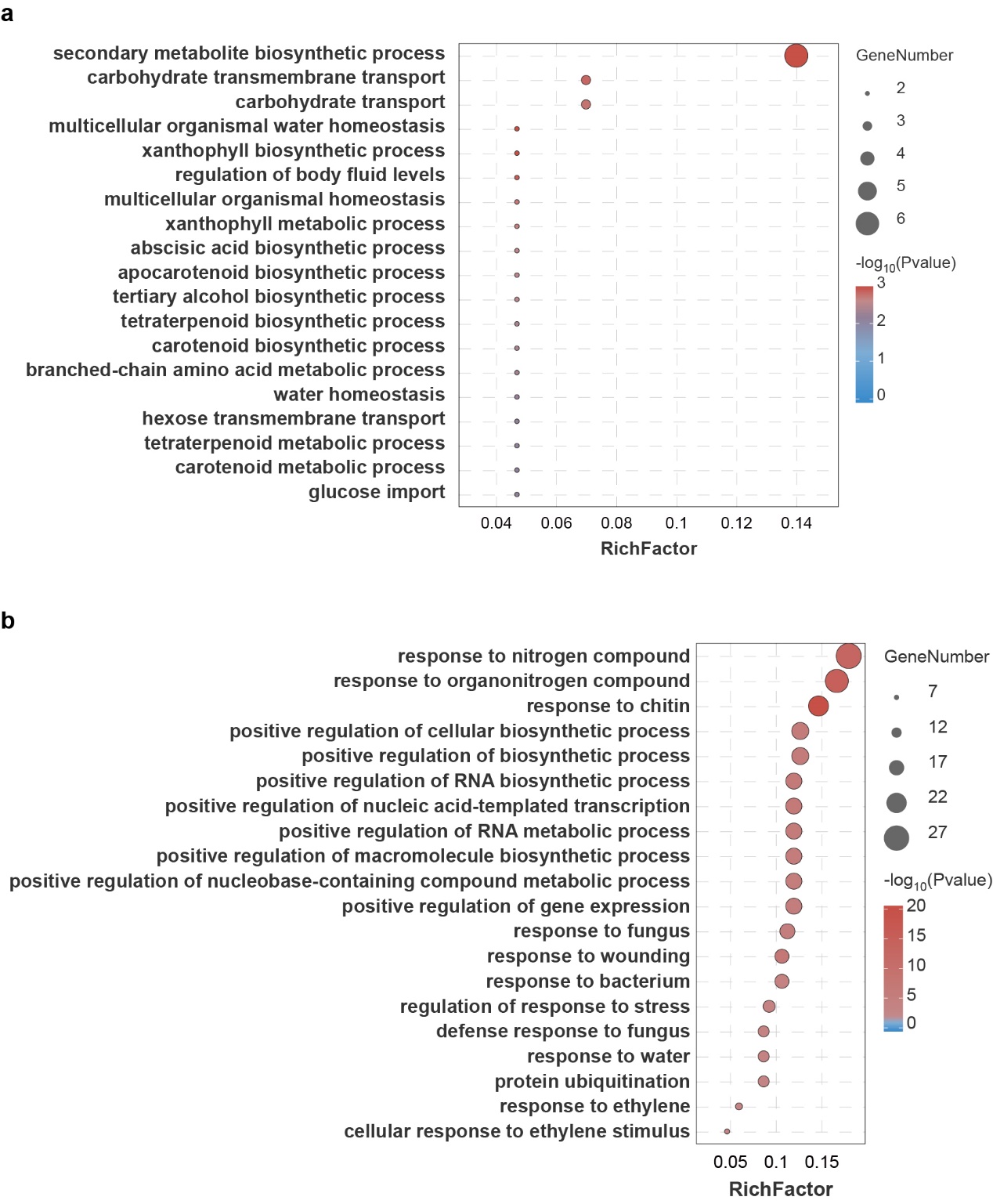
**

**Figure S47. Gene function enrichment of gene modules early responded to KClO_3_ treatment.** a. MEpurple, b. MEbrown. Only the top 20 GO terms of biological processes (BP) and KEGG pathways are shown.

**
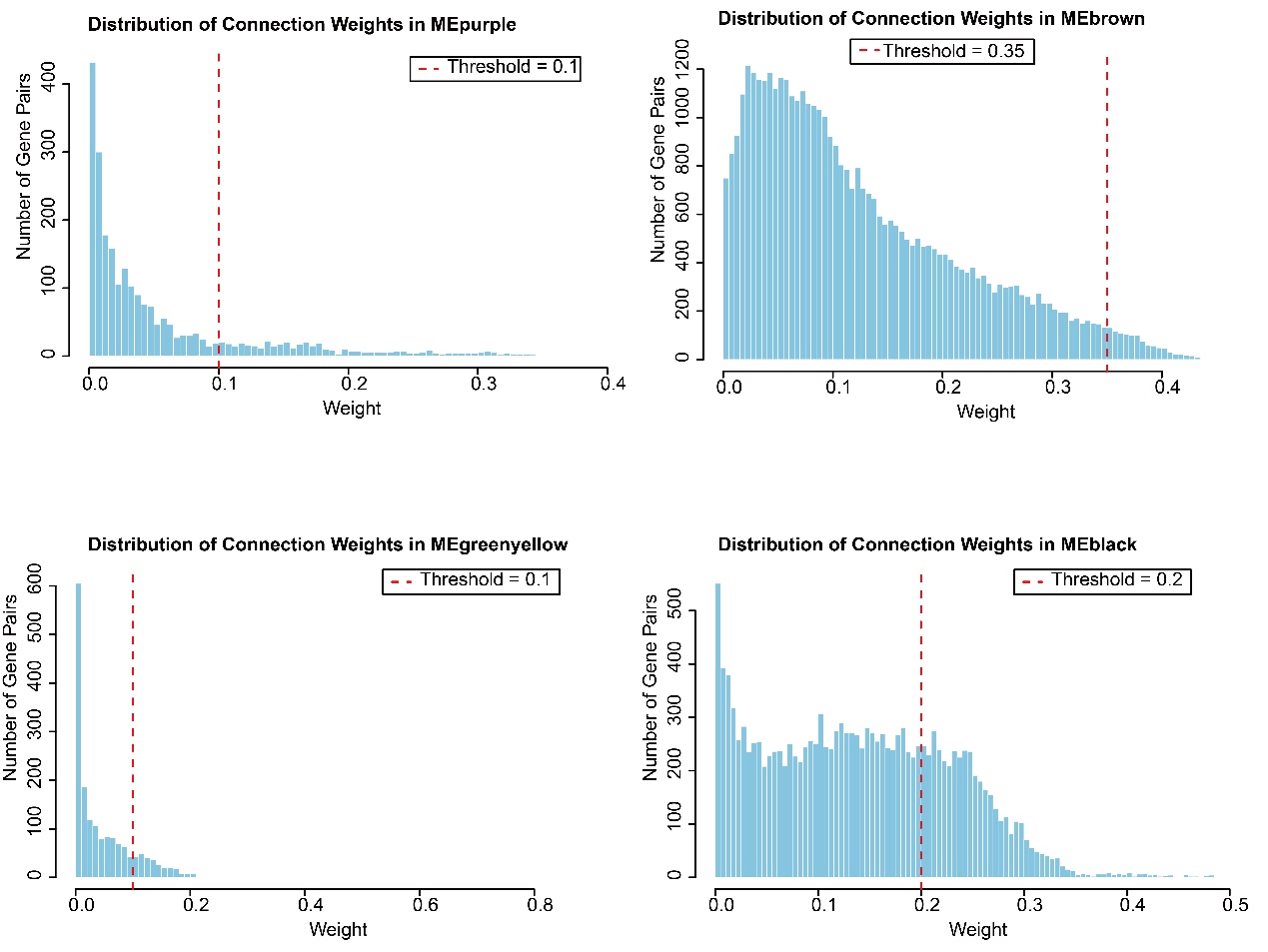
**

**Figure S48. Distribution of connection weights among co-expressed gene pairs in KClO3-induced flowering-specific modules.** Red dashed lines indicate the module-specific thresholds used for constructing gene co-expression networks.**
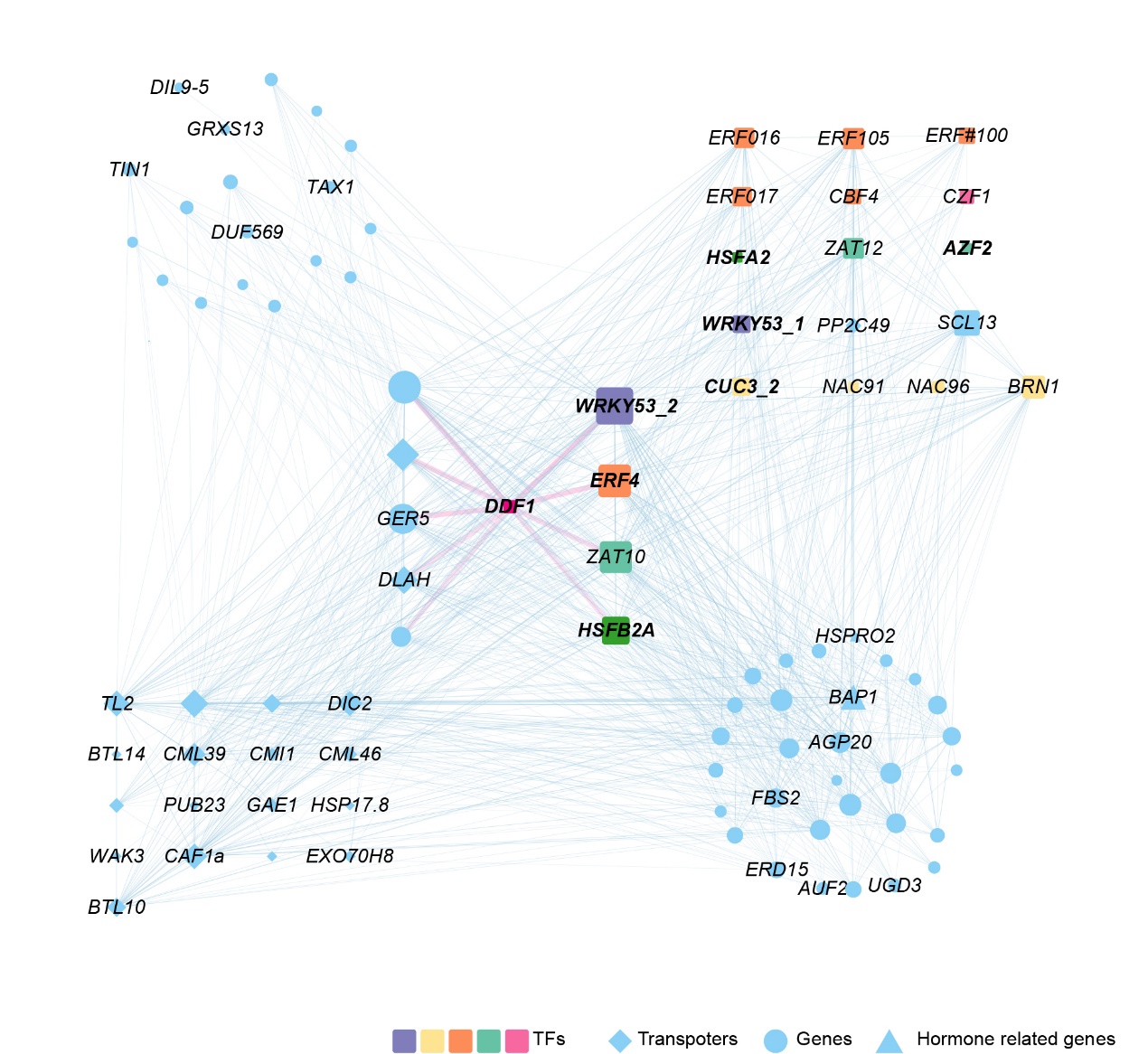
**

**Figure S49. Gene co-expression network of MEbrown.** Weight > 0.35, 92 genes.

**
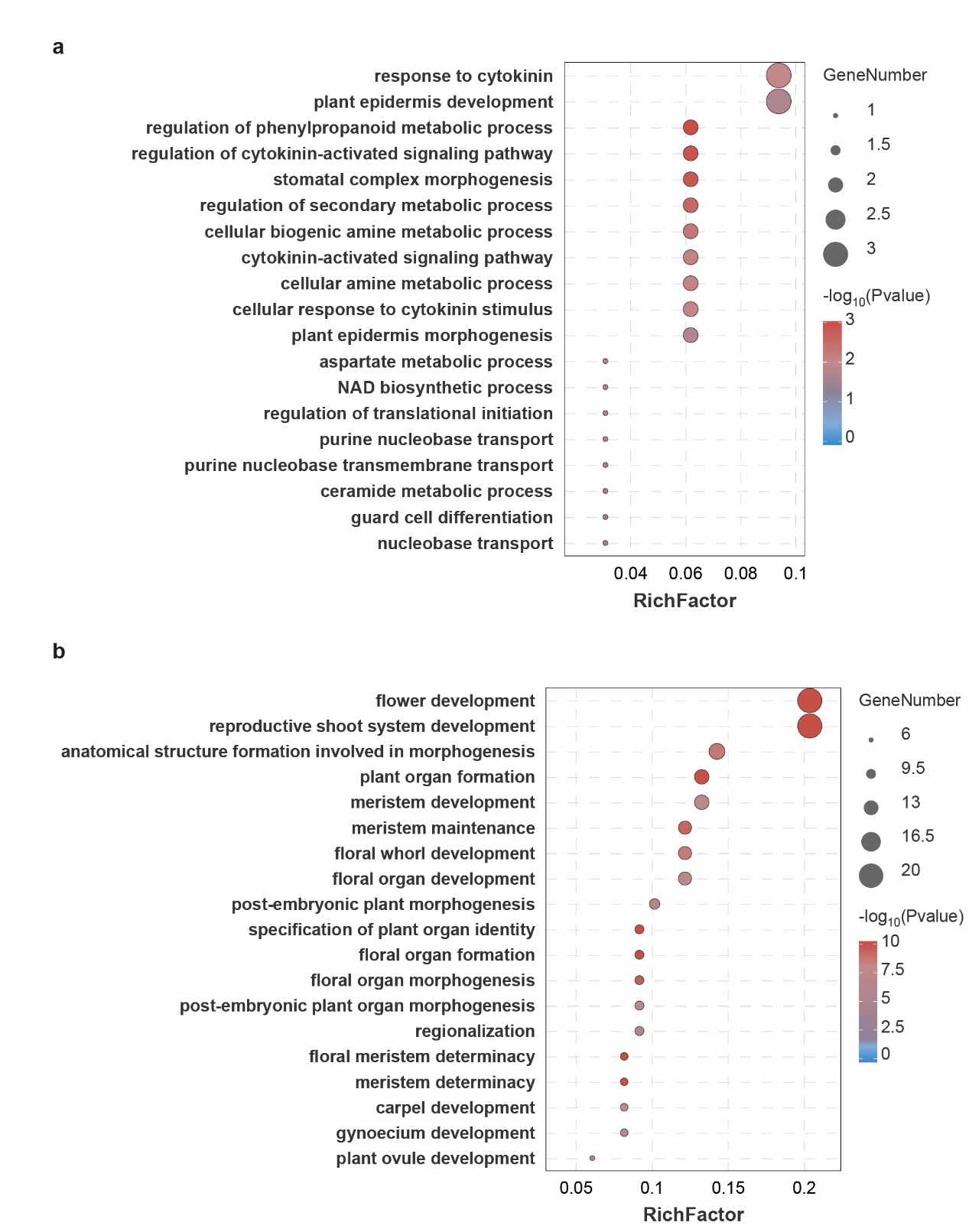
**

**Figure S50. Gene function enrichment of gene modules involved in floral initiation and flower bud differentiation under KClO_3_ treatment.** a. MEgreenyellow, b. MEblack. Only the top 20 GO terms of biological processes (BP) and KEGG pathways were shown.


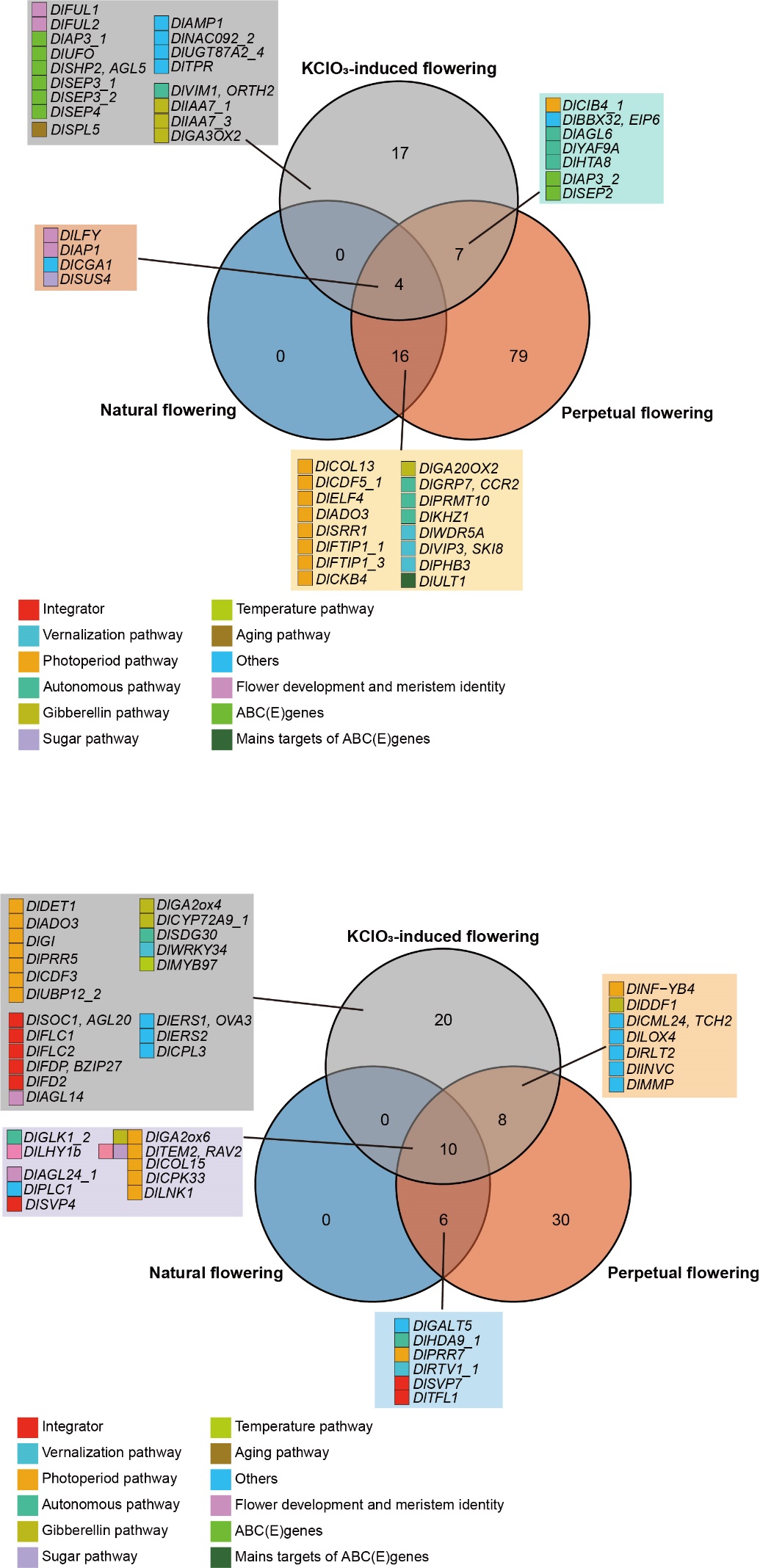


**Figure S51. Venn diagram of flowering time-related up-regulated DEGs for KClO_3_-induced off-season flowering, natural flowering and perpetual flowering.**

**
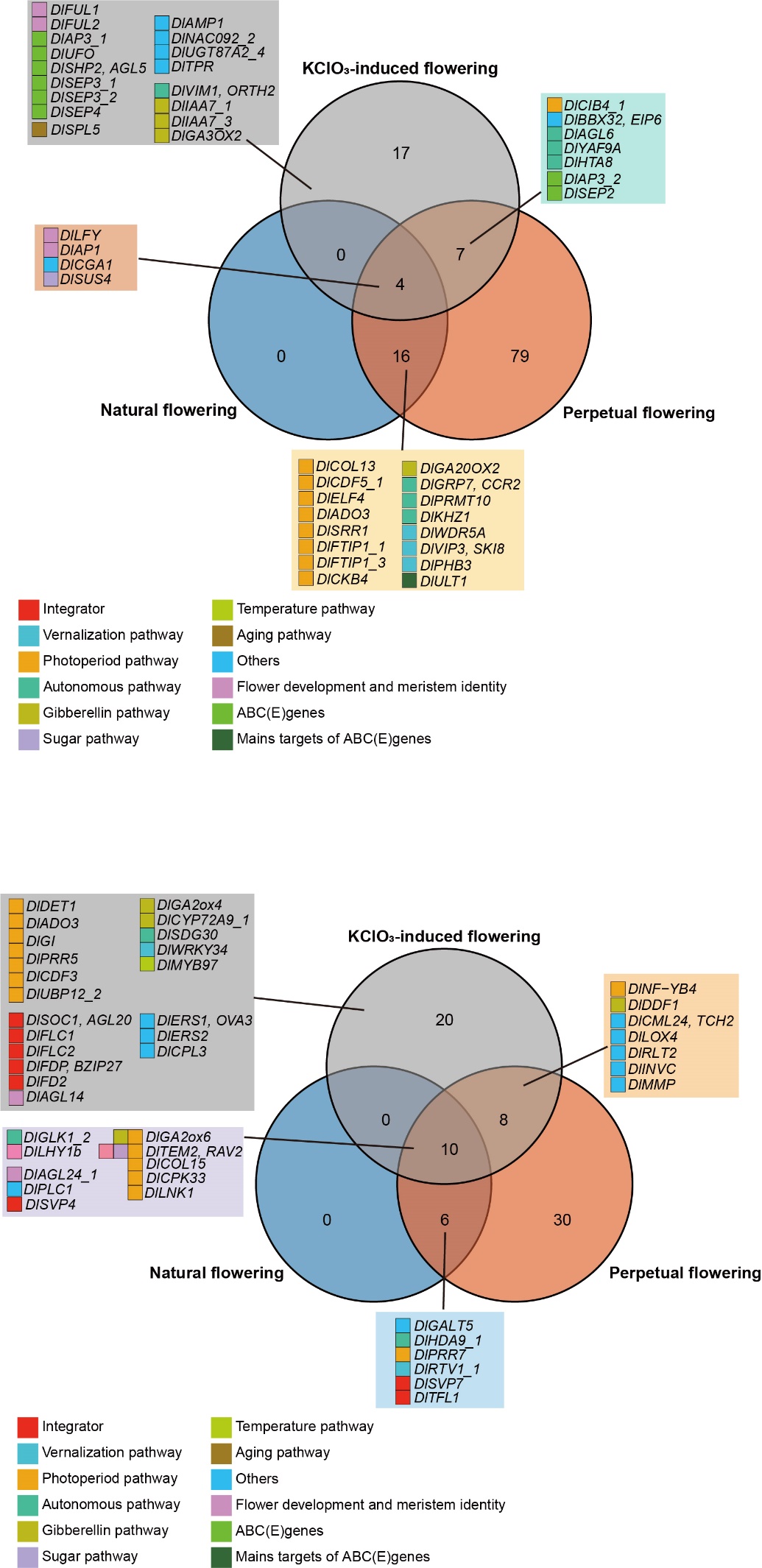
**

**Figure S52. Venn diagram of flowering time-related down-regulated DEGs for KClO_3_-induced off-season flowering, natural flowering and perpetual flowering.**

**
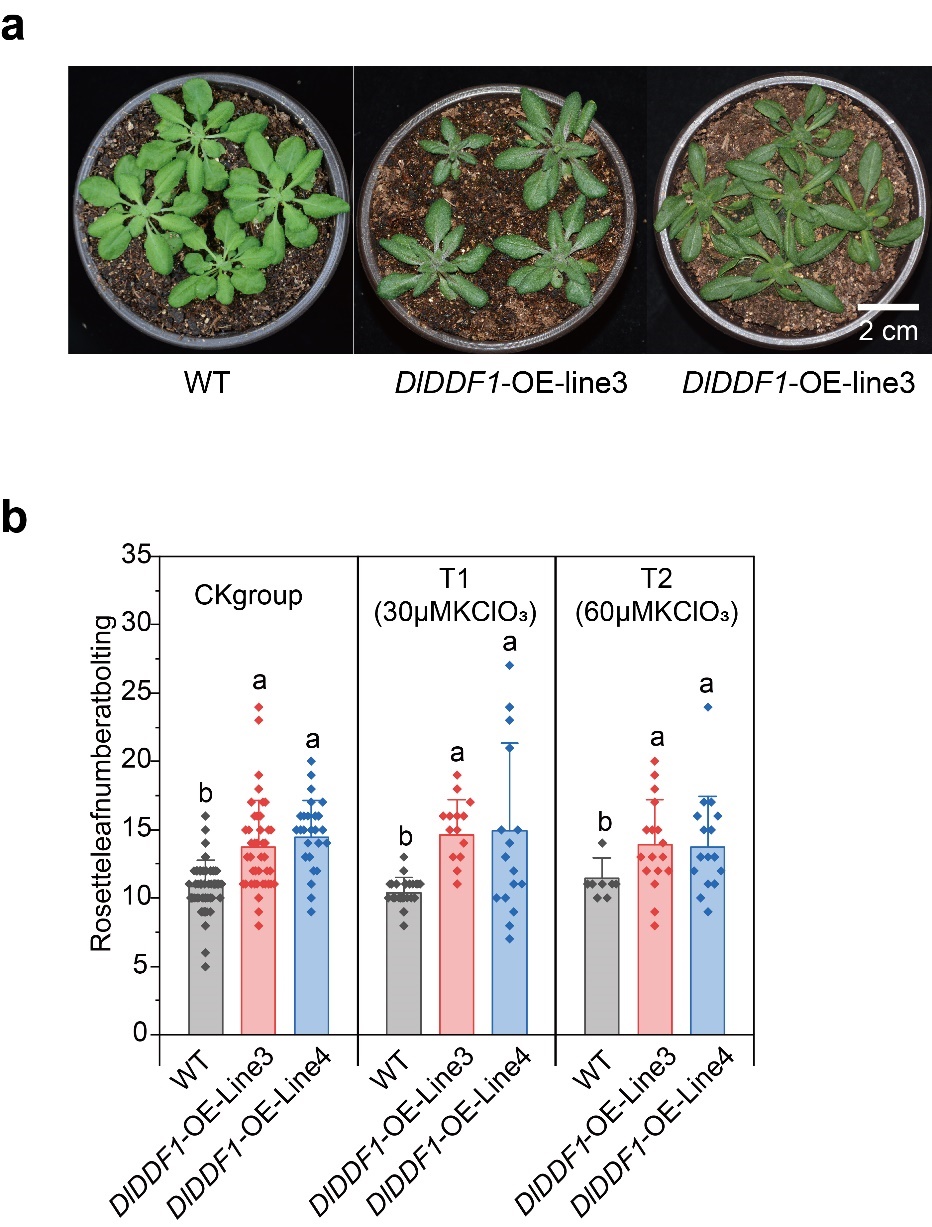
**

**Figure S53. Rosette leaf analysis of *DlDDF1* overexpression plants. a.** Morphological characteristics of rosette leaves in WT, *DlDDF1*-OE-line3, and *Dl DDF1*-OE-line4. Both overexpression lines exhibited deep-green leaves, slightly purple petioles, and leaf blades curled toward the abaxial side, forming a rod-like shape. **b.** Statistical analysis of rosette leaf number at the bolting stage under different treatments and genetic backgrounds. Sample size *n* > 10 for each group. Data were analyzed by two-way ANOVA, and multiple comparisons were performed using Tukey’s test. Different letters indicate significant differences at *P* < 0.05.


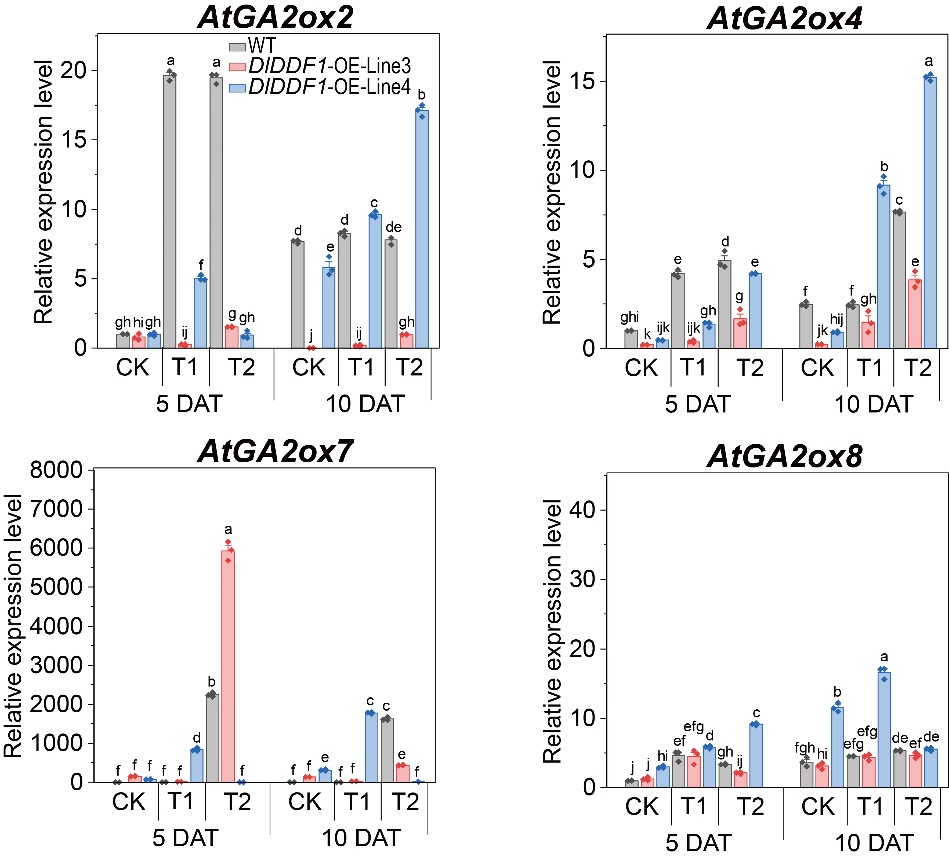


**Figure S54. Relative expression levels of genes in different genotypes under KClO_3_ treatments determined by qRT-PCR.** Gray, pink, and blue bars represent WT, *DlDDF1*-OE-line3, and *DlDDF1*-OE-line4, respectively. T1 indicates treatment with 30 μM KClO_3_, and T2 indicates treatment with 60 μM KClO_3_. “5 DAT” and “10 DAT” refer to 5 and 10 days after KClO₃ treatment. According to three-way ANOVA followed by Tukey’s multiple comparison test, different letters indicate significant differences at *P* < 0.05.

**
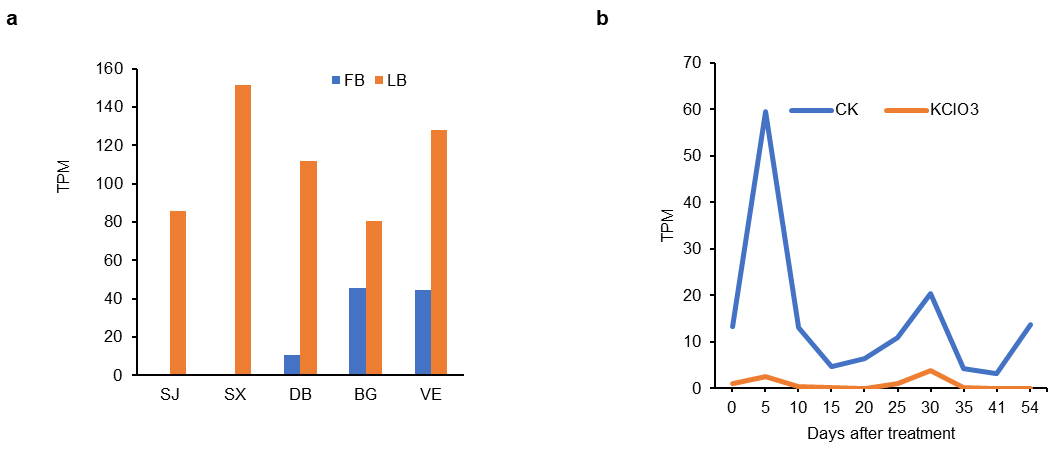
**

**Figure S55 Expression pattern of *DlDDF1* in natural and KClO_3_-induced flowering. a.** Expression pattern of *DlDDF1* in flower bud and leaf bud of five cultivars under natural floral transition conditions. **b.** Expression pattern of *DlDDF1* in buds at 10 time-points of CK and KClO_3_ treatment.

**Supporting tables: Table S1 ~ S25**

**Table S1. Sequencing data for genome assembly of** ***D. longan* cultivar ‘Shixia’.**

| Read type | Read length(bp) | Number of reads | Size (Gbp) | Base coverage |
| --- | --- | --- | --- | --- |
| PacBio HiFi reads | 16,682* | 1,334,129 | 22.3 | 51× |
| 150 bp paired-end Hi-C reads | 150* | 588,914,642 | 88.1 | 200× |

***Note: Average length**

**Table S2. Statistics of the genome assembly for** ***D. longan* cultivar ‘Shixia’.**

| **Assembly characteristics** | **Statistic** |
| --- | --- |
| **Assembly size (bp)** | 441,516,848 |
| **Number of scaffolds** | 95 |
| **Scaffold N50 (bp)** | 28,343,314 |
| **Scaffold N90 (bp)** | 230,311,93 |
| **Longest Scaffold (bp)** | 45,795,559 |
| **Number of contigs** | 98 |
| **Contig N50 (bp)** | 28,343,314 |
| **Contig N90 (bp)** | 21,907,183 |
| **Longest contig (bp)** | 42,987,119 |
| **Ratio of anchored onto the pseudochromosomes (%)** | 99.17 |
| **Ratio of oriented contigs (%)** | 94.70 |
| **Repeat region % of assembly (%)** | 50.23 |
| **Number of predicted gene models** | 29,325 |
| **Average coding sequence length(bp)** | 1,244 |
| **Average exons per gene** | 5 |

**Table S3. Statistics of contig-level assembly.**

| Items | HiFiasm assembly |
| --- | --- |
| Assembly size (bp) | 441,513,848 |
| No. of contigs | 98 |
| Maximum length (bp) | 42,987,119 |
| N90 (bp) | 21,907,183 |
| N80 (bp) | 23,290,233, |
| N70 (bp) | 25,914,415 |
| N60 (bp) | 26,975,426 |
| N50 (bp) | 28,343,314 |
| Average length (bp) | 45,052,43 |

**Table S4. Statistics for Hi-C sequencing and mapping analysis.**

| **Statistics of mapping** | |
| --- | --- |
| **Clean Paired-end Reads** | 294,457,321 |
| **Unmapped Paired-end Reads** | 44,054,771 |
| **Unmapped Paired-end Reads Rate (%)** | 14.961 |
| **Paired-end Reads with Singleton** | 186,765,723 |
| **Paired-end Reads with Singleton Rate (%)** | 63.427 |
| **Multi Mapped Paired-end Reads** | 14,078,939 |
| **Multi Mapped Ratio (%)** | 4.781 |
| **Unique Mapped Paired-end Reads** | 49,557,888 |
| **Unique Mapped Ratio (%)** | 16.83 |
| **Statistics for valid reads** | |
| **Unique Mapped Paired-end Reads** | 49,557,888 |
| **Dangling End Paired-end Reads** | 2,533,786 |
| **Dangling End Rate (%)** | 5.515 |
| **Self Circle Paired-end Reads** | 819,399 |
| **Self Circle Rate (%)** | 1.653 |
| **Dumped Paired-end Reads** | 18,693 |
| **Dumped Rate (%)** | 0.038 |
| **Interaction Paired-end Reads** | 36,270,356 |
| **Interaction Rate (%)** | 73.188 |
| **Lib Valid Paired-end Reads** | 22,909,658 |
| **Lib Valid Rate (%)** | 63.164 |
| **Lib Dup (%)** | 36.836 |

**Table S5. Statistics of Hi-C assembly analysis.**

| Group number | Anchored contigs | Length of contigs (bp) |
| --- | --- | --- |
| Group1 | 2 | 45,795,559 |
| Group2 | 1 | 34,905,494 |
| Group3 | 1 | 34,761,350 |
| Group4 | 2 | 34,444,747 |
| Group5 | 1 | 31,727,478 |
| Group6 | 1 | 29,143,437 |
| Group7 | 1 | 28,343,314 |
| Group8 | 1 | 27,962,133 |
| Group9 | 1 | 26,975,426 |
| Group10 | 2 | 26,106,781 |
| Group11 | 1 | 25,914,415 |
| Group12 | 1 | 23,527,950 |
| Group13 | 1 | 23,290,233 |
| Group14 | 1 | 23,031,193 |
| Group15 | 1 | 21,907,183 |
| Unanchored | 77 | 3,677,155 |
| Total | 95 | 441,513,848 |

**Table S6. BUSCO assessment of the ‘Shixia’ genome assembly.**

| **Description** | **Number**  **(****embryophyta)** | **Percentage (%)** | **Number**  **(****eudicotyledons)** | **Percentage (%)** |
| --- | --- | --- | --- | --- |
| **Complete BUSCOs (C)** | 1587 | 98.4 | 2280 | 98.0 |
| **Complete and single-copy BUSCOs (S)** | 1552 | 96.2 | 2229 | 95.8 |
| **Complete and duplicated BUSCOs (D)** | 35 | 2.2 | 51 | 2.2 |
| **Fragmented BUSCOs (F)** | 15 | 0.9 | 23 | 1.0 |
| **Missing BUSCOs (M)** | 12 | 0.7 | 23 | 1.0 |
| **Total BUSCO groups searched** | 1614 | 100 | 2,326 | 100 |

**Table S7. Consensus quality (QV) and completeness of the ‘Shixia’ genome based on Merqury analysis.**

| **Items** | **HiFi reads** |
| --- | --- |
| **Unique Kmer** | 1,299 |
| **Common Kmer for reads and genome** | 441,389,680 |
| **Consensus quality (QV)** | 68.0997 |
| **e-value** | 1.54894e-07 |
| **High-quality Kmer in genome** | 297,161,266 |
| **High-quality Kmer in reads** | 354,333,702 |
| **Completeness%** | 83.8648 |

**Table S8. Merqury quality values of the 15 chromosomes based on HiFi reads.**

| **Chromosome** | **Unique kmer** | **Common kmer for reads and genome** | **Quality value** | **Error rate** |
| --- | --- | --- | --- | --- |
| Chr01 | 83 | 45,794,523 | 70.2049 | 9.54E-08 |
| Chr02 | 167 | 34,443,711 | 65.9315 | 2.55E-07 |
| Chr03 | 36 | 34,905,476 | 72.6534 | 5.43E-08 |
| Chr04 | 39 | 34,761,332 | 72.2879 | 5.90E-08 |
| Chr05 | 77 | 31,727,460 | 68.937 | 1.28E-07 |
| Chr06 | 44 | 26,975,408 | 70.6627 | 8.58E-08 |
| Chr07 | 58 | 28,343,296 | 69.6778 | 1.08E-07 |
| Chr08 | 62 | 29,143,419 | 69.509 | 1.12E-07 |
| Chr09 | 119 | 26,105,745 | 66.1994 | 2.40E-07 |
| Chr10 | 36 | 27,962,115 | 71.6902 | 6.78E-08 |
| Chr11 | 7 | 25,914,397 | 78.472 | 1.42E-08 |
| Chr12 | 47 | 23,290,215 | 69.7383 | 1.06E-07 |
| Chr13 | 53 | 21,907,165 | 68.9506 | 1.27E-07 |
| Chr14 | 17 | 23,031,175 | 74.1062 | 3.88E-08 |
| Chr15 | 26 | 23,527,932 | 72.3536 | 5.82E-08 |

**Table S9. Genome consistency assessment.**

| **Items** | **HiFi reads** |
| --- | --- |
| **Number of reads** | 1,334,129 |
| **Data size (Gb)** | 22.26 |
| **Mapped bases (Gb)** | 22.25 |
| **Map rate (%)** | 99.93 |
| **Genome length (Mbp)** | 441 |
| **Mean depth** | 48.70 |
| **Coverage rate (%)** | 99.99 |

**Table S10. BUSCO assessment of protein-coding genome annotation.**

| **Description** | **Number**  **(plant)** | **Percentage** | **Number**  **(eudicotyledons)** | **Percentage** |
| --- | --- | --- | --- | --- |
| **Complete BUSCOs (C)** | 1,576 | 97.6 | 2,273 | 97.7 |
| **Complete and single-copy BUSCOs (S)** | 1,537 | 95.2 | 2,213 | 95.1 |
| **Complete and duplicated BUSCOs (D)** | 39 | 2.4 | 60 | 2.6 |
| **Fragmented BUSCOs (F)** | 24 | 1.5 | 27 | 1.2 |
| **Missing BUSCOs (M)** | 14 | 0.9 | 26 | 1.1 |
| **Total BUSCO groups searched** | 1,614 | 100 | 2,326 | 100 |

**Table S11. Genome assembly of ‘Shixia’ longan and comparison with other cultivars and related genera.**

| Species | *D. longan* | | | *L. chinensis* | *S. mukorossi* | *X. sorbifolia* | *N. lappaceum* |
| --- | --- | --- | --- | --- | --- | --- | --- |
| Cultivar* | **‘Shixia’** | **‘Jidanben’** | **‘Honghezi’** | **‘Feizixiao’** |  | **‘XsoG11’** | **‘Baoyan 7’** |
| Genome (M) | 441.5 | 455.5 | 471.9 | 470.4 | 391.6 | 489.2 | 328 |
| Scaffold number | 95 | 90 | 17,367 | 15 | 1007 | 417 | 24 |
| Scaffold N50 | 28.34 | 29.6 | 0.57 | 30.90 | 24.66 | 31.6 | 20.10 |
| Scaffold N90 | 23.03 | 22.3 | 0.12 | 24.39 | 15.75 |  | 18.47 |
| Scaffold Gaps | 3 | 229 | 34,025 | 1861 | 4931 |  | 1250 |
| Contig number | 98 | 250 | 51,392 | 2053 | - | 2,002 | 3,884 |
| Contig N50 | 28.34 | 12.1 | 0.03 | 1.13 | 2.88 | 31.60 | 0.33 |
| Contig N90 | 21.91 | 1.8 | 0.01 | - | - | - | - |
| Chromosomes (n) | 15 | 15 | 15 | 15 | 14 | 15 | 16 |
| Anchored onto chromosomes (%) | 99.19 | 98.70 | - | 94.93 | 90.87 | 96.2 | 99.39 |
| Number of genes | 29,325 | 40,420 | 31,007 | 31,896 | 31,853 | 35,039 | 26,500 |
| GC content (%) | 33.9 | 43.9 | 33.7 | 34.0 | 33.2 | 35.7 | 32.9 |
| Repeat content (%) | 54.96 | 55.40 | 52.34 | 55.48 | 50.95 | 63.47 | 44.62 |
| Gaps | 3 | 229 | 34,025 | - | - | - | - |
| Telomere number | 26 | - | - | - | - | - | - |
| Centromere number | 15 | - | - | - | - | - | - |
| BUSCO | 98.0 | 98.1 | 94.0 | 96.2 | 83.8 | 95.70 | 96.2 |
| LAI | 21.2 | 20.0 | 13.4 | 15.9 | 10.8 | 14.3 | 14.8 |
| QV | 68.1 | - | - | - | - | - | - |
| Coverage rate (%) | 99.97 | - | - | - | - | - | - |
| Reference | This study | (J. Wang *et al.*, 2022) | (Lin *et al.*, 2017) | (Hu *et al.*, 2022) | (Xue *et al.*, 2022) | (Wang *et al.*, 2023) | (Zhang *et al.*, 2021) |

**Table S12. Summary statistics of telomere prediction results of longan genome**

| **Chromosome** | **Length** | **Status** | **Left Num** | **Left Dir** | **Right Num** | **Right Dir** |
| --- | --- | --- | --- | --- | --- | --- |
| Chr01 | 45,795,559 | both | 1,350 | + | 1,623 | - |
| Chr02 | 34,444,747 | both | 1,621 | + | 2,227 | - |
| Chr03 | 34,905,494 | both | 1,358 | + | 1,075 | - |
| Chr04 | 34,761,350 | both | 1,377 | + | 1,812 | - |
| Chr05 | 31,727,478 | left | 1,548 | + | 0 |  |
| Chr06 | 26,975,426 | both | 1,857 | + | 1,369 | - |
| Chr07 | 28,343,314 | both | 1,741 | + | 1,964 | - |
| Chr08 | 29,143,437 | both | 1,409 | + | 641 | - |
| Chr09 | 26,106,781 | both | 1,069 | + | 1,800 | - |
| Chr10 | 27,962,133 | both | 1,954 | + | 1,606 | - |
| Chr11 | 25,914,415 | both | 2,132 | + | 619 | - |
| Chr12 | 23,290,233 | left | 885 | + | 0 |  |
| Chr13 | 21,907,183 | left | 1,342 | + | 0 |  |
| Chr14 | 23,031,193 | both | 1,259 | + | 1,930 | - |
| Chr15 | 23,527,950 | right | 0 |  | 1,428 | - |

* Telomere repeat monomer: AAACCCT

**Table S13. Statistical summary of the best predicted centromere candidates of longan genome**

| **Chromosome** | **Start** | **End** | **Length** |
| --- | --- | --- | --- |
| Chr01 | 26,833,405 | 27,026,609 | 193,205 |
| Chr02 | 20,199,631 | 20,377,053 | 177,423 |
| Chr03 | 683,793 | 843,072 | 159,280 |
| Chr04 | 72,496 | 318,358 | 245,863 |
| Chr05 | 14,082,495 | 14,223,093 | 140,599 |
| Chr06 | 16,532,800 | 16,636,221 | 103,422 |
| Chr07 | 1,712,509 | 1,819,703 | 107,195 |
| Chr08 | 20,590,566 | 20,741,822 | 151,257 |
| Chr09 | 6,061,722 | 6,214,882 | 153,161 |
| Chr10 | 16,484,816 | 16,639,420 | 154,605 |
| Chr11 | 8,677,207 | 8,975,748 | 298,542 |
| Chr12 | 22,595,462 | 23,288,756 | 693,295 |
| Chr13 | 20,339,209 | 20,752,743 | 413,535 |
| Chr14 | 16,772,982 | 16,903,359 | 130,378 |
| Chr15 | 1 | 508,498 | 508,498 |

**Table S14. Information on three remaining gaps of the longan genome**

| **Chromosome** | **Start** | **End** |
| --- | --- | --- |
| Chr01 | 2,807,441 | 2,808,440 |
| Chr02 | 1,717,787 | 1,718,786 |
| Chr09 | 15,294,954 | 15,295,953 |

**Table S15. Summary of genome annotation for longan cultivar ‘Shixia’.**

| **Annotation** | **number** | **Average size (bp)** | **Median size (bp)** | **Total length (Mb)** | **% of genome** | **% GC** |
| --- | --- | --- | --- | --- | --- | --- |
| Gene | 29,325 | 37,87 | 2,751 | 111 | 25 | 36 |
| Exons | 149,351 | 295 | 151 | 44 | 10 | 42 |
| Introns | 122,677 | 532 | 203 | 65 | 15 | 32 |
| miRNA | 137 | 118 | 111 | 0.0162 |  | 43 |
| snRNA | 885 | 111 | 107 | 0.0978 | 0.02 | 43 |
| tRNA | 437 | 75 | 73 | 0.0328 | 0.01 | 56 |
| rRNA | 1627 | 816 | 112 | 1.3278 | 0.3 | 55 |

**Table S16. Gene function annotation statistics.**

| **Type** | | **Number** | **Percent (%)** |
| --- | --- | --- | --- |
| Annotated proteins | NR | 28,069 | 95.72 |
|  | SwissProt | 21,460 | 73.18 |
|  | eggNOG | 27,983 | 95.42 |
|  | pFAM | 27,978 | 95.41 |
|  | KEGG | 9,084 | 30.98 |
|  | Mercator4 | 27926 | 95.23 |
|  | TAIR | 17873 | 60.95 |
|  | TCDB | 2133 | 7.27 |
|  | PlantTFDB | 1747 | 5.96 |
|  | iTAK | PKs: 1333  TFs: 1576  TRs:378 | 11.21 |
|  | FLOR-ID, PlantCFG, PFGD | 586 | 2.00 |
|  | total | 28,254 | 96.35 |
| Unannotated proteins | | 1072 | 3.65 |
| Total proteins | | 29,325 | 100 |

**Table S17. Summary of transposable elements (TEs) and other repeats in longan cultivar ‘Shixia’.**

| **Class** | **Group/Superfamily** | **Count** | **Length/bp** | **Percentage** |
| --- | --- | --- | --- | --- |
| Class I | LTR/*Copia* | 23,859 | 27,169,518 | 6.2% |
|  | LTR/*Gypsy* | 61,654 | 65,309,589 | 14.8% |
|  | LTR/unknown | 1,957 | 524,889 | 0.1% |
|  | LINE | 22,402 | 11,721,417 | 2.7% |
|  | SINE | 2,756 | 509,468 | 0.1% |
|  | pararetrovirus | 1,098 | 1,472,507 | 0.3% |
|  | PLE | 750 | 477,387 | 0.1% |
|  | nLTR/uknown | 67 | 23,956 | 0.0% |
|  | unknown | 2,843 | 917,589 | 0.2% |
| Class II | TIR/*CACTA* | 7,293 | 3,132,776 | 0.7% |
|  | TIR/*CMC* | 0 | 63,611 | 2.0% |
|  | TIR/*hAT* | 102,193 | 30,656,727 | 7.0% |
|  | TIR/*Kolobok* | 3,543 | 1,703,308 | 0.4% |
|  | TIR/*Merlin* | 138 | 17,060 | 0.0% |
|  | TIR/*MULE* | 19,541 | 10,806,983 | 2.5% |
|  | TIR/*Mutator* | 61,331 | 14,345,832 | 3.3% |
|  | TIR/*MITE* | 24,886 | 4,002,949 | 0.9% |
|  | TIR/*nMITE* | 29,812 | 8,092,226 | 1.8% |
|  | TIR/*P* | 1,295 | 223,630 | 0.1% |
|  | TIR/*PIF* | 18,212 | 5,183,286 | 1.2% |
|  | TIR/*PiggyBac* | 566 | 191,898 | 0.0% |
|  | TIR/*TcMar* | 22,316 | 5,770,847 | 1.3% |
|  | TIR/*Zator* | 253 | 75,176 | 0.0% |
|  | TIR/*Zisupton* | 3,076 | 2,003,554 | 0.5% |
|  | Crypton/*Crypton* | 1,128 | 179,239 | 0.0% |
|  | Helitron/*Helitron* | 12,409 | 5,743,531 | 1.3% |
|  | Maverick/*Maverick* | 1,047 | 477,245 | 0.1% |
|  | unknown | 14,053 | 4,378,120 | 1.0% |
| Unclassified |  | 78,216 | 28,499,264 | 6.5% |
| Satellite |  | 396 | 168,851 | 0.0% |
| Total repeat |  |  | 242,548,429 | 55.0% |

**Table S18. Chromosome size statistics of five closely related species.**

| **Species** | ***D. longan*** | | ***L. chinensis*** | | ***N. lappaceum*** | | ***S. mukorossi*** | | ***X. sorbifolia*** | |
| --- | --- | --- | --- | --- | --- | --- | --- | --- | --- | --- |
| **Chromosome** | **Length (bp)** | **Percent (%)** | **Length (bp)** | **Percent (%)** | **Length (bp)** | **Percent (%)** | **Length (bp)** | **Percent (%)** | **Length(bp)** | **Percent (%)** |
| Chr01 | 45,795,559 | **10.46%** | 49,720,996 | **10.57%** | 29,067,666 | 8.51% | 44,803,740 | **12.44%** | 40,097,451 | 8.39% |
| Chr02 | 34,444,747 | 7.87% | 39,913,022 | 8.49% | 26,802,709 | 7.85% | 35,436,268 | 9.84% | 39,341,037 | 8.24% |
| Chr03 | 34,905,494 | 7.97% | 36,721,797 | 7.81% | 25,038,929 | 7.33% | 31,626,953 | 8.78% | 35,738,308 | 7.48% |
| Chr04 | 34,761,350 | 7.94% | 34,608,567 | 7.36% | 23,844,088 | 6.98% | 27,111,013 | 7.53% | 34,590,148 | 7.24% |
| Chr05 | 31,727,478 | 7.25% | 34,400,222 | 7.31% | 23,286,198 | 6.82% | 26,892,049 | 7.46% | 34,015,926 | 7.12% |
| Chr06 | 26,975,426 | 6.16% | 32,069,177 | 6.82% | 21,703,535 | 6.36% | 25,300,362 | 7.02% | 34,973,130 | 7.32% |
| Chr07 | 28,343,314 | 6.47% | 30,902,938 | 6.57% | 20,311,494 | 5.95% | 24,843,121 | 6.90% | 32,173,403 | 6.74% |
| Chr08 | 29,143,437 | 6.66% | 30,266,306 | 6.43% | 20,104,206 | 5.89% | 24,664,527 | 6.85% | 32,613,457 | 6.83% |
| Chr09 | 26,106,781 | 5.96% | 30,124,626 | 6.40% | 20,088,722 | 5.88% | 21,895,847 | 6.08% | 30,062,096 | 6.29% |
| Chr10 | 27,962,133 | 6.39% | 28,140,600 | 5.98% | 19,520,659 | 5.72% | 21,883,516 | 6.07% | 29,817,396 | 6.24% |
| Chr11 | 25,914,415 | 5.92% | 26,228,562 | 5.58% | 19,041,425 | 5.58% | 21,615,186 | 6.00% | 28,876,547 | 6.05% |
| Chr12 | 23,290,233 | 5.32% | 24,682,403 | 5.25% | 19,029,923 | 5.57% | 21,131,190 | 5.87% | 28,800,770 | 6.03% |
| Chr13 | 21,907,183 | 5.00% | 24,517,789 | 5.21% | 18,796,286 | 5.50% | 17,295,268 | 4.80% | 28,934,939 | 6.06% |
| Chr14 | 23,031,193 | 5.26% | 24,387,438 | 5.18% | 18,789,777 | 5.50% | 15,759,200 | 4.37% | 25,069,408 | 5.25% |
| Chr15 | 23,527,950 | 5.37% | 23,684,157 | 5.04% | 18,469,584 | 5.41% |  |  | 22,556,286 | 4.72% |
|  |  |  |  |  | 17,596,462 | 5.15% |  |  |  |  |
| Average | 29,189,113 | 6.67% | 31,357,907 | 6.67% | 21,343,229 | 6.25% | 25,732,731 | 7.14% | 31,844,020 | 6.67% |
| Total | 437,836,693 | 100.00% | 470,368,600 | 100.00% | 341,491,663 | 100.00% | 360,258,240 | 100.00% | 477,660,302 | 100.00% |

**Table S19. Identification of longan specific genes compared with lychee.**

| **Gene family cluster** | | |
| --- | --- | --- |
| **Species** | **Longan** | **Lychee** |
| Total genes | 29,325 | 31,896 |
| Common orthologues groups | 16,898 | 16,898 |
| Orthologues groups genes | 24,359 | 25,223 |
| Orthologues | 24,079 | 25,078 |
| Specific orthologues groups | 486 | 869 |
| Specific genes | 3,522 | 3,866 |
| Unassigned Groups | 1,444 | 2,808 |
| Unassigned Genes | 1,444 | 2,808 |
| **BlastP** | | |
| **Species** | **DB-longan** | **Query-lychee** |
| Best-hit | 27,654 | 29,451 |
| Specific | 1,671 | 2,445 |
| **Species** | **Query-longan** | **DB-lychee** |
| Best-hit | 27,790 | 29,370 |
| Specific | 1,535 | 2,526 |
| **JCVI** | | |
| **Species** | **longan** | **lychee** |
| Anchors | 18,249 | 18,541 |
| Specific | 11,076 | 13,355 |

**Table S20. Statistics of NBS-encoding genes in longan and other four species of closely related genera of Sapindaceae family.**

| **Species** | **Cultivar** | **Total number of predicted genes** | **Total number of predicted NBS encoding genes** | **Ratio of NBS in the whole genome** |
| --- | --- | --- | --- | --- |
| *D. longan* | ‘Shixia’ | 29,325 | 389 | 1.3% |
|  | ‘Honghezi’ | 31,007 | 533 | 1.7% |
|  | ‘Jidanben’ | 40,420 | 594 | 1.5% |
| *L. chinensis* | ‘Feizixiao’ | 31,896 | 273 | 0.9% |
| *N. lappaceum* | ‘Baoyan7’ | 26,500 | 171 | 0.7% |
| *S. mukorossi* | - | 31,853 | 165 | 0.5% |
| *X. sorbifolia* | ‘XsoG11’ | 35,039 | 177 | 0.5% |

**Table S21. Distribution of NBS-encoding genes on the 15 pseudo-chromosomes of longan.**

| **Pseudomolecule name** | **NBS number** | **NBS (%)** | **Genes on pseudomolecule** | **NBS on each Pseudomolecule (%)** |
| --- | --- | --- | --- | --- |
|  |  |  |  |  |
| Chr1 | 11 | 2.83 | 3,336 | 0.33 |
| Chr2 | 121 | 31.11 | 1,953 | 6.20 |
| Chr3 | 75 | 19.28 | 1,890 | 3.97 |
| Chr4 | 1 | 0.26 | 2,501 | 0.04 |
| Chr5 | 51 | 13.11 | 1,941 | 2.63 |
| Chr6 | 15 | 3.86 | 1,776 | 0.84 |
| Chr7 | 16 | 4.11 | 1,936 | 0.83 |
| Chr8 | 6 | 1.54 | 1,631 | 0.37 |
| Chr9 | 12 | 3.08 | 1,819 | 0.66 |
| Chr10 | 28 | 7.20 | 1,510 | 1.85 |
| Chr11 | 21 | 5.40 | 1,840 | 1.14 |
| Chr12 | 11 | 2.83 | 1,584 | 0.69 |
| Chr13 | 0 | 0.00 | 1,784 | 0.00 |
| Chr14 | 13 | 3.34 | 1,888 | 0.69 |
| Chr15 | 3 | 0.77 | 1,795 | 0.17 |
| Unanchored scaffolds | 5 | 1.29 | 141 | 3.55 |
| **Total** | **389** | **100** | **29,325** | **1.33** |

**Table S22. Statistics of the chromosome structural variation types in longan compared with lychee.**

| Variation type^*^ | Counts | Counts percentage | Length | Genome percentage | Genes  distributed | TE  distributed |
| --- | --- | --- | --- | --- | --- | --- |
| SYN | 2,830 | 6.4 | 190,718,948 | 43.6 | 18,596 | 229,090 |
| SYNAL | 12,101 | 27.2 | 66,186,619 | 15.1 | 13,040 | 32,266 |
| NOTAL | 5,967 | 13.4 | 182,607,387 | 41.7 | 8,328 | 291,226 |
| HDR | 9,186 | 20.7 | 170,683,148 | 39.0 | 10,546 | 271,056 |
| INV | 182 | 0.4 | 55,354,335 | 12.6 | 3,000 | 77,669 |
| INVAL | 1,174 | 2.6 | 5,511,308 | 1.3 | 1,064 | 3,157 |
| INVDP | 1,286 | 2.9 | 3,622,257 | 0.8 | 508 | 2,926 |
| INVDPAL | 1,359 | 3.1 | 3,524,923 | 0.8 | 503 | 2,869 |
| INVTR | 462 | 1.0 | 1,559,016 | 0.4 | 250 | 1,419 |
| INVTRAL | 527 | 1.2 | 1,409,239 | 0.3 | 610 | 1,193 |
| DUP | 2,984 | 6.7 | 9,586,945 | 2.2 | 1,926 | 7,023 |
| DUPAL | 3,189 | 7.2 | 9,277,217 | 2.1 | 1,923 | 6,669 |
| CPG | 332 | 0.7 | 3,840,038 | 0.9 | 379 | 5,244 |
| TRANS | 903 | 2.0 | 3,042,503 | 0.7 | 618 | 2,776 |
| TRANSAL | 1,052 | 2.4 | 2,706,760 | 0.6 | 610 | 2,269 |
| CPL | 338 | 0.8 | 301,295 | 0.1 | 293 | 167 |
| DEL | 287 | 0.6 | 0 | 0.0 | 0 | 0 |
| INS | 312 | 0.7 | 860,180 | 0.2 | 230 | 1,235 |
| TDM | 7 | 1.57E-02 | 6,376 | 1.46E-03 | 7 | 4 |
| Total | 44,478 | 100 | 710,798,494 |  |  |  |

^*^ CPG, Copy gain in longan genome; CPL, Copy loss in longan genome; DEL, Deletion in longan genome; DUP , Duplicated region; DUPAL, Alignment in duplicated region; HDR, Highly diverged regions; INS, Insertion in longan genome; INV, Inverted region; INVAL, Alignment in inverted region; INVDP, Inverted duplicated region; INVDPAL, Alignment in inverted duplicated region; INVTR, Inverted translocated region; INVTRAL, Alignment in inverted translocated region; NOTAL, Un-aligned region; TDM, Tandem repeat; TRANS, Translocated region; TRANSAL, Alignment in translocated region; SYN, Syntenic region; SYNAL, Alignment in syntenic region.

**Table S23. Statistics of longan chromosome structural variations in longan and lychee.**

|  | **INV** | | | |  | **TRANS** | | | |  | **DUP** | | | |  |
| --- | --- | --- | --- | --- | --- | --- | --- | --- | --- | --- | --- | --- | --- | --- | --- |
|  | **Length** | **Regions** | **Genes** | **TEs** | **LTRs** | **Length** | **Regions** | **Genes** | **TEs** | **LTRs** | **Length** | **Regions** | **Genes** | **TEs** | **LTRs** |
| Chr01 | 854,196 | 17 | 70 | 1,102 | 198 | 335,704 | 89 | 62 | 272 | 99 | 850,639 | 242 | 177 | 566 | 151 |
| Chr02 | 6,902,768 | 18 | 446 | 9,271 | 1,424 | 417,055 | 126 | 75 | 461 | 68 | 1,054,121 | 361 | 210 | 1,055 | 200 |
| Chr03 | 6,217,209 | 14 | 500 | 7,784 | 1,224 | 263,604 | 100 | 47 | 285 | 54 | 871,343 | 325 | 157 | 972 | 184 |
| Chr04 | 13,757,032 | 5 | 721 | 19,695 | 4,390 | 196,634 | 21 | 39 | 154 | 23 | 838,419 | 129 | 153 | 573 | 88 |
| Chr05 | 6,514,914 | 17 | 328 | 9,720 | 1,638 | 283,339 | 105 | 74 | 289 | 47 | 786,708 | 317 | 173 | 726 | 133 |
| Chr06 | 606,515 | 14 | 49 | 85 | 258 | 247,182 | 81 | 50 | 227 | 35 | 710,362 | 239 | 179 | 536 | 86 |
| Chr07 | 3,604,134 | 9 | 119 | 5,116 | 1,816 | 288,199 | 57 | 58 | 250 | 68 | 608,114 | 176 | 121 | 356 | 100 |
| Chr08 | 3,703,213 | 16 | 140 | 5,631 | 1,626 | 101,325 | 44 | 23 | 73 | 25 | 411,002 | 131 | 88 | 280 | 69 |
| Chr09 | 405,517 | 11 | 20 | 750 | 108 | 211,752 | 82 | 53 | 216 | 59 | 594,470 | 216 | 103 | 451 | 150 |
| Chr10 | 1,241,819 | 18 | 74 | 1,776 | 467 | 147,961 | 49 | 27 | 121 | 54 | 556,512 | 183 | 120 | 365 | 109 |
| Chr11 | 1,152,055 | 6 | 53 | 1,777 | 421 | 144,681 | 37 | 29 | 108 | 14 | 712,795 | 182 | 115 | 332 | 79 |
| Chr12 | 1,482,726 | 14 | 87 | 1759 | 4,217 | 106,623 | 33 | 22 | 98 | 21 | 478,834 | 124 | 94 | 233 | 83 |
| Chr13 | 527,146 | 8 | 50 | 625 | 92 | 128,207 | 32 | 25 | 73 | 25 | 542,601 | 148 | 87 | 270 | 110 |
| Chr14 | 6,190,705 | 8 | 274 | 8,911 | 1,911 | 88,557 | 22 | 18 | 90 | 9 | 258,548 | 99 | 68 | 137 | 31 |
| Chr15 | 2,194,386 | 7 | 69 | 2,900 | 1,225 | 81,680 | 25 | 16 | 59 | 19 | 312,477 | 112 | 8 | 171 | 51 |
| Total | 55,354,335 | 182 | 3000 | 77,669 | 17,225 | 3,042,503 | 903 | 618 | 2,776 | 620 | 9,586,945 | 2,984 | 1,926 | 7,023 | 1,624 |

**Table S24. Statistics of transposable element numbers in INV, TRANS, and DUP regions of the longan genome.**

| **Class** | **Group/Superfamily** | **INV** | **TRANS** | **DUP** |
| --- | --- | --- | --- | --- |
| Class I | LTR/*Copia* | 3,615 | 408 | 1,048 |
|  | LTR/*Gypsy* | 13,155 | 206 | 555 |
|  | LTR/unknown | 354 | 5 | 14 |
|  | LINE | 3,646 | 80 | 151 |
|  | SINE | 341 | 9 | 19 |
|  | pararetrovirus | 160 |  |  |
|  | PLE | 86 | 1 | 7 |
|  | nLTR/uknown | 15 | 1 | 7 |
|  | Unknown | 369 | 17 | 37 |
| Class II | TIR/*CACTA* | 1,253 | 24 | 77 |
|  | TIR/*CMC* | 4,040 | 115 | 340 |
|  | TIR/*hAT* | 12,863 | 402 | 840 |
|  | TIR/*Kolobok* | 428 | 8 | 13 |
|  | TIR/*Merlin* | 7 |  | 1 |
|  | TIR/*MITE* | 2,898 | 86 | 238 |
|  | TIR/*MULE* | 2,760 | 56 | 134 |
|  | TIR/*Mutator* | 7,075 | 264 | 602 |
|  | TIR/nMITE | 3,818 | 197 | 456 |
|  | TIR/*P* | 142 | 12 | 24 |
|  | TIR*/PIF* | 2,180 | 121 | 352 |
|  | TIR/*PiggyBac* | 131 | 5 | 14 |
|  | TIR/*TcMar* | 3,214 | 57 | 126 |
|  | TIR/*Zator* | 13 | 1 | 3 |
|  | TIR/*Zisupton* | 486 | 13 | 57 |
|  | Crypton/*Crypton* | 100 | 4 | 4 |
|  | Helitron/*Helitron* | 2,101 | 195 | 545 |
|  | Maverick/*Maverick* | 103 | 16 | 23 |
|  | unknown | 1,587 | 85 | 222 |
| Unclassified |  | 10,899 | 389 | 904 |
| Satellite |  | 57 |  | 1 |
| Total repeat |  | 77,896 | 2,777 | 6,814 |

**Table S25. List of qRT-PCR primer sequences.**

| Gene | Primer |
| --- | --- |
| AtGA2OX7-F | GCCATCTAACTAGTGGTGAGGAGGT |
| AtGA2OX7-R | TCCCCACTCTTTCGCAGCT |
| AtGA2OX2-F | AGGTCGTAAACCACGGAGTC |
| AtGA2OX2-R | GATTAGCATTGAGGAGGAGATA |
| AtGA2OX4-F | ATCTGACGGCTGAGGGACTT |
| AtGA2OX4-R | AGAATTGATCGGACGGTGGA |
| AtGA2OX6-F | TTGAATCACTATCCACCAGC |
| AtGA2OX6-R | AGTCACCGACCAATACGAAG |
| AtGA2OX8-F | CGTCAGCCGTTTGATTGATG |
| AtGA2OX8-R | CTCGAAGCTCTCGCAATCG |
| AtGA20OX1-F | CCGCTCAAAATCCGTTCAAG |
| AtGA20OX1-R | CCTTCCCAAATGGCTGAAAC |
| AtGA20OX2-F | TGCTCACCGTTTGATGGAAAG |
| AtGA20OX2-R | CACCGGGTTTTCTCTGAGCTT |
| AtGA20OX3-F | CTCCAAGTCCCACTCATAGACCTAG |
| AtGA20OX3-R | TAGCCTCCGATGCCAAGC |
| AtWRKY75-F | ATGGGTCGTTGTATGCTCCTTT |
| AtWRKY75-R | ACGCTTTTCGAACAACCTTCTG |
| AtFT-F | CCTCAGGAACTTCTATACTTTGGTTATGG |
| AtFT-R | CTGTTTGCCTGCCAAGCTGTC |
| AtAP1-F | CAAGGTGACGAACCAAGTAT |
| AtAP1-R | ACGTAGTGTCGCATTTTAGG |
| AtDDF1-F | GTCTCTTTTTGTTTTCCGGCGA |
| AtDDF1-R | CTGGGTGACGTGTCTCCTTAAA |
| AtEF1α-F | GAGTACCCACCTTTGGGACG |
| AtEF1α-R | TTGGGTCCTTCTTGTCCACG |
| DlDDF1-F | ACTCAGACGAGGAGGTTTTG |
| DlDDF1-R | GTGTTTTCTTGTTCGGTTCA |
| AtLFY-F | CAAGGTGACGAACCAAGTAT |
| AtLFY-R | ACGTAGTGTCGCATTTTAGG |
